# Comparative Binding Studies of the Chelators Methylolanthanin and Rhodopetrobactin B to Lanthanides and Ferric Iron

**DOI:** 10.1002/cbic.202500312

**Published:** 2025-12-01

**Authors:** Sophie M. Gutenthaler‐Tietze, Michael Mertens, Manh Tri Phi, Patrick Weis, Björn Drobot, Alexander Köhrer, Robin Steudtner, Uwe Karst, N. Cecilia Martinez‐Gomez, Lena J. Daumann

**Affiliations:** ^1^ Department of Chemistry Ludwig Maximilians University of Munich Butenandtstraße 5–13 81377 Munich Germany; ^2^ Chair of Bioinorganic Chemistry Heinrich Heine University Düsseldorf Universitätsstr. 1 40225 Düsseldorf Germany; ^3^ Institute of Physical Chemistry Karlsruhe Institute of Technology Fritz‐Haber‐Weg 2 76131 Karlsruhe Germany; ^4^ Institute of Resource Ecology Biogeochemie Helmholtz‐Zentrum Dresden‐Rossendorf e.V. Bautzner Landstraße 400 01328 Dresden Germany; ^5^ Institute of Inorganic and Analytical Chemistry University of Münster Corrensstraße 48 48149 Münster Germany; ^6^ Department of Plant and Microbial Biology University of California–Berkeley California 94720 USA

**Keywords:** ion mobility spectrometry, lanthanide uptake, lanthanophores, metallophore, methylolanthanin, methylotrophy, siderophores, single‐cell inductively coupled plasma mass spectrometry, spectroscopy, time‐resolved laser‐induced fluorescence spectroscopy

## Abstract

The question of lanthanide (Ln) uptake in Ln‐using bacteria has gained a lot of attention in recent years, and the existence of specific Ln‐binding metallophores, termed lanthanophores, has been postulated. Here, the recently isolated metallophore methylolanthanin (MLL), which is shown to be involved in Ln metabolism of *Methylobacterium extorquens* AM1 along the structurally related siderophore rhodopetrobactin B (RPB B), is investigated. The total synthesis of both chelators as well as Ln‐binding investigations employing a multitude of spectroscopic methods is reported. Compared to MLL, RPB B has a higher binding affinity for Fe^3+^. Unexpectedly, both metallophores seem to precipitate Lns under biologically relevant conditions (pH and concentration range). Therefore, a solubility product of −12.07 ± 0.24 mol^2^ L^−2^ for the precipitated Eu^3+^‐MLL complex is reported. Furthermore, a combination of single‐cell inductively coupled plasma mass spectrometry and Liquid Chromatography‐Mass Spectrometry (LC‐MS) analysis of bacterial supernatant to investigate the Nd accumulation as well as MLL secretion under Fe limitation in *M. extorquens* AM1 is used. Finally, ion mobility spectrometry‐mass spectrometry and quantum chemical calculations are used to investigate the RPB B and MLL complexation in the gas phase with Fe^3+^ and all rare earth elements (except Pm). The results challenge the classical siderophore‐like Ln uptake (via simple solubilization) through MLL and underline again a potential complex interplay between Fe^3+^ and Ln^3+^ in microbial Ln uptake.

## Graphical abstract

The synthesis and the investigation of the lanthanide (Ln) and Fe‐binding properties of the putative lanthanophore methylolanthanin (MLL) and the siderophore rhodopetrobactin B (RPB B) are reported. While both chelators bind Lns, they unexpectedly precipitate with Lns under biologically relevant conditions and thereby challenge the proposed classic siderophore‐like Ln uptake.

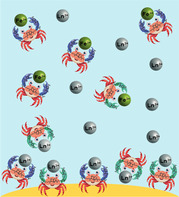

## 1 Introduction

The group of rare earth elements (REEs), consisting of the 15 lanthanides (Lns) lanthanum to lutetium and the elements scandium and yttrium, has long been believed to be biologically irrelevant. In nature, the REEs usually coappear in hardly soluble minerals such as bastnäsite, xenotime, and monazite.^[^ ^1^ ^]^ The perception that these elements are not used in biological processes was primarily due to their limited bioavailability under physiological conditions and the misconception that they are rare.^[^ ^2^ ^,^ ^3^ ^]^ However, this belief was severely challenged approximately a decade ago, as the first studies showed that these metals indeed play an important role for the metabolism of methylotrophic bacteria.^[^ ^4^, ^5^ ^–^ ^6^ ^]^ Since then, the field of Ln‐dependent bacterial metabolism flourished, and not only were numerous organisms using Lns in their enzymes identified,^[^ ^7^ ^,^ ^8^ ^]^ but also the first Ln‐binding proteins were discovered.^[^ ^9^, ^10^, ^11^ ^–^ ^12^ ^]^ However, one long‐standing hypothesis that remained unconfirmed for years was how mesophilic Ln‐using bacteria acquire the poorly bioavailable Lns in the first place.^[^ ^13^ ^]^

That a TonB‐dependent reporter might be associated with Ln uptake was suggested in multiple studies,^[^ ^9^ ^,^ ^14^ ^]^ and in 2019, Ochsner et al. demonstrated that the Ln‐dependent growth of *Methylorubrum extorquens* PA1, a methylotrophic bacterium, was linked to a TonB‐dependent receptor and a specific ABC transporter.^[^ ^15^ ^]^ This gave rise to the hypothesis that Ln‐using bacteria might use metallophores, specifically designed for Lns, termed lanthanophores,^[^ ^8^ ^]^ for Ln uptake.

Metallophores, by definition, are low‐molecular‐weight (typically <1500 Da) molecules which are secreted as secondary metabolites by bacteria, fungi, or plants to act as metal chelators and scavenge different metals from the environment.^[^ ^16^ ^,^ ^17^ ^]^ To date, multiple metallophores are known for various trace elements such as Cu, Zn, Mo, V, Ni, and Fe.^[^ ^17^ ^]^ However, the most extensively studied class of metallophores are siderophores, molecules designed by nature to coordinate ferric iron. Since the first siderophores were discovered in the fifties,^[^ ^18^ ^,^ ^19^ ^]^ hundreds of siderophores have been described in the literature.^[^ ^20^ ^]^

Since the first suggestion that lanthanophores may exist, further studies suggesting siderophore‐like molecules for Ln uptake emerged for different microorganisms, also indicating that some bacteria might use siderophores for Fe and Ln acquisition.^[^ ^21^ ^,^ ^22^ ^]^ Studies have also shown that natural siderophores have Ln‐binding capabilities.^[^ ^23^ ^]^ Furthermore, it was also hypothesized that mechanisms might exist where microorganisms modify metallophores produced by other organisms for Ln binding.^[^ ^22^ ^]^ This is not far‐fetched from a coordination chemistry point of view. In particular, as siderophores have already been used widely as inspiration for the development of potent Ln and actinide (An) chelators such as hydroxypyridinones (HOPO)‐ and 2‐hydroxyisophthalamide (IAM)‐based chelators for, e.g., antenna ligands in luminescent Ln complexes.^[^ ^24^, ^25^ ^–^ ^26^ ^]^

**Figure 1:**
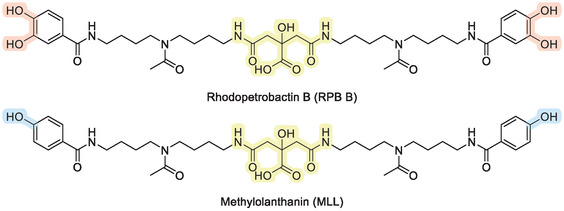
Molecular structure of the two citrate‐based chelators rhodopetrobactin B (RPB B)^[^ ^27^ ^]^ and methylolanthanin (MLL); the central citrate motif is highlighted in yellow, while the differences between both chelators are highlighted in red/blue.

Nevertheless, the identification of the first metallophore involved in Ln metabolism was only recently achieved. Zytnick et al. identified and isolated the metallophore methylolanthanin (MLL), from *Methylobacterium extorquens* (*M. extorquens*) AM1.^[^ ^27^ ^]^ MLL bears structural resemblance to the known siderophore rhodopetrobactin B (RPB B) (**Figure** **1**).^[^ ^28^ ^]^

However, it distinguishes itself by having two 4‐hydroxybenzoic acid moieties instead of the two 3,4‐dihydroxybenzoic acid moieties present in RPB B.^[^ ^27^ ^]^ The para‐position of the hydroxybenzoic acid moiety is especially interesting and unexpected as the phenolate‐type siderophores usually have hydroxy groups in the ortho‐position.^[^ ^29^ ^]^ After we reported initial binding experiments of MLL with La^3+^, Nd^3+^, and Lu^3+^ via direct‐injection mass spectrometry showing the formation of Ln complexes in the gas phase,^[^ ^27^ ^]^ we wanted to thoroughly investigate the Ln binding of MLL in solution. Here, we present the synthesis of MLL and RPB B as well as the investigation of both metallophores in respect to their metal‐binding behavior to Lns and ferric iron using a multitude of methods ranging from UV–vis spectroscopy, NMR spectroscopy, and time‐resolved laser‐induced fluorescence spectroscopy (TRLFS) to ion mobility spectrometry (IMS) time‐of‐flight mass spectrometry analysis complemented by quantum chemical calculations. While the binding investigation turned out to be more challenging than expected, valuable insights into the biological role of MLL were uncovered. As a connection between the Fe and Ln metabolism was already suggested previously,^[^ ^27^ ^]^ we also investigated the effect of Fe limitation on the Nd accumulation as well as on MLL secretion by single‐cell inductively coupled plasma mass spectrometry (scICP‐MS) in combination with Liquid Chromatography‐Mass Spectrometry (LC‐MS) analysis. Nd was chosen to stay consistent with the cultivation experiments described by Zytnick et al.^[^ ^27^ ^]^

## 2 Results and Discussion

Due to the structural resemblance of the newly identified MLL with RPB B, we wanted to investigate both metallophores for their Ln‐binding capabilities. For this, RPB B was synthesized based on the literature‐known synthesis of petrobactin by an 11‐step synthesis (Scheme S1, Supporting Information).^[^ ^30^ ^]^ First, the linker arm was synthesized starting from 1,4‐diaminobutane, yielding an acetylated dibutylenetriamine structure covalently attached to a 3,4‐dihydroxybenzoic acid (3,4‐DHBA) building block via an amide bond. Conjugation to the citrate core was achieved using *N,N′*‐dicyclohexylcarbodiimide (DCC) and *N‐*hydroxysuccinimide (NHS) as coupling agents. The final purified product was obtained after deprotection and purification via preparative high‐performance liquid chromatography. By a modified procedure, which involved using 4‐hydroxybenzoic acid as a building block instead of 3,4‐DHBA, we were able to obtain synthetic MLL. The analytical data of synthesized MLL are in excellent agreement with that of the isolated molecule previously published.^[^ ^27^ ^]^ This is, to the best of our knowledge, the first time that the syntheses of RPB B and MLL are reported. As the observed concentrations of MLL in the bacterial supernatant of *M. extorquens* AM1 consistently remained in the nanomolar range under the tested conditions (Figure 4B and S17, Supporting Information), making the isolation extremely cumbersome, the synthesis of MLL was an important step toward obtaining greater quantities of MLL for extensive binding studies.

### 2.1 Binding Studies via UV–Vis, NMR, and TRLFS

**Figure 2:**
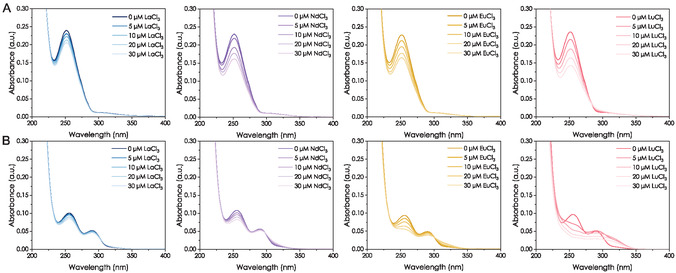
UV–vis spectra of Ln^3+^ to ligand A) 10 µM MLL and B) 10 µM RPB B titrations in MOPSO buffer (10 mM, 100 mM KCl, pH 6.0) with increasing amounts of Ln^3+^ (0 to 30 µM; Ln = La, Nd, Eu, and Lu from left to right).

In the case of MLL, we were curious to discover which groups are involved in Ln coordination. We anticipated to see via UV–vis spectroscopy whether the phenolate groups are involved in binding through the formation of an intensely colored charge–transfer complex. The experiments were performed at pH 6.0 to mimic the pH present in the bacterial supernatant during early stationary phase when grown in ½ Hypho medium and with La^3+^, Nd^3+^, Eu^3+^, and Lu^3+^. We chose these four Lns to have a selection of Lns that bacteria use (La^3+^ and Nd^3+^), can use but do not prefer (Eu^3+^) and do not use (Lu^3+^) for their C_1_‐metabolism. The ionic radii of Ln^3+^ decrease gradually within the series; this is known as the lanthanide contraction and has been shown to influence both bacterial growth and the activity of Ln‐dependent enzymes.^[^ ^6^ ^,^ ^31^, ^32^, ^33^, ^34^, ^35^ ^–^ ^35^ ^]^ Instead of an expected signal shift, we observed a continuous decrease of the absorption band at 251 nm with increasing Ln concentration (**Figure** **2**A). Only for the addition of Lu^3+^, an isosbestic point at 283 nm can be suspected. However, for all tested Lns, the observed absorbance can significantly be further decreased by centrifugation and measurement of the supernatant (Figure S6, Supporting Information). Thus, we attribute the spectral changes to the partial removal of MLL‐Ln^3+^ complex, likely formed by precipitation.

For analogously performed experiments with RPB B, we observe the literature‐known spectrum of the 3,4‐catechol ligand with maxima at 294 and 255 nm.^[^ ^28^ ^]^ After the addition of La^3+^, Nd^3+^, or Eu^3+^, both absorption bands decrease and isosbestic points can be observed at 304, 299, and 283 nm, respectively. For Nd^3+^ and Eu^3+^, a slight redshift of both maxima appears. At first glance, the results do not suggest the formation of a precipitate in contrast to the MLL experiments, but rather the formation of potentially soluble complexes, indicating also differences for the tested Lns. However, after centrifugation, the absorbance decreased significantly, again pointing to some amount of precipitate formation (Figure S7, Supporting Information). Interestingly, the spectral changes observed following the addition of Lu^3+^ do not align with the observations for La^3+^ and Nd^3+^. With addition of 0.5 equiv. Lu^3+^, a redshift and decrease of the signal at 255 nm and a broadening for the band at 294 nm occur, likely due the formation of a Lu^3+^ complex with involvement of the catechol groups.

We further investigated both chelators by La^3+^ titration experiments via ^1^H NMR spectroscopy which again indicated precipitation of a MLL‐La^3+^ complex (Figure S9, Supporting Information). For RPB B (Figure S11, Supporting Information), very similar observations can be made overall albeit the spectral changes suggest the presence of a small amount of soluble RPB B‐La^3+^ complex as well as possible precipitation of a complex.

However, how does this fit into the picture of a lanthanophore which gets secreted by the bacteria in order to solubilize poorly bioavailable Lns? In the bacterial supernatant, we only detect MLL in the nanomolar range (Figure 4B), while both experimental setups needed chelator concentrations in the lower micromolar range. Thus, we considered that the precipitation of the chelator might only occur at higher ligand concentrations. This could have a biological function for MLL such as avoiding the uptake of toxic amounts of Lns. Therefore, we conducted Ln affinity experiments at lower concentrations in order to get closer to biologically relevant conditions.

**Figure 3:**
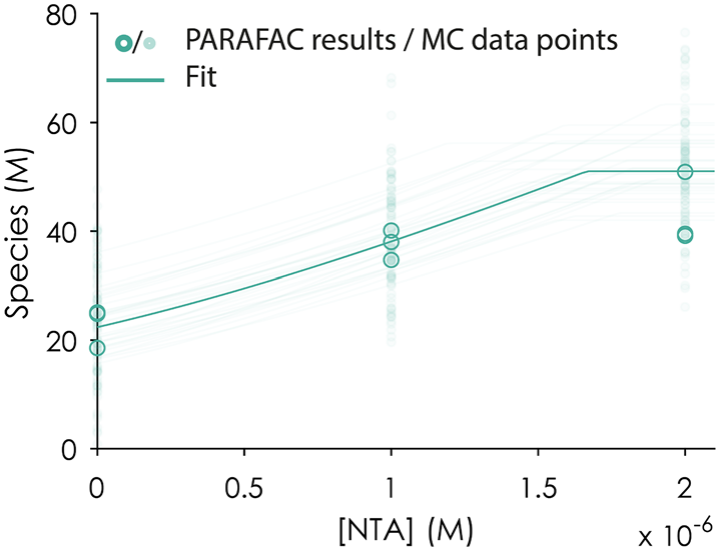
PARAFAC results and fit of the competition experiment between MLL, NTA, and Eu^3+^. The data points represent triplicates of three independent series containing fixed concentrations of MLL (2 µM) and EuCl_3_ (2 µM). The lines represent the fit as obtained from 100 MC runs. An extensive discussion of the experimental design, analysis workflow, and thermodynamic characterization is available in the Supporting Information in Section 2.4 on pages 39–42.

**Figure 4:**
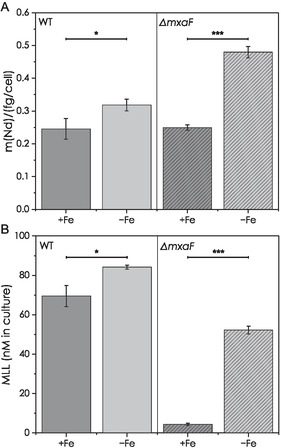
scICP‐MS quantification of Nd in WT and Δ*mxaF* cells grown under +/−Fe conditions (½ Hypho medium, 50 mM MeOH, 2 µM NdCl_3_) and MLL quantification in the corresponding supernatant samples. A) Bar plot of mean values of the median biological replicates. Differences between mean values for WT and *ΔmxaF* were checked for significance using a two‐sample t‐test (95% confidence interval of mean difference). Mean mass of Nd per cell was significantly higher under Fe‐limiting conditions for both WT (*t*(4) = 3.50258, *p* = 0.02484) and mutant (*t*(6) = 23.96897, *p* < 0.0001). Underlying distribution as boxplot is shown in Figure S19, Supporting Information. B) Bar plot of the determined MLL concentration adjusted by OD in WT and *ΔmxaF* supernatant samples. Differences between mean values for WT and *ΔmxaF* were checked for significance using a two‐sample t‐test (95% confidence interval of mean difference). The MLL concentration under Fe‐limiting conditions was for both WT (*t*(4) =  3.77627, *p* = 0.0195) and mutant (*t*(6) = 40.30977, *p* < 0.0001) significantly higher. For both graphs: *p < 0.05 and ***p < 0.001 applies.

To achieve this, we moved on to TRLFS, a method which can be used in the nanomolar range. With TRLFS, we can use the excellent luminescence properties of Eu^3+^ as well as the high quantum yield of the radioactive actinide Cm^3+^ as proxy for Lns in order to work at even lower concentrations. In the first titration experiments using 1 µM Eu^3+^, we made some unexpected observations. For example, the signal intensity of the observed Eu^3+^ species decreased with increasing MLL concentration. Usually, a higher quantum yield and thus better signal‐to‐noise ratio are expected at higher complex concentrations (due to displacement of quenching water molecules in the first coordination sphere of Eu^3+^). Furthermore, the observed lifetime of the formed Eu^3+^ complex showed no monoexponential decay, and we observed unusual scattering in the sample distributions along the titration series (Figure S13, Supporting Information). While the experiment demonstrated that MLL can bind Eu^3+^, we were not able to obtain reliable thermodynamic parameters and again assumed potential precipitation of a formed complex as cause. Still assuming that the concentration might be too high, we performed measurements with Cm^3+^ at a metal concentration of 100 nM and therefore in a range close to the observed MLL concentrations in the bacterial supernatant (Figure 4B). The data seemed very promising at first, although similar observations to the Eu^3+^ titration series were made (Figure S14, Supporting Information), again pointing toward issues with precipitation. For easier sample handling, we again moved to Eu^3+^ and lowered the concentration to 200 nM (despite longer measurement time and worse signal‐to‐noise ratios at low concentrations in comparison to Cm^3+^). After completion of the titration, we centrifuged the last titration step and could significantly remove Eu from the solution—thus again clearly suggesting the formation of particles and the precipitation of a Eu^3+^‐MLL complex (Figure S15, Supporting Information). The same experiment was performed with RPB B, leading to a similar result (Figure S16, Supporting Information). Since the formation of a precipitate at nanomolar concentrations cannot be observed by the naked eye, we used chemical microscopy^[^ ^36^ ^]^ to take a closer look at the presumably formed MLL‐Eu^3+^ precipitate. We were able to detect particles under the microscope showing the expected spectral features of complexed Eu^3+^, strongly supporting the formation of a MLL‐Eu^3+^ precipitate (Figure S17 and S18, Supporting Information). The formation of precipitates impedes the determination of reliable affinity constants. However, we were able to determine the solubility product of the precipitating Eu^3+^‐MLL complex by designing a competition experiment between Eu^3+^, MLL, and nitrilotriacetic acid (NTA) using TRLFS. As the Eu^3+^‐NTA system has been well characterized in the literature,^[^ ^37^ ^]^ and NTA seems to bind Lns in a similar affinity range, it is a good choice for such a setup. With this approach, we were able to determine the solubility product of the precipitated Eu^3+^‐MLL complex (K_SP_ = [Eu^3+^] × [MLL^3−^]) to be −12.07 ±  0.24 mol^2 ^L^−2^ (**Figure** **3**).

However, this did not resolve the questions that remain with the result that MLL precipitates with Lns within the nanomolar range in aqueous solution in a biologically relevant pH range which would be present in the bacterial supernatant (pH 5.5–6.8). What is the biological function of a chelator that precipitates Lns? We hypothesize that MLL might precipitate Lns to concentrate them in a certain form to build reservoirs for metal uptake and act as a sensor or could give a competitive advantage toward other microbes. We should also note that while we investigated MLL at a biological relevant pH, this does not fully represent the complex interplay of different chemical species within the bacterial culture medium during cultivation. Thus, we cannot fully rule out the possibility that MLL might solubilize Lns in a biological environment in the very low nanomolar or picomolar range. However, as RPB B, a known siderophore, shows a similar binding behavior, the observations strongly indicated, as already suggested earlier,^[^ ^27^ ^]^ a close relationship between the Fe and Ln metabolism. Zytnick et al. showed with promoter fusion experiments that the promoter activity for *mll* (refers to the gene cluster) increases under Fe‐limiting conditions for both WT (wild‐type) and the Ln‐dependent Δ*mxaF* mutant when grown Ln independently on the multicarbon source succinate, but even more when grown on the C_1_ carbon source methanol with NdCl_3_.^[^ ^27^ ^]^ Therefore, we performed Fe‐limiting experiments focusing on Nd and Fe accumulation as well as chelator secretion.

### 2.2 Influence of Iron‐Limiting Conditions on Nd Cell Accumulation and MLL Excretion

To analyze the effect of Fe limitation on the Ln accumulation with the goal to investigate the potential relationship between the Ln and Fe metabolism, we turned to scICP‐MS. By using scICP‐MS instead of directly measuring acid‐digested cell pellets, we wanted to circumvent a potential interference of the precipitated MLL‐Ln complex among the cell pellet. In order to link the observations to MLL secretion, we quantified MLL using LC‐MS in the corresponding supernatants.

In line with the experiments performed in our recent publication,^[^ ^27^ ^]^ we performed the cultivation experiments with both WT and the Ln‐dependent Δ*mxaF* mutant of *M. extorquens* AM1 in ½ Hypho medium in the presence of the C_1_ carbon source methanol using NdCl_3_ as Ln source. Cells were harvested in the early stationary phase, and the supernatant was filtered, desalted, and analyzed by high‐resolution LC‐MS. The obtained cells were washed and fixed using glutaraldehyde prior to investigation via scICP‐MS with regard to Fe and Nd levels. It should be noted that even though the cells have been extensively washed in the fixation workflow, we cannot differentiate between metals strongly associated with the cells and metals accumulated by the cells. However, scICP‐MS has a major advantage over the analysis of centrifuged cell pellets after nitric acid digestion, as less comeasurement of potentially precipitated metals (and complexes) should appear and only strongly adsorbed and accumulated metals should be detected. Along with the cell analyses, we also performed a control measurement of MLL mixed with NdCl_3_, showing that even if a potentially precipitated MLL‐Nd complex would still be present after the washing step, it would not interfere with the analysis.

By scICP‐MS, we found for both, the WT as well as the mutant, a significant higher Nd content for cells grown under Fe‐limiting conditions in comparison to cells grown in Fe‐replete medium (**Figure** **4**A). For both strains, no significant differences between the Fe levels in respect to Fe‐limiting conditions could be observed (exemplary shown for WT in Figure S20, Supporting Information), underlining the tightly regulated Fe household of bacteria.^[^ ^38^ ^]^ This is in line with what was previously observed by Good et al. for *M. extorquens* AM1 cultivations using magnet swarf.^[^ ^39^ ^]^

When analyzing the obtained supernatants of the associated samples, a 1.2‐fold for the WT and 10‐fold higher MLL concentration for Δ*mxaF* mutant supernatant samples can be found under Fe limitation (Figure 4B). These results are in line with the observations made previously, also showing a higher activity of the *mll* promoter under Fe‐limiting conditions for both WT and Δ*mxaF* mutant in the presence of MeOH and NdCl_3_. Furthermore, we also observe a significant difference between the WT and Δ*mxaF* mutant under Fe‐replete conditions, again fitting the previously published promoter fusion experiments which found a higher activity for the WT than the Ln‐dependent Δ*mxaF* mutant.^[^ ^27^ ^]^ This is further evidence for an involvement in the “Ln‐switch” as we have previously suggested in Zytnick et al.^[^ ^27^ ^]^ and in line with other observations made in the literature^[^ ^21^ ^]^ that suggested the involvement of metallophores in the “Ln‐switch” mechanism. An overproduction of a chelator under Fe limitation is commonly connected to it being a siderophore, thus suggesting that MLL is also involved as siderophore. As RPB B shows a similar binding behavior, one could rashly conclude that MLL might only be a siderophore which gets mismetallated by Lns and precipitates. This would therefore withdraw the chelator for Fe^3+^ uptake and thus indirectly inducing Fe limitation which in turn induces an overproduction of the siderophore to maintain normal Fe levels. Juma et al. recently showed that in *Methylobacterium aquaticum* 22A, a higher promoter (P_*sbnA*_) activity for the siderophore cluster *sbn* can be observed at high LaCl_3_ concentrations (90 µM) with low Fe versus high Fe levels, from which they concluded that LaCl_3_ might create Fe scarcity.^[^ ^21^ ^]^ They also suggested that the excreted siderophores are involved in the Ln uptake, especially under limitation of citric acid which they also showed to be potentially involved in the Ln uptake machinery.^[^ ^21^ ^]^ In addition, it has been shown for *Pseudomonas putida* KT2440 that REE‐dependent growth is strongly influenced by the presented Fe concentration.^[^ ^40^ ^]^ One should note that the Ln uptake machineries may differ from the herein discussed *M.* extorquens AM1. Taken together, our findings stress the connection between the Ln and Fe metabolism in Ln‐using bacteria. The role of chelators in Ln uptake systems seems to be even more complex than originally anticipated. To follow the potential involvement of MLL as siderophore, we further explored the Fe^3+^‐binding capabilities of MLL in comparison to the siderophore RPB B.

### 2.3 Fe^3+^ Binding of MLL and RPB B in Competition with Chromeazurol S

**Figure 5:**
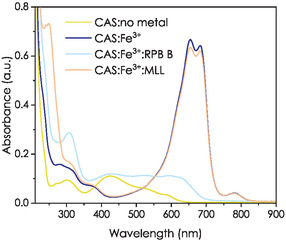
CAS competition experiment of RPB B and MLL with FeCl_3_ in buffer (10 mM MOPSO, 100 mM KCl, pH 6.0; final concentration: CAS/HDTMA (18.75 µM/200 µM), FeCl_3_ (18.75 µM), and ligand (18.75 µM)).

We performed a competition experiment using the chromophore chromeazurol S (CAS) based on the assay by Schwyn and Neilands.^[^ ^41^ ^]^ We used a mixture of CAS, the cationic surfactant hexadecyltrimethylammonium bromide (HDTMA), and ferric iron, to which we added the respective ligand after a 1 h incubation in the dark at r.t. After further incubation (2 h) in the dark, UV–vis spectra were recorded. From **Figure** **5**, it can readily be seen that RPB B (as expected) can efficiently “steal” Fe^3+^ from the CAS/HDTMA‐Fe^3+^ complex and has therefore a higher affinity for Fe^3+^ than the chromophore CAS. For MLL, only a slight reduction of the absorbance associated with the CAS/HDTMA‐Fe^3+^ complex can be observed, suggesting that MLL can bind Fe^3+^, but only with a low affinity. This fits the expectation that phenolic groups in para‐positions are less competent ligands for Fe^3+^ than the bidentate 3,4‐dihydroxy moiety of RPB B and that MLL is not a typical siderophore. To further investigate the Fe^3+^ complexation, we performed NMR titration experiments with trivalent gallium as diamagnetic proxy for ferric iron (Figure S10 and S12, Supporting Information).^[^ ^42^ ^,^ ^43^ ^]^ For MLL, no steady decrease of all signals can be observed, contradicting a precipitation of MLL with Ga^3+^. Slight upfield shifts can be observed for the aromatic signals (6.94 and 7.67 ppm) of the para‐substituted phenol. The NH (7.77 ppm) and CH_2_ (2.56, 2.66 ppm) signals of the citrate core decrease, get broader, and vanish at a 1:3 ligand to metal concentration but show no significant shift. Next to the signal of the acetyl group (2.08 ppm), a doublet appears from ligand to metal ratios of 1:0.5 to 1:2 and vanishes at 1:3. To summarize, the titration experiment with Ga^3+^ and MLL suggests the binding of Ga^3+^ and shows no indication of complex precipitation, therefore suggesting that MLL could in principle form soluble complexes with ferric iron. For RPB B, the addition of 0.5 equiv. Ga^3+^ already induces major spectral changes involving signal broadening, shifts, and the emerging of new signals showing the formation of a RPB B‐Ga^3+^ complex. This fits the results from the CAS competition experiment: RPB B is capable of binding Fe^3+^ with high affinity, while MLL seems to bind Fe^3+^, but with much lower affinity. Furthermore, siderophores are often detected in the micromolar range in bacterial supernatants,^[^ ^28^ ^]^ especially when cultivated under Fe limitation, while we find MLL concentrations below 100 nM under Fe limitation. One might argue that, if MLL precipitates with added (soluble) Lns, the detected supernatant levels could be lower as it might be removed concurrently with the cells from the supernatant upon centrifugation. However, this would imply that if it would indeed act as common siderophore, rather micromolar levels of MLL should be found in the supernatant when cultivating the WT in the absence of Lns under Fe limitation. However, via LC‐MS analysis, we showed that the detected MLL concentration still remains in a similar range at nanomolar concentrations (Figure S21, Supporting Information).

As CAS can form complexes not only with Fe^3+^ but also with Lns,^[^ ^44^ ^,^ ^45^ ^]^ we conducted a similar experiment with Nd^3+^. For both chelators almost the same spectral changes can be observed, suggesting a similar binding behavior for Nd^3+^ (Figure S8, Supporting Information). As only a small reduction of the bands associated with the CAS/ HDTMA‐Nd^3+^ complex could be observed, a lower affinity of MLL and RPB B to Nd^3+^ in comparison to CAS can be assumed. It should be noted that we cannot tell from this experiment whether the formed complex precipitates or stays in solution as both scenarios would lead to a decrease of the absorption band associated with the CAS/HDTMA‐Nd^3+^ complex.

Both RPB B and MLL appear to have a similar binding affinity toward Nd^3+^, and RPB B exhibits a significantly higher affinity toward Fe^3+^ than MLL, distinguishing the latter from being a common siderophore. As we observe the precipitation of both chelators with Lns and thus suspect that this could lead to secondary effects such as chelator limitation followed by overproduction, the careful consideration of the used growth medium when investigating the impact of metal ions on bacteria becomes very important. For example, depending on the phosphate content, the metal availability in a used medium can drastically change. Therefore, we started to look into the true bioavailability of Ln ions in the used ½ Hypho medium. For this, we turned again to TRLFS using Eu^3+^ luminescence as reporter and proxy for other Lns. TRLFS has already been used successfully in the literature to investigate the speciation in biological media.^[^ ^46^ ^,^ ^47^ ^]^ We found that with EuCl_3_, the actual Eu^3+^ (thus Ln^3+^) concentration in solution is far from the added 2 µM Lns and not present as easily available Eu^3+^‐aquo ion, but rather a phosphate‐like species which precipitates partially as well (Figure S5 and S22, Supporting Information). We also tested the availability of Ln oxides using Eu_2_O_3_ as a proxy and were able to show that with this poorly soluble starting material, we cannot detect any soluble species after incubation in ½ Hypho medium (in the absence of bacteria and excreted ligands to mobilize Lns). These observations support the claim that the oxides are indeed less bioavailable in the beginning of a cultivation experiment and thus a good mimic for biological Ln uptake scenarios (Figure S22, Supporting Information). However, it also highlights that components such as phosphates in media significantly lower the amount of soluble Ln species far from the intentionally added concentrations.

### 2.4 Investigation of RPB B and MLL with REEs in the Gas Phase by Ion Mobility Measurements and Quantum Chemical Calculations

While we have not been able to determine the structure of a MLL‐Ln^3+^ complex by X‐ray crystallography so far, and the precipitating complexes (or complex polymers) may well be neutral complexes, we successfully observed doubly charged MLL‐Ln^3+^ complexes using mass spectrometry.^[^ ^27^ ^]^ As MS measurements were so far the only method where we could observe 1:1 complexes of MLL and Ln without precipitation, we decided to investigate these complexes with additional gas phase techniques to investigate differences in Fe and Ln coordination.

**Figure 6:**
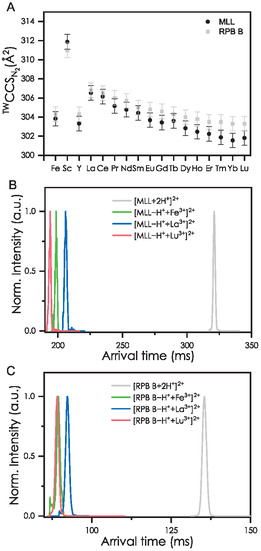
A) Experimental ^TW^CCS_N2_ of [MLL−H^+^+M^3+^]^2+^ and [RPB B−H^+^ +M^3+^]^2+^ (M = 3H, Fe, REEs, except Pm). ATDs of the ions [Ligand−H^+^ + M^3+^]^2+^ with M = 3H, La, Lu, Fe for B) MLL and C) RPB B.

Gas‐phase IMS is a technique that is sensitive to the shape of isolated ions and allows isomer separation in a mass spectrometer. In brief, a packet of mass‐selected ions is guided by an electrical field through a gas‐filled drift cell (typically 1–2 mbar of nitrogen). The arrival time distribution (ATD) is measured as a function of the applied field and pressure. Bi‐ or multimodal ATDs imply the presence of isomers. The ATD can be deconvoluted into ion mobility and finally into a collision cross section (CCS).^[^ ^48^ ^]^ These “experimental” CCSs can be compared with predictions from theory (here obtained by quantum chemical and trajectory calculations) to confirm or rule out possible candidate structures. By traveling wave (TW) IMS coupled to mass spectrometry we observed for MLL and RPB B, the following complex: [Ligand−H^+^+M^3+^]^2+^ (M = Fe, REEs, except Pm) which is in line with the complexes reported in the literature.^[^ ^27^, ^28^ ^]^ The arrival times for MLL and RPB B as doubly charged free ligands and complexed with La^3+^, Lu^3+^, and Fe^3+^ are shown in **Figure** **6**B,C. For both, [MLL + 2H^+^]^2+^ and [RPB B + 2H^+^]^2+^, later arrival times as well as larger CCS than those of the metallated species (at the same charge state) are observed. This is expected as the coordination to a metal ion forces the molecule to form a less flexible and more rigid structure.

Both [MLL−H^+^+M^3+^]^2+^ and [RPB B−H^+^+M^3+^]^2+^ (M = Fe, REEs, except Pm) complexes show the same trend (Figure 6A). The CCSs of the RPB B metal complexes are in most cases slightly larger (<0.5%) than the MLL metal complexes which can be explained by the catechol‐binding moiety instead of the phenol moiety. As only small variations between the CCS of all tested metal complexes are observed, we conclude that MLL and RPB B form similar 3D structures with the respective metal. Interestingly, the scandium complexes [MLL−H^+^+Sc^3+^]^2+^ and [RPB B−H^+^+Sc^3+^]^2+^ have significantly larger CCS (311.9 and 311.0 Å^2^, respectively), even though the ion radius of Sc^3+^ (0.745 Å) is much smaller than the ion radii of the lanthanides (1.032–0.861 Å).^[^ ^49^ ^]^ Only [MLL + 2H^+^]^2+^ and [RPB B + 2H^+^]^2+^ still have larger CCS values with 355.0 and 353.1 Å^2^, respectively (Table S5, Supporting Information). We tentatively attribute this to the fact that for Sc^3+^, more open structures, like the third structure shown in Figure S26, Supporting Information (+0.23 eV, 346 Å^2^), are energetically close to the calculated minimum structure and therefore probably contribute to the observed CCS, possibly in a dynamic equilibrium (compare Figure S29, Supporting Information, for calculations of [RPB B−H^+^+Sc^3+^]^2+^). In the case of La^3+^, this structure (Figure S24, Supporting Information, structure 4) is energetically out of reach (+1.07 eV).

The lowest energy protomer of [MLL + 2H^+^]^2+^ with a quantum theoretically calculated CCS (^theo^CCS) of 364 Å^2^ is shown in Figure S23, Supporting Information, and is in good agreement with the experimental value (ca. 3% larger; 2%–5% deviation between experiment and calculation are common).^[^ ^50^ ^]^ For [MLL−H^+^+La^3+^]^2+^, geometry optimizations yielded several local minima which are shown in Figure S24, Supporting Information. In all cases, the La^3+^ center is surrounded by six to seven oxygen atoms with O—La distances between 2.4 and 2.6 Å. The lowest energy structure has a ^theo^CCS of 324 Å^2^ (experimental: 306.5 Å^2^). Based on the calculated CCS, we cannot differentiate between the first three structures shown in Figure S24, Supporting Information, as they show very similar coordination patterns with La^3+^ being mono‐ or bidentately bound by the carboxyl group, one amide oxygen of the citrate center of the molecule, and the acetyl oxygens and amide oxygens of the attached phenol groups (Figure S24, Supporting Information). However, two of the five structures (structures 4 and 5 with +1.07 eV and +2.04 eV, respectively) can be ruled out based on their much larger ^theo^CCS. Thus, the calculations suggest that in the gas phase, the phenol group may not be involved in metal coordination. By replacing La^3+^ with Lu^3+^ and reoptimizing the geometries, five local minima were obtained again (Figure S25, Supporting Information) which all show a decrease of the ^theo^CCS when compared with La^3+^, thus reproducing the experimentally observed reduction in CCS from La^3+^ to Lu^3+^. For [RPB B+2H^+^]^2+^ and [RPB B−H^+^+La^3+^]^2+^, the obtained results are similar (Figure S27 and S28, Supporting Information). Interestingly, the calculations indicate for La^3+^ binding in the gas phase that there is no involvement of the catechol moiety, thus producing very similar structures to [MLL−H^+^+La^3+^]^2+^.

## 3 Conclusion

MLL and RPB B were successfully synthesized, and for both metallophores, a comparable Ln‐binding behavior was observed. Surprisingly, both seem to form poorly soluble complexes at biologically relevant pH. For the MLL and Eu^3+^ system, we were able to determine the solubility product of the precipitated complex to be −12.07 ± 0.24 mol^2 ^L^−2^. By employing scICP‐MS in combination with LC‐MS analysis of the corresponding bacterial supernatants, we were able to show that under Fe limitation, more Nd gets accumulated, while Fe accumulation is not affected and higher concentrations of MLL can be found in the bacterial supernatant. The observed effects are significantly more pronounced for the Ln‐dependent Δ*mxaF* mutant than the WT strain, pointing toward a potential role in the “Ln‐switch.” Despite the fact that the MLL production is influenced by the Fe availability, it only shows low affinity toward Fe^3+^, which is produced in nanomolar concentrations, and the observed effects cannot be explained by simple correlations. Future studies on Ln uptake and metabolism should consider the role of Fe and closely investigate potential secondary effects due to unintentionally induced metal scarcity or availability. Growth media are usually optimized to support optimal growth of bacteria but do not do necessarily reflect biological conditions found in nature. Further, the true metal ion concentrations and their availability in solution may differ from the originally added amount due to formation of insoluble complexes or salts, further complicating analyses.

## Conflict of Interest

The authors declare no conflict of interest.

## Author Contributions

**Sophie M. Gutenthaler‐Tietze** and **Lena J. Daumann** designed the study and wrote the initial draft of the manuscript. **Sophie M. Gutenthaler‐Tietze** cultivated bacteria, isolated MLL, performed MLL quantifications and metal‐binding experiments, and analyzed the data. **Michael Mertens** and **Manh Tri Phi** synthesized RPB B and MLL. **Patrick Weis** performed IMS measurements and calculations. **Alexander Köhrer** measured and analyzed scICP‐MS. **Björn Drobot** performed TRLFS experiments and chemical microscopy and analyzed the data. **N. Cecilia** **Martinez‐Gomez** provided the *M. extorquens* AM1 strains and support for growth experiments. **Lena J. Daumann**, **N. Cecilia Martinez‐Gomez**, **Björn Drobot**, **Uwe Karst**, and **Robin Steudtner** provided the necessary infrastructure for this work. All authors were involved in reviewing this manuscript.

## Supporting information

Supplementary Material

## Data Availability

Primary data concerning the synthesis described within this manuscript and more detailed reaction procedures are accessible at the Chemotion Online Repository under https://dx.doi.org/10.14272/collection/MWM_2024‐11‐19. UV–vis (.csv), TRLFS (.sif and processed), scICP‐MS event (.csv), NMR (*Bruker* raw data), and chemical microscopy data (raw data and processed) as well as quantum chemical calculations (.xyz) shown in this manuscript and its Supporting Information are available at the RODARE repository: https://doi.org/10.14278/rodare.3273.
