# Supplementary material for: Comparative Binding Studies of the Chelators Methylolanthanin and Rhodopetrobactin B to Lanthanides and Ferric Iron
Source: Chembiochem. 2025 Dec 1;27(1):e202500312. doi: 10.1002/cbic.202500312 (PMC12780827; doi:10.1002/cbic.202500312)
Supplement: Supplementary file 1 — Supplementary Material [file CBIC-27-e202500312-s001.pdf]

Supporting Information

Comparative Binding Studies of the Chelators Methylolanthanin and Rhodopetrobactin B to Lanthanides and Ferric Iron

Sophie M. Gutenthaler-Tietze[a,b], Michael Mertens[a,b], Manh Tri Phi[a], Patrick Weis[c], Björn Drobot[d], Alexander Köhrer[e], Robin Steudtner[d], Uwe Karst[e], N. Cecilia Martinez-Gomez[f], Lena J. Daumann*[b]*

*corresponding authors: lena.daumann@hhu.de, cecimartinez@berkeley.edu

[a] Department of Chemistry, Ludwig Maximilians University of Munich, Butenandtstraße 5-13, 81377 Munich (Germany)
[b] Chair of Bioinorganic Chemistry, Heinrich Heine University Düsseldorf, Universitätsstr. 1, 40225 Düsseldorf (Germany)
[c] Institute of Physical Chemistry, Karlsruhe Institute of Technology, Fritz-Haber-Weg 2, 76131 Karlsruhe (Germany)
[d] Institute of Resource Ecology Helmholtz-Zentrum Dresden-Rossendorf e.V. Bautzner Landstraße 400, 01328 Dresden (Germany)
[e] Institute of Inorganic and Analytical Chemistry, University of Münster, Corrensstraße 48, 48149 Münster (Germany)
[f] Department of Plant and Microbial Biology, University of California–Berkeley, California 94720 (United States)

Table of Contents

1 Organic Synthesis 3

1.1 General Working Methods 3

1.2 Synthesis of Rhodopetrobactin B (13a) and Methylolanthanin (13b) 5

1.3 NMR Spectra 21

2 Binding Studies 35

2.1 General working methods 35

2.2 UV-vis Spectroscopy 36

2.3 Metal to Ligand NMR Titration Experiments 37

2.4 Time-resolved laser-induced fluorescence spectroscopy (TRLFS) 37

2.5 Chemical Microscopy 44

2.6 Cyclic Ion Mobility Spectrometry-Mass Spectrometry (cIMS-MS) 45

3 Investigation of Nd Cell Accumulation and MLL Excretion 46

3.1 General Working Methods 46

3.2 Cultivation in 48-well plates 46

3.3 Cell Fixation with Glutaraldehyde 47

3.4 Sample Preparation and LC-MS analysis 47

3.5 scICP-MS 48

4 Quantum Chemical Calculations 48

5 Supplementary Tables and Figures 50

6 Data Availability 69

7 References 69

1 Organic Synthesis

1.1 General Working Methods

Solvents and Chemical Reagents

Reagents, dry solvents and deuterated organic solvents were purchased from commercial suppliers (*ThermoFischer, TCI, Sigma Aldrich, Apollo Scientific, BLDpharm, Eurisotop*) and used without further purification.

All reactions were performed under inert nitrogen atmosphere unless stated otherwise. Chemicals sensitive to air and/or water were handled using dried solvents and Schlenk techniques. Reactions were carried out in one-neck round-bottomed flasks with septum, which were evacuated and filled with N_2 *via* cannulas. Silica-coated aluminium plates (0.25 mm, 60 Å pore-size, *Merck KGaA*) were used for thin layer chromatography (TLC). Compound spots were detected using a UV light at λ = 254 nm and 365 nm, if not stated otherwise. TLC plates were bought as 20 × 20 cm sheets and cut by hand. TLC was performed on plates with a length (direction of flow) of 6 cm, substances were applied at a distance of 1 cm to the bottom and the chromatography was stopped when the solvent front was approximately 0.5-1 cm from the top edge. Resulting in a travel distance of approx. 4-4.5 cm for the mobile phase. Flash column chromatography was performed with an *Interchim* puriFlash XS 520+ system using self-packed silica columns (Silica 60, 0.04 – 0.062 mm from *Macherey-Nagel*) for separation. The eluent ratios for TLC and column chromatography are given in percentages by volume. The eluent AM is a 1:9 mixture of an aqueous solution of ammonia (25%) in methanol. Solvents were removed under reduced pressure at 40 °C unless stated otherwise. Preparative reversed-phased HPLC separations were performed on an *Agilent* 1260 Infinity II (G1364E 1260 FC-PS, G7165A 1260 MWD, G7161A Prep Bin Pump) system. The used solvents were HPLC grade.

NMR spectroscopy

^{1}H and ^{13}C NMR spectra were either recorded at the Center for Molecular and Structural Analytics (CeMSA) of the HHU Düsseldorf using either a *Bruker* Avance III – 300 system operating at 300 MHz for ^{1}H and 75 MHz for $^{13}C\{^{1}H\}$ spectra or a *Bruker* Avance III – 600 system operating at 600 MHz for ^{1}H and 151 MHz for $^{13}C\{^{1}H\}$ spectra or at the Faculty of Chemistry and Pharmacy of the LMU Munich using either a *Bruker* Avance III – 400 system operating at 400 MHz for ^{1}H and 101 MHz for $^{13}C\{^{1}H\}$ spectra or a *Bruker* Avance III HD spectrometer equipped with a triple channel cryogenic probe operating at 800 MHz for ^{1}H and 201 MHz for $^{13}C\{^{1}H\}$ spectra. ^{1}H and ^{13}C chemical shifts are reported in ppm units relative to $CDCl_3$ (δ_H = 7.26 ppm, δ_C = 77.16 ppm (central line of the triplet), DMSO-d_6 (δ_H = 2.50 ppm,

δ_C = 39.52 ppm (central line of the septet), MeOD-d_4 (δ_H = 3.31 ppm, δ_C = 49.00 ppm or TMSP-d_4 (δ_H = 0 ppm, δ_C = 0 ppm). Coupling constants (*J*) are given in Hz. The assignment of all signals was done by using two-dimensional NMR spectroscopy experiments (^{1}H-^{1}H-COSY, ^{1}H-^{13}C-HSQC and ^{1}H-^{13}C-HMBC).

Mass spectrometry

Electronspray ionization (ESI)-High resolution mass spectrometry was either performed on a *Bruker* Daltonics UHR-QTOF maXis 4G system in positive ion mode at the CeMSA of the HHU Düsseldorf or on an *Agilent* QTOF 6530 C coupled to an *Agilent* HPLC 1260 Infinity II instrument (G7115A 1260 DAD WR, G7116A 1260 MCI, G7167A 1260 multisampler, G7104C 1260 flexible pump) equipped with an *Agilent* Poroshell 120 EC-C18 column (3.0 × 150 mm, 2.7 µm) in positive ion mode.

1.2 Synthesis of Rhodopetrobactin B (13a) and Methylolanthanin (13b)

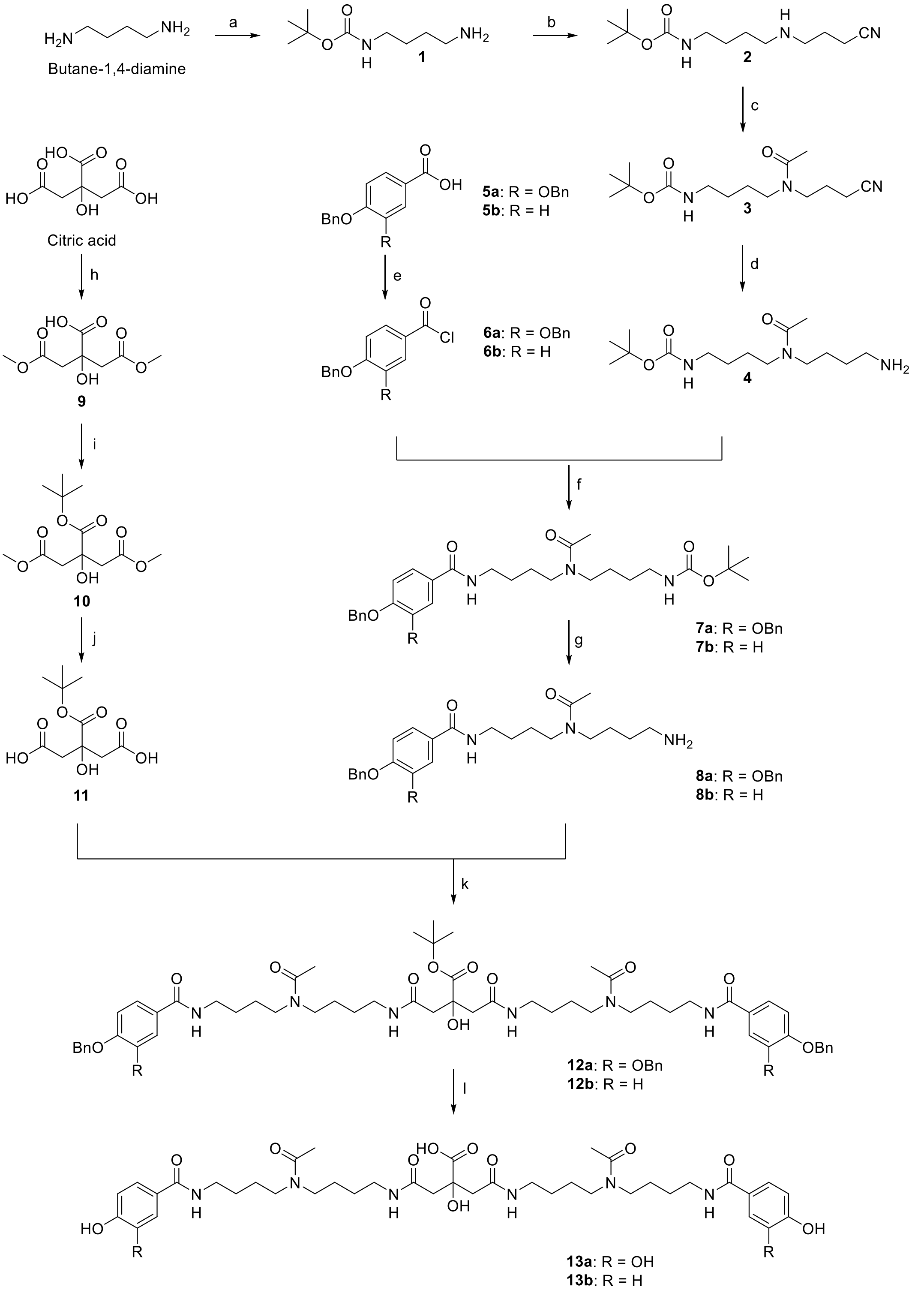

Scheme S1 Reagents and conditions: **(a)** Di-*tert*-butyl dicarbonate (0.33 equiv.), TEA, MeOH, 20 °C, 18 h; **(b)** 4-Bromobutyronitrile (1.50 equiv.), K_2CO_3, MeCN, 60 °C, 3 h; **(c)** Acetyl chloride (1.04 equiv.), TEA, DCM, 23 °C, 18 h; **(d)** $NaBH_4$ (10.0 equiv.), $CoCl_2 \cdot 6\,H_2O$ (2.00 equiv.), THF, 22 °C, 3 h; **(e)** for **6a:** Oxalyl chloride (8.37 equiv.), DMF, DCM, 0 °C for 30 min then 22 °C for 2; for **6b:** Oxalyl chloride (2.65 equiv.), DMF, DCM/Toluene (5:2), 22 °C, 2 h; **(f)** DCM, TEA, 22 °C, 2 h; **(g)** DCM, TFA, 0 °C for 1 h then 20 °C for 2 h; **(h)** NaI (0.3 equiv.), MeOH, Dowex-H^+ resin, 20-22 °C, 28 h; **(i)** 1) *tert*-butyl acetate, perchloric acid, 20-22 °C, 3 d; **(j)** 2 M NaOH, MeOH, 0 °C for 30 min then 24 h at 20-22 °C; **(k)** 1) **11**, DCC (3.00 equiv.), NHS (3.00 equiv.), DCM, THF, 22 °C, 3 h, 2) **8a/b**, TEA, 1,4-Dioxane/DCM (1:1), 10 °C for 30 min then 23 °C for 16 h (**12a**) or 66 h (**12b**); **(l)** for **13a:** 1) AcOH/HCl (5:2), 22 °C, 2 h, 2) Pd/C (10 wt.%), H_2 (1 atm), EtOH, 20 °C, 2 h; **13b:** AcOH/HCl (1:1), 22 °C, 3 d.

Compound 1

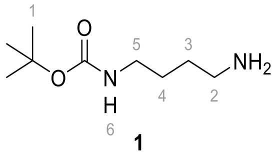

1

The reaction was performed according to a modified literature procedure.[1]

In a 250 mL round-bottomed flask under standard atmospheric conditions, 1,4-diaminobutane (6.20 g, 70.1 mmol, 1.00 equiv.) was dissolved in a solution of 135 mL methanol (MeOH) and 15 mL triethylamine (TEA). Di-*tert*-butyl dicarbonate (5.10 g, 23.4 mmol, 0.33 equiv.) was dissolved in 15 mL MeOH and rapidly added to the solution using a glass pipette. The reaction mixture was stirred for 18 h at 20 °C. After completion, the volatiles were removed under reduced pressure. The residue was dissolved in 150 mL dichloromethane (DCM) and washed four times with 150 mL saturated Na_2CO_3 solution and the combined organic layers were dried over Na_2SO_4. The volatiles were removed under reduced pressure and purified by flash column chromatography (DCM/MeOH, 0 to 10% + 1% TEA). The product was obtained as a colourless oil (3.10 g, 16.5 mmol, 70%).

^{1}H NMR (400 MHz, $CDCl_3$) δ/ppm = 4.70 (s, 1H), 3.10 (q, J = 6.5 Hz, 2H), 2.68 (t, J = 6.6 Hz, 2H), 1.54 – 1.43 (m, 4H), 1.41 (s, 9H), 1.22 (s, 2H).

^{13}C NMR (101 MHz, $CDCl_3$) δ/ppm = 156.1, 79.1, 79.1, 41.9, 40.5, 31.0, 28.5, 27.6.

HR-MS (ESI): m/z calcd for $C_9H_{21}N_2O_2^+$: 189.1598 $[M+H]^+$, found: 189.1599 (+0.529 ppm).

Compound 2

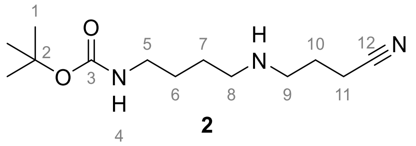

2

The reaction was performed according to a modified literature procedure.[1]

In a 250 mL round-bottomed flask, compound **2** (2.20 g, 11.7 mmol, 1.00 equiv.) was dissolved in 100 mL of dry acetonitrile. Potassium carbonate (3.23 g, 23.4 mmol, 2.00 equiv.) was dried under reduced pressure for 1 h in another flask before being immediately added to the *tert*-butyl(4-aminobutyl)carbamate at once. Then, 4-bromobutyronitrile (1.75 mL, 17.6 mmol, 1.50 equiv.) was added with a syringe and the reaction mixture heated to 60 °C for 3 h. The solids were filtered off and the volatiles were removed under reduced pressure. The crude product was purified by flash column chromatography (silica, gradient from 5 % to 10 % AM in DCM) yielding compound **2** as clear oil (379 mg, 1.48 mmol, 13%).

R_f (DCM/AM 95:5; ninhydrin) = 0.18.

^{1}H NMR (600 MHz, $CDCl_3$) *δ*/ppm = 4.78 (s, 1H, N-4), 3.12 (q, *J* = 6.4 Hz, 2H, 5-H), 2.73 (t, *J* = 6.7 Hz, 2H, 9-H), 2.61 (t, *J* = 6.6 Hz, 2H, 8-H), 2.44 (t, *J* = 7.1 Hz, 2H, 11-H), 1.80 (p, *J* = 6.9 Hz, 2H, 10-H), 1.57 – 1.46 (m, 4H, 6/7-H), 1.43 (s, 9H, 1-H).

^{13}C NMR (151 MHz, $CDCl_3$) *δ*/ppm = 156.1 (C-3), 119.8 (C-12), 79.1 (C-2), 49.4 (C-8), 48.1 (C-9), 40.5 (C-5), 28.5 (C-1), 27.9 (C-6/7), 27.5 (C-6/7), 25.9 (C-10), 15.1 (C-11).

HR-MS (ESI): *m/z* calcd for $C_{13}H_{26}N_3O_2^+$: 256.2020 [*M*+H]$^+$; found: 256.2019 (−0.390 ppm).

Compound 3

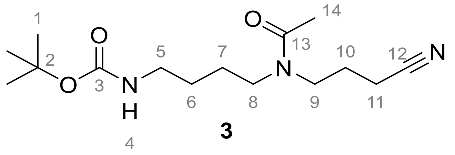

3

In a 50 mL round-bottomed flask compound **2** (103 mg, 0.403 mmol, 1.00 equiv.) was dissolved in 20 mL dry DCM and TEA (0.10 mL, 0.718 mmol, 1.78 equiv.). The solution was cooled to 0 °C before acetyl chloride (0.03 mL, 0.420 mmol, 1.04 equiv.) was added dropwise with a syringe. The cooling was removed and the reaction mixture stirred for 18 h at 23 °C. Afterwards 20 mL of water were added and the reaction mixture was stirred for another 30 min. The layers were separated and the aqueous phase extracted with DCM (3 × 30 mL). The combined organic phases were washed once with a saturated solution of $NaHCO_3$ and NaCl, then dried over Na_2SO_4. Volatiles were removed under reduced pressure and the crude product purified by flash column chromatography (silica, 5 % AM in DCM) yielding compound **3** as a clear oil (101 mg, 0.340 mmol, 84%).

Rf (DCM/AM 95:5; ninhydrin) = 0.25.

^{1}H NMR (600 MHz, $CDCl_3$) δ/ppm = 4.61 (d, J = 33.2 Hz, 1H, NH-4), 3.41 (dt, J = 11.8, 7.3 Hz, 2H, 9-H), 3.34 – 3.25 (m, 2H, 8-H), 3.14 (dq, J = 14.2, 6.7 Hz, 2H, 5-H), 2.39 (dt, J = 18.4, 7.0 Hz, 2H, 11-H), 2.10 (d, J = 15.1 Hz, 3H, 14-H), 1.96 – 1.87 (m, 2H, 10-H), 1.63 – 1.54 (m, 2H, 7-H), 1.53 – 1.46 (m, 2H, 6-H), 1.43 (d, J = 3.6 Hz, 9H, 1-H).

^{13}C NMR (151 MHz, $CDCl_3$) δ/ppm = 171.0 – 170.3 (C-13), 156.1 (C-3), 119.5 (C-12), 79.5 (C2), 48.9 (C-8), 47.1 (C-9), 45.3 (C-8), 44.6 (C-9), 39.9 (C-5), 28.5 (C-1), 27.7 (C-6), 26.1 (C-7), 24.9 (C-7), 23.9 (C-10), 21.6 (C-14), 15.1 – 14.7 (C-11).

HR-MS (ESI): *m/z* calcd for $C_{15}H_{28}N_3O_3^+$: 298.2125 [M+H]$^+$; found: 298.2120 (−1.67 ppm).

Compound 4

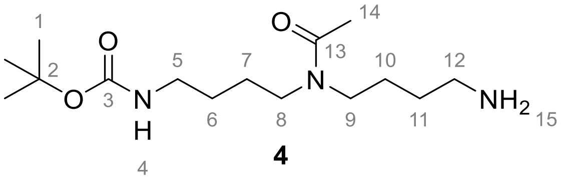

4

In a 100 mL round-bottomed flask compound **3** (184 mg, 0.620 mmol, 1.00 equiv.) was dissolved in 50 mL dry tetrahydrofuran (THF) before $CoCl_2 \cdot 6\ H_2O$ (295 mg, 1.24 mmol, 2.00 equiv.) was added at once, resulting in a deep blue solution. Subsequently, $NaBH_4$ (235 mg, 6.20 mmol, 10.0 equiv.) was added in three equal portions, resulting in the formation of a black solution. The mixture was stirred for 3 h at 22 °C, then 20 mL of water were added. The solids were filtered off and the layers were separated. The aqueous phase was extracted with DCM (3 × 50 mL), the combined organic phases washed with a saturated NaCl solution and dried over Na_2SO_4. Volatiles were removed under reduced pressure and the crude product purified by flash column chromatography (silica, gradient from 5% to 10% AM in DCM) yielding compound **4** as yellowish oil (97.1 mg, 0.322 mmol, 52 %).

R_f (DCM/AM 9:1; ninhydrin) = 0.10.

^{1}H NMR (600 MHz, $CDCl_3$) δ/ppm = 4.69 (d, J = 35.9 Hz, 1H; N-H(4)), 3.32 – 3.26 (m, 2H, 8-H), 3.22 (q, J = 7.7 Hz, 2H, 9-H), 3.12 (dq, J = 12.9, 6.7 Hz, 2H, 5-H), 2.70 (dt, J = 13.5, 7.0 Hz, 2H, 12-H), 2.05 (d, J = 4.0 Hz, 3H, 14-H), 1.62 – 1.47 (dd, J = 9.1, 5.7 Hz, 8H, 6/7/10/11-H), 1.43 (s, 9H, 1-H).

^{13}C NMR (151 MHz, $CDCl_3$) δ/ppm = 170.2 (C-13), 156.1 (C-3), 79.4 – 79.1 (C-2), 48.8 – 48.5 (C-14), 45.5 – 45.3 (C-12), 41.9 (C-8), 40.2 – 40.0 (C-5), 30.9 (C-6/7), 28.5 (C-1), 27.6 – 27.5 (C-6/7), 26.5 – 26.1 (C-10/11), 25.1 – 25.0 (C-10/11), 21.6 (C-14).

HR-MS (ESI): *m/z* calcd for $C_{15}H_{32}N_3O_3^+$: 302.2438 [M+H]$^+$; found: 302.2438 (0.000 ppm).

Compound 5a

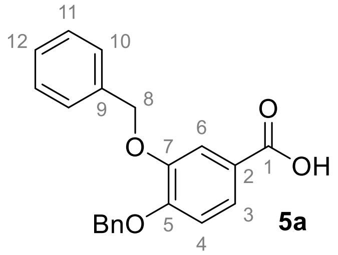

5a

The synthesis was performed according to a literature procedure.[1]

In a 50 mL round-bottomed flask under standard atmospheric conditions 3,4-dihydroxybenzoic acid (1.00 g, 6.50 mmol, 1.00 equiv.) was dissolved in 25 mL acetone and K_2CO_3 (2.50 g, 18.1 mmol, 2.79 equiv.) was added at once. Then, benzyl bromide (4.60 mL, 38.7 mmol, 6.00 equiv.) was added with a syringe and the reaction was heated to 70 °C for 21 h. After completion, the solids were filtered off and the volatiles were removed under reduced pressure. The residue was redissolved in a solution of 25 mL MeOH and 12.5 mL 6 M NaOH. The reaction mixture was heated to 80 °C for 2 h first and then stirred for 16 h at 22 °C. After completion, volatiles were removed under reduced pressure, and the residue was re-dissolved in 150 mL of water. The aqueous solution was washed with *iso*-hexane (3 × 50 mL). The aqueous layer was then acidified with 3 M HCl and stored at 5 °C for 3 d. The resulting white solid was filtered off and dried in a vacuum oven (60 °C, 1 mbar, 18 h). Compound **5a** was obtained as a colourless solid (1.02 g, 3.05 mmol, 47%).

^{1}H NMR (600 MHz, DMSO-*d*$_6$) δ/ppm = 12.29 (s, 1H), 7.56 – 7.52 (m, 2H), 7.42 – 7.38 (m, 4H), 7.35 – 7.28 (m, 4H), 7.28 – 7.22 (m, 2H), 6.98 (d, *J* = 8.6 Hz, 1H), 5.12 (d, *J* = 26.2 Hz, 4H).

^{13}C NMR (151 MHz, DMSO-*d*$_6$) δ/ppm = 166.9, 151.9, 147.4, 136.4, 136.1, 128.0, 127.9, 127.5, 127.4, 127.0, 126.9, 123.4, 123.3, 114.8, 112.6, 70.2, 69.9.

HR-MS (ESI): *m/z* calcd for $C_{21}H_{19}O_4^+$: 335.1278 $[M+H]^+$; found: 335.1280 (+0.597 ppm).

Compound 6a

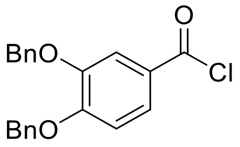

6a

In a 10 mL round-bottomed flask compound **5a** (46.6 mg, 0.139 mmol, 1.00 equiv.) was dissolved in 5 mL dry DCM and cooled to 0 °C. Three drops of *N*,*N*-dimethylformamide (DMF) were added before oxalyl chloride (0.10 mL, 1.17 mmol, 8.37 equiv.) was added dropwise with a syringe. After some drops of the reagent, the prior turbid solution turned clear. The cooling was removed and the solution was stirred for 2 h at 22 °C. After completion volatiles were removed under reduced pressure and the resulting yellow solid was used without further purification in the synthesis of compound **7a**.

Compound 7a

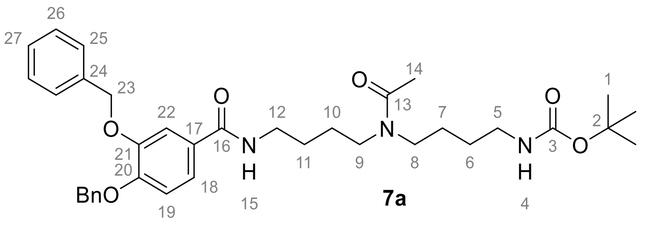

7a

In a 10 mL round-bottomed flask compound **4** (35.0 mg, 0.116 mmol, 1.00 equiv.) was dissolved in 5 mL dry DCM and TEA (0.200 mL, 1.44 mmol, 12.4 equiv.). Then, the solution was cooled to 0 °C. Compound **6a** (49.1 mg, 0.139 mmol, 1.20 equiv.) was dissolved in 5 mL dry DCM and transferred with a syringe dropwise to compound **4**. Then, the cooling was removed and the solution was stirred for 20 h at 22 °C. After completion, volatiles were removed under reduced pressure and the crude product was purified by flash column chromatography (silica, gradient from 2% to 5% AM in DCM) yielding compound **7a** as yellowish oil (16.0 mg, 25.8 µmol, 22%).

R_f (DCM/AM 95:5) = 0.10.

^{1}H NMR (600 MHz, $CDCl_3$) δ/ppm = 7.59/7.50 (m, 1H, 18/22-H), 7.48 – 7.41 (m, 4H, 25-H), 7.39/7.25 (m, 1H, 18/22-H), 7.36 (ddt, J = 7.9, 6.2, 2.3 Hz, 4H, 26-H), 7.32 – 7.28 (m, 2H, 27-H), 6.94 (m, 1H, 15-H), 6.91 (m, 1H, 19-H), 5.19 (dd, J = 8.3, 3.2 Hz, 4H, 23-H), 4.66 (d, J = 41.6 Hz, 1H, 4-H), 3.46 (dq, J = 23.6, 6.2 Hz, 2H, 12-H), 3.32 (dt, J = 28.0, 7.2 Hz, 2H, 8-H), 3.25 (q, J = 8.1 Hz, 2H, 9-H), 3.12 (dq, J = 17.6, 6.8 Hz, 2H, 5-H), 2.06 (d, J = 9.7 Hz, 3H,

14-H), 1.64 – 1.56 (m, 6H, 7/10/11-H), 1.50 (dq, J = 26.9, 7.7 Hz, 2H, 6-H), 1.43 (d, J = 4.3 Hz, 9H, 1-H).

^{13}C NMR (151 MHz, $CDCl_3$) δ/ppm = 170.6 – 170.3 (C-13), 167.1 – 167.0 (C-16), 156.2 – 156.1 (C-3), 151.7 – 151.5 (C-20), 148.9 – 148.7 (C-21), 137.1 – 136.7 (C-24), 128.7 – 128.6 (C-26), 128.1 – 128.0 (C-27), 127.9 – 127.7 (C-17), 127.2 (C-25), 120.5 – 120.1 (C-18/22), 114.1 – 113.9 (C-18/22), 113.8 (C-19), 79.5 (C-2), 71.4 – 71.0 (C-23), 48.6 – 48.5 (C-9), 45.2 – 45.0 (C-8), 39.9 (C-5), 39.4 (C-12) , 28.5 (C-1), 27.7 – 27.5 (C-6), 27.25 – 24.9 (C-7/10/11), 21.7 – 21.6 (C-14).

HR-MS (ESI): *m/z* calcd for $C_{36}H_{48}N_3O_6^+$: 618.3538 [M+H]$^+$; found: 618.3536 (−0.323 ppm).

Compound 8a

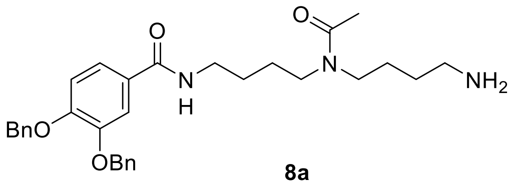

8a

In a 25 mL round-bottomed flask under standard atmospheric conditions compound **7a** (210 mg, 0.34 mmol) was dissolved in 5 mL DCM and stirred for 15 min at 0 °C. Then, 1 mL trifluoroacetic acid (TFA) was dissolved in 5 mL DCM and added dropwise with a syringe. The cooling was removed and the reaction mixture was stirred for 2 h at 20 °C. After completion, the volatiles were removed under reduced pressure and the crude compound **8a** was used immediately without further purification.

Compound 9

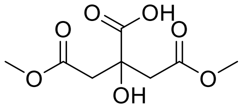

9

The synthesis was performed according to a modified literature procedure.[2]

Citric acid (4.8 g, 25 mmol, 1.0 equiv.) and sodium iodide (1.1 g, 7.5 mmol, 0.3 equiv.) were dissolved in 75 mL MeOH. 10 g dried Dowex-H^+ resin* was added and the reaction mixture was stirred for 28 h at room temperature. After completion, the resin was filtered off and the volatiles were removed under reduced pressure. The residue was dissolved in 100 mL DCM and the reaction mixture was stirred overnight at room temperature. The product forms as a white precipitate that was collected by filtration. After addition of 100 mL DCM to the filtrate, the reaction mixture was stirred for 4 h at room temperature. The product was collected again and the process was repeated once more. Title compound **9** was obtained as a yellowish solid (1.3 g, 6.0 mmol, 24%).

* 25 g of Dowex ion-exchange resin (50WX8, 16–40 mesh) was added to 50 mL 2 M HCl and stirred for 60 min at room temperature. The resin was collected and washed with H_2O until the filtrate reached a pH of 7. Then, the resin was dried in an oven for 16–24 h at 100 °C.

^{1}H NMR (400 MHz, MeOD-*d*$_4$) δ/ppm = 3.66 (s, 6H), 2.96–2.80 (m, 4H).
Analytical data were consistent with those reported in literature.[2]

Compound 10

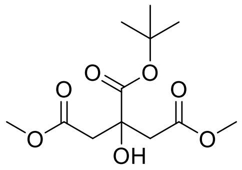

10

The synthesis was performed according to a modified literature procedure.[3]

The reaction flask was charged with compound **9** (1.1 g, 5.0 mmol, 1.0 equiv.). To the reaction mixture was added dropwise 200 µL concentrated perchloric acid. The reaction mixture was stirred for 3 days at room temperature. After completion, the reaction mixture was carefully poured into a saturated solution of 20 mL $NaHCO_3$ and extracted three times with Et_2O. Volatiles were removed under reduced pressure before 30 mL *iso*-hexane were added to the

residue. The reaction mixture was stirred for 2 h and then stored at 4 °C overnight. The by-product (white precipitate) was filtered off and the volatiles were removed under reduced pressure. Compound **10** was obtained as a colourless oil (0.55 g, 2.0 mmol, 40%).

^{1}H NMR (400 MHz, $CDCl_3$) *δ*/ppm = 3.68 (s, 6H), 2.88–2.74 (m, 4H), 1.50 (s, 9H).
Analytical data were consistent with those reported in literature.[3]

Compound 11

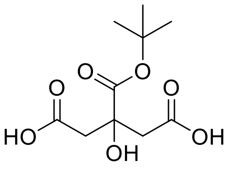

11

The synthesis was performed according to a modified literature procedure.[3]

Compound **10** (0.49 g, 1.77 mmol, 1.00 equiv.) was dissolved in 2.5 mL MeOH. The reaction mixture was cooled down to 0 °C before 2.5 mL of cooled 2 M NaOH was added. The reaction mixture was stirred for 30 min at 0 °C. The cooling was removed and the reaction was stirred for 24 h at room temperature. After completion, the reaction mixture was acidified with 2 M HCl and extracted three times with EtOAc. The volatiles were removed under reduced pressure. Title compound **11** was obtained as a white solid (0.42 g, 1.68 mmol, 95%).

^{1}H NMR (400 MHz, MeOD-*d*$_4$) *δ*/ppm = 2.89–2.70 (m, 4H), 1.48 (s, 9H).
Analytical data were consistent with those reported in literature.[3]

Compound 12a

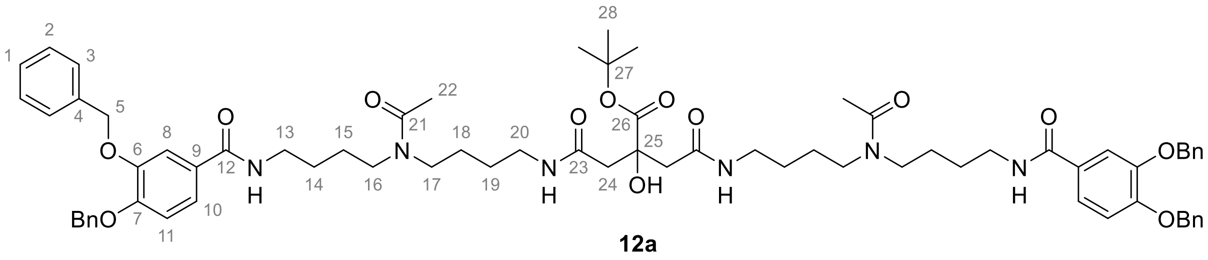

12a

The synthesis was performed according to a modified literature procedure.[2]

In a 25 mL round-bottomed flask compound **11** (68.0 mg, 0.274 mmol, 1.00 equiv.) was dissolved in 5 mL dry THF, followed by the addition of *N*-hydroxysuccinimide (94.6 mg, 0.822 mmol, 3.00 equiv.) and *N,N'*-dicyclohexylcarbodiimide (170 mg, 0.822 mmol,

3.00 equiv.). The reaction mixture was stirred for 3 h at 20 °C. After completion, volatiles were removed under reduced pressure. Then, 5 mL of dry 1,4-dioxane were added to the residue and the reaction mixture was stirred for 15 min at 10 °C and used immediately.

Compound **8a** (299 mg, 578 µmol, 2.10 equiv.) was dissolved in 5 mL dry DCM. Dry TEA (0.30 mL, 2.18 mmol, 7.89 equiv.) was added and the reaction mixture was stirred for 30 min at 0 °C. Then, the reaction mixture was added dropwise to 2-(*tert*-butyl) 1,3-bis(2,5-dioxopyrrolidin-1-yl) 2-hydroxypropane-1,2,3-tricarboxylate (121 mg, 274 µmol, 1.00 equiv.) with a syringe. The cooling was removed and the reaction mixture was stirred for 20 h at 20 °C. After completion, the solids were filtered off and volatiles were removed under reduced pressure. The residue was re-dissolved in 15 mL DCM and the organic layers were washed three times with saturated Na_2CO_3 solution and dried over Na_2SO_4. Volatiles were removed under reduced pressure and the crude product purified by flash column chromatography (silica, gradient from 0% to 10% AM in DCM). Compound **12a** was obtained as a colourless oil (116 mg, 92.6 µmol, 34%).

R_f (DCM/MeOH 95:5) = 0.10.

^{1}H NMR (600 MHz, $CDCl_3$) *δ*/ppm = 7.57 (dd, *J* = 6.8, 2.1 Hz, 1H, 8/10/11-H), 7.53 – 7.51 (m, 1H, 8/10/11-H), 7.44 – 7.40 (m, 8H, 3-H), 7.39 – 7.36 (m, 1H, 8/10/11-H), 7.35 – 7.31 (m, 8H, 2-H), 7.30 – 7.27 (m, 4H, 3-H; 1H, 8/10/11-H), 7.15 – 7.06 (m, 2H, N-H), 6.91 – 6.88 (m, 2H, 8/10/11-H), 6.82 (q, *J* = 5.9 Hz, 1H, N-H), 5.16 – 5.14 (m, 8H, 5-H), 3.44 – 3.39 (m, 4H, 13-H), 3.31 – 3.17 (m, 12H, 16/17/20-H), 2.69 – 2.51 (m, 4H, 24-H), 2.03 – 2.01 (m, 6H, 22-H), 1.57 – 1.43 (m, 16H, 14/15/18/19-H), 1.43 (s, 6H, 28-H), 1.42 (s, 3H, 28-H).

^{13}C NMR (151 MHz, $CDCl_3$) *δ*/ppm = 172.9 – 172.6 (C-23/26), 170.5 – 170.4 (C-21), 170.0 – 169.8 (C-23/26), 167.2 – 167.1 (C-12), 151.71 – 151.5 (C-6/7), 148.7 – 148.6 (C-6/7), 137.0 – 136.7 (C-4), 128.6 – 128.5 (C-2), 128.0 – 127.9 (C-1), 127.7 – 127.6 (C-9), 127.5 – 127.2 (C-3), 120.6 – 120.4 (C-8/10/11), 114.1 – 113.7 (C-8/10/11), 82.7 (C-27), 74.0 (C-25), 71.3 – 71.0 (C-5), 48.7 – 48.5 (C-16/17/20), 45.5 – 45.0 (C-16/17/20), 44.1 – 43.6 (C-24), 39.5 – 39.4 (C-13), 38.8 – 38.6 (C-16/17/20), 27.8 (C-28), 27.1 – 25.1 (C-14/15/18/19), 21.7 – 21.6 (C-22).

HR-MS (ESI): *m/z* calcd for $C_{72}H_{91}N_6O_{13}^+$: 1247.6639 [*M*+H]$^+$; found 1247.6632 (−0.561 ppm).

3,4-Rhodopetrobactin B (13a)

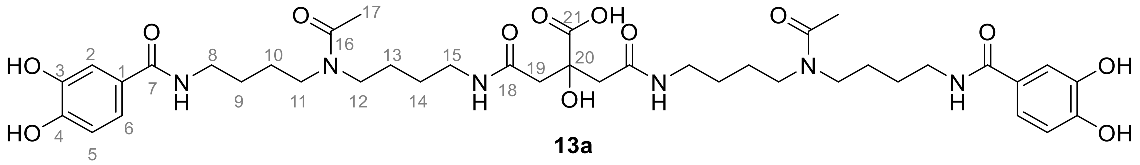

13a

The synthesis was performed according to a modified literature procedure.[1]

In a round-bottomed 25 mL flask under standard atmospheric conditions, compound **12a** (45 mg, 36.1 µmol) was dissolved in 2 mL glacial acetic acid. Then, 2 mL concentrated HCl was dissolved in 3 mL glacial acetic acid and added dropwise using a glass pipette. The reaction mixture was stirred for 2 h at 20 °C. After completion, volatiles were removed under reduced pressure and residual acid was removed by co-evaporation with DCM. The residue was re-dissolved in 5 mL ethanol (EtOH) and 10 mg Pd/C (10 wt.%) was added. The reaction mixture was saturated with H_2 for 15 min and stirred for 2 h at 20 °C. After completion, the solids were filtered off with Celite and washed with EtOH. Volatiles were removed under reduced pressure. The residue was re-dissolved in 5% acetonitrile in water and purified by preparative HPLC (*Dr. Maisch* ReproSil-Pur 120 C18-AQ column (250 × 20mm, 5 µm), A: H_2O + 0.1% TFA, B: acetonitrile + 0.1% TFA, 5 to 32% in 45 min). Rhodopetrobactin B was obtained as a white solid (3 mg, 3.6 µmol,10%). **HPLC: R_t** = 27.7 min.

Table S1 ^{1}H NMR (800 MHz) and **^{13}C NMR** (201 MHz) in DMSO-d_6 of 3,4-Rhodopetrobactin B (**13a**).

C/H	δ_H (ppm)	Multiplicity (*J* in Hz)	δ_C (ppm)	HMBC contacts
**1**			125.95	
**2**	7.26	d (2.2)	115.06	C1, C3, C4, C6, C7
**3**			144.77	
**4**			148.15	
**5**	6.74	ddd (8.2, 4.3, 1.3)	114.78	C1, C2, C3, C4, C6, C7
**6**	7.16	dd (8.2, 2.1)	118.83	C2, C3, C4, C7
**7**			166.10	
**8**	3.19, 3.22	m	38.59, 38.64	C7, C9, C10, C11
**9**	1.35/1.47	m	25.43/25.99	C8, C10, C11
**10**	1.35/1.47	m	25.43/25.99	C8, C9, C11
**11**	3.19, 3.22	m	44.25, 47.31	C9, C10, C12, C16
**16**			169.10	
**17**	1.96	m	21.32	C11, C12, C16
**12**	3.19, 3.22	m	44.25, 47.31	C11, C13, C14, C16
**13**	1.30/1.41/1.47	m	24.70/26.17/26.36/26.54	C12, C14, C15
**14**	1.30/1.41/1.47	m	24.70/26.17/26.36/26.54	C12, C13, C15
**15**	3.00, 3.04	m	37.85	C13, C14
**18**			169.54	
**19**	2.48, 2.57	m	43.13	C18, C19, C20
**20**			73.52	
**21**			174.98	

HR-MS ESI: *m/z* calcd for $C_{40}H_{59}N_6O_{13}^+$ 831.4135 [M+H]$^+$; found 831.4127 (−0.962 ppm).

Compound 6b

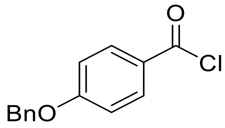

6b

In a 25 mL round-bottomed flask 4-benzyloxybenzoic acid (100 mg, 440 µmol, 1.00 equiv.) was dissolved in 5 mL dry DCM and 2 mL dry toluene before oxalyl chloride (100 µL, 1.17 mmol, 2.65 equiv.) was added. Afterwards DMF was added in portions of 3 drops in time intervals of 30 min over 2 h at 22 °C using a syringe until no further gas evolution was observed. The reaction was then stopped immediately and volatiles were removed under reduced pressure, resulting in a yellowish solid. The crude product was used immediately in the synthesis of compound **7b** without further purification.

Compound 7b

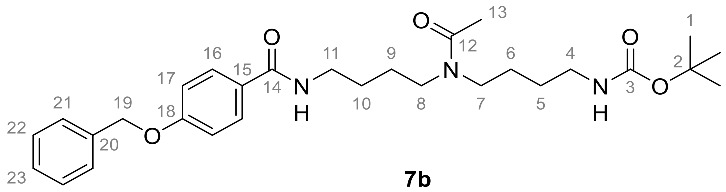

7b

In a 50 mL round-bottomed flask compound **4** (88.0 mg, 292 µmol, 1.00 equiv.) was dissolved in 5 mL dry DCM and dry TEA (100 µL, 717 µmol, 2.46 equiv.). Compound **6b** (108 mg, 438 µmol, 1.50 equiv.) was dissolved in 3 mL dry DCM and transferred with a syringe dropwise to the other solution. The mixture turned from colorless to brown. The reaction was stirred at 23 °C for 3 h. Volatiles were removed under reduced pressure and the crude product was purified by column chromatography (silica, gradient from 2% to 10% AM in DCM) yielding compound **7b** as colorless oil (56.0 mg, 109 µmol, 37% yield).

$\mathbf{R_f}$ (DCM/AM 9:1) = 0.40.

^{1}H NMR (600 MHz, CDCl$_3$) *δ/ppm* = 7.83 – 7.72 (m, 2H, 16-H), 7.42 – 7.39 (m, 2H, 21-H), 7.37 (ddd, *J* = 7.6, 6.6, 1.3 Hz, 2H, 22-H), 7.33 – 7.29 (m, 1H, 23-H), 6.96 (dd, *J* = 8.9, 2.3 Hz, 2H, 17-H), 5.08 (d, *J* = 3.5 Hz, 2H, 19-H), 4.76 (dd, *J* = 19.7, 13.4 Hz, 1H, N-H), 3.45 (dq, *J* = 7.9, 5.9, 5.5 Hz, 2H, 11-H), 3.30 (dt, *J* = 28.6, 7.2 Hz, 2H, 7/8-H), 3.23 (dt, *J* = 13.0, 5.1 Hz, 2H, 7/8-H), 3.10 (dq, *J* = 18.0, 6.8 Hz, 2H, 4-H), 2.05 (d, *J* = 10.0 Hz, 3H, 13-H), 1.66 – 1.55 (m, 6H, 5/6/9/10-H), 1.49 – 1.44 (m, 2H, 5/6/9/10-H), 1.41 (d, *J* = 4.8 Hz, 9H, 1-H).

^{13}C NMR (151 MHz, $CDCl_3$) δ/ppm = 170.5 (C-12), 167.2 (C-14), 161.3 (C-18), 156.2 (C-3), 136.5 (C-20), 128.9 (C-16), 128.7 (C-22), 128.2 (C-23) 127.5 (C-21), 127.3 – 127.0 (C-15), 114.7 (C-17), 79.3 (C-2), 70.1 (C-19), 48.6 (C-7), 45.2 (C-8), 40.1 – 39.9 (C-4), 39.4 (C-11), 28.5 (C-1), 28.1 (C-5/6/9/10), 27.6 (C-5/6/9/10), 27.2 (C-5/6/9/10), 26.3 (C-5/6/9/10), 26.0 (C-5/6/9/10), 25.3 (C-5/6/9/10), 24.9 (C-5/6/9/10), 21.6 (C-13).

HR-MS (ESI): *m/z* calcd for $C_{29}H_{42}N_3O_5^+$: 512.3119 [M+H]$^+$; found: 512.3124 (+0.976 ppm).

Compound 8b

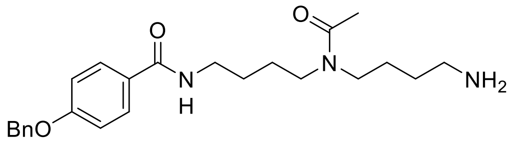

8b

In a 10 mL round-bottomed flask under standard atmospheric conditions, compound **7b** (55.0 mg, 107 µmol, 1.00 equiv.) was dissolved in 5 mL DCM and cooled to 0 °C. Afterwards, TFA (200 µL, 2.61 mmol, 24.3 equiv.) was added in two 100 µL portions: one at the start and another after 1.5 h. Then, the cooling was removed and the solution stirred for 17 h at 23 °C. Volatiles were removed under reduced pressure and the crude product was immediately used without further purification for the synthesis of compound **12b**.

Compound 12b

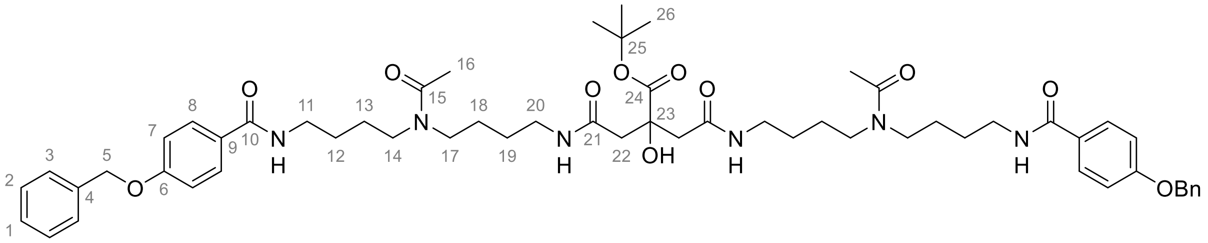

12b

This synthesis was performed according to a modified literature procedure.[1]

In a 5 mL round-bottomed flask compound **11** (12.2 mg, 49.1 µmol, 1.00 equiv.) was dissolved in 1 mL dry THF. *N*-Hydroxysuccinimide (17.0 mg, 147 µmol, 3.00 equiv.) and *N,N'*-dicyclohexylcarbodiimide (30.4 mg, 147 µmol, 3.00 equiv.) were dried in separate 5 mL round-bottomed flasks before each was dissolved in 0.5 mL dry THF and transferred with a syringe to the dissolved pentanedioic acid. The solution was stirred for 3 h at 22 °C. After completion, volatiles were removed under reduced pressure. Then, 2 mL dry 1,4-dioxane was added and the mixture was cooled to 10 °C.

In a 25 mL round-bottomed flask compound **8b** (44.2 mg, 107 μmol, 2.18 equiv.) was dissolved in 2 mL dry DCM and TEA (100 μL, 721 μmol, 14.7 equiv.). The solution was transferred dropwise with a syringe to 4-(2,5-dioxopyrrolidin-1-yl)oxy-2-[2-(2,5-dioxopyrrolidin-1-yl)oxy-2-oxoethyl]-2-hydroxy-4-oxobutanoic acid (19.0 mg, 49.2 μmol, 1.00 equiv.). The cooling was removed and the reaction mixture was stirred for 66 h at 23 °C. After completion, the solids were filtered off and volatiles were removed under reduced pressure. The residue was re-dissolved in 20 mL DCM and the organic layer was washed three times with saturated Na_2CO_3 solution and dried over Na_2SO_4. Volatiles were removed under reduced pressure and the crude product purified by flash column chromatography (silica, gradient from 0% to 12% AM in DCM). Compound **12b** was obtained as colourless oil (28.0 mg, 27.0 μmol, 55% yield).

$\mathbf{R_f}$ (DCM/AM 9:1) = 0.34.

^{1}H NMR (600 MHz, CDCl$_3$) *δ*/ppm = 7.82 – 7.71 (m, 4H, 8-H), 7.41 (d, *J* = 7.6 Hz, 4H, 3-H), 7.40 – 7.35 (m, 4H, 2-H), 7.36 – 7.30 (m, 2H, 1-H), 6.99 – 6.96 (m, 4H, 7-H), 5.09 (d, *J* = 2.8 Hz, 4H, 5-H), 3.46 – 3.41 (m, 4H, 11-H), 3.36 – 3.16 (m, 12H, 14/17/20-H), 2.73 – 2.52 (m, 4H, 22-H), 2.05 – 2.03 (m, 6H, 16-H), 1.64 – 1.46 (m, 16H, 12/13/18/19-H), 1.44 (d, *J* = 6.4 Hz, 9H, 26-H).

^{13}C NMR (151 MHz, CDCl$_3$) *δ*/ppm = 172.9 (C-24), 170.5 (C-15), 170.03 (C-21), 169.8 (C-10), 167.3 (C-10), 161.4 (C-6), 136.5 (C-4), 129.0 (C-8), 128.8 (C-2), 128.32 (C-1), 127.6 (C-3), 127.24 (C-9), 114.7 (C-7), 82.8 (C-25), 74.1 (C-23), 70.2 (C-5), 48.8 (C-14/17/20), 45.6 – 45.1 (C-14/17/20), 44.19 – 43.68 (C-22), 39.5 (C-11), 38.8 (C-14/17/20), 27.9 (C-26), 27.3 – 25.2 (C-12/13/18/19), 21.73 (C-16).

HR-MS (ESI): *m/z* calcd for $C_{58}H_{78}N_6O_{11}^+$: 1035.5801 $[M+H]^+$; found: 1035.5799 (−0.193 ppm).

Methylolanthanin (13b)

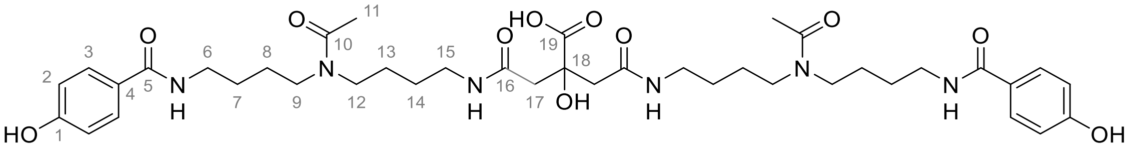

13b

In a 5 mL round-bottomed flask under standard atmospheric conditions, compound **12b** (10.0 mg, 9.66 µmol, 1.00 equiv.) was dissolved in 1 mL acetic acid and 1 mL concentrated HCl. The mixture was stirred for 68 h at 23 °C and the volatiles were removed under reduced pressure. The crude product was re-dissolved in 5 mL H_2O:ACN (1:1) and purified by HPLC (*Dr. Maisch* ReproSil Gold 120 C18 column (250 × 20mm, 5 µm) A = H_2O + 0.1% formic acid (FA), B = ACN + 0.1% FA, 2% to 98% in 25 min). Methylolanthanin **13b** was obtained as white solid (1.00 mg, 1.25 µmol, 13% yield). HPLC: $\mathbf{R_t}$ = 12.8 min.

^{1}H NMR (800 MHz, D_2O+0.003% TMSP-*d₄*) *δ*/ppm = 7.67 – 7.66 (m, 4H, 3-H), 6.93 – 6.82 (m, 4H, 2-H), 3.39 – 3.25 (m, 12H, 6/9/12/15-H), 3.10 – 3.07 (m, 4H, 6/9/12/15-H), 2.67 – 2.55 (m, 4H, 17-H), 2.08 (m, 6H, 11-H), 1.64 – 1.38 (m, 16H, 7/8/13/14-H).

^{13}C NMR (201 MHz, D_2O+0.003% TMSP-d_4) *δ*/ppm = 182.0 (C-16/19), 176.5 (C-10), 175.0 (C-16/19), 173.0 (C-5), 162.8 (C-1), 132.1 (C-3), 127.8 (C-4), 118.4 (C-2), 77.8 (C-18), 51.7 (C-6/9/12/15), 48.5 (C-6/9/12/15), 47.2 (C-17), 42.2 – 42.1 (C-6/9/12/15), 41.7 – 41.6 (C-6/9/12/15), 28.7 – 27.1 (C-7/8/13/14), 23.4 (C-11).

HR-MS (ESI): *m/z* calcd for $C_{40}H_{58}N_6O_{11}^+$: 799.4236[M+H]$^+$; found: 799.4237 (+0.125 ppm).

1.3 NMR Spectra

^{1}H NMR Spectrum of Compound **1** in $CDCl_3$ (400 MHz)

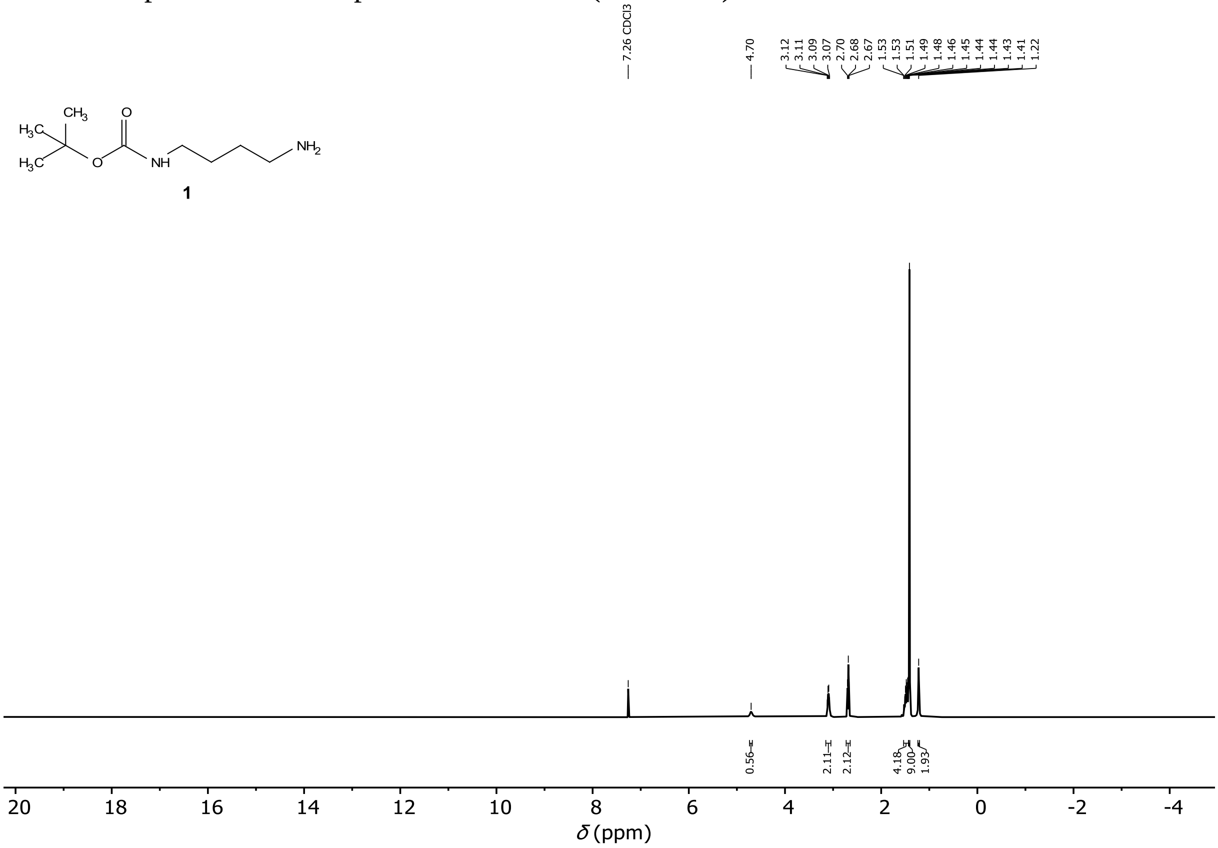

^{13}C NMR Spectrum of Compound **1** in $CDCl_3$ (101 MHz)

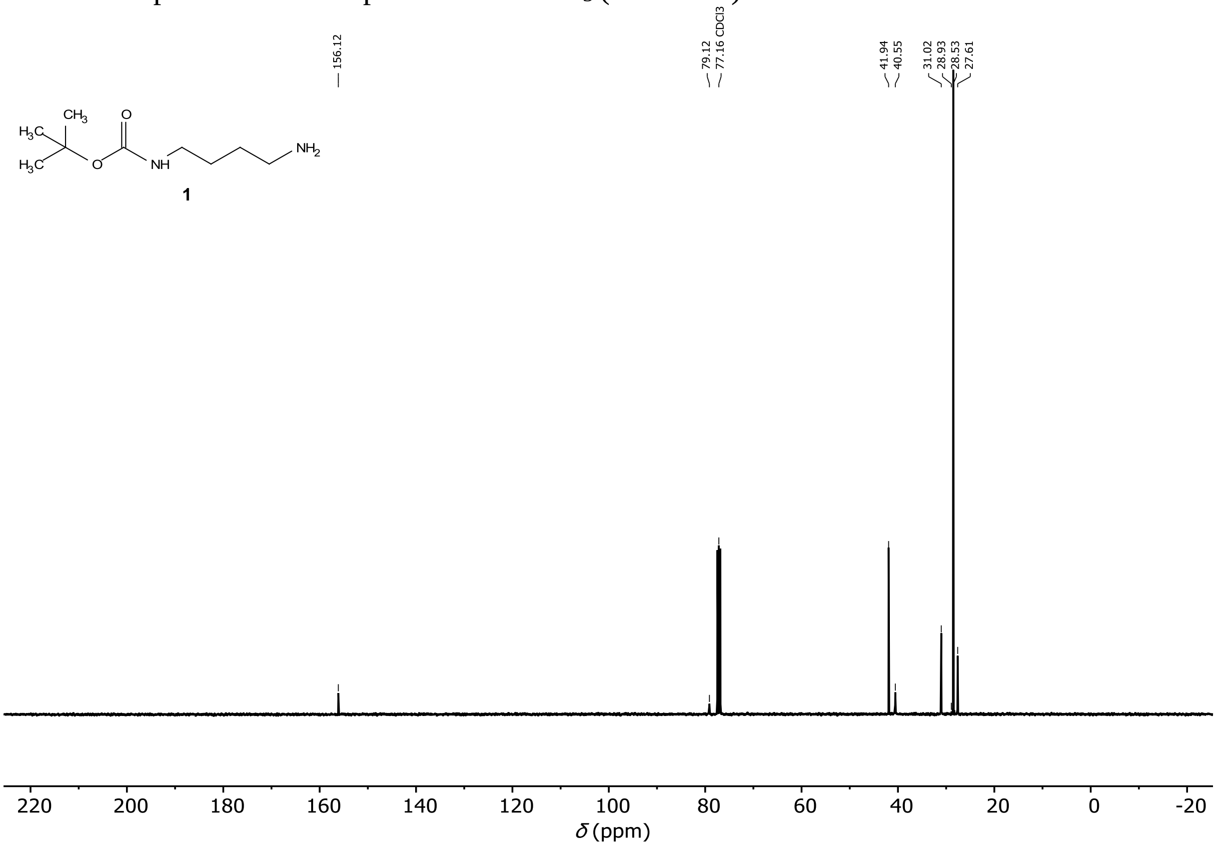

^{1}H NMR Spectrum of Compound **2** in $CDCl_3$ (600 MHz)

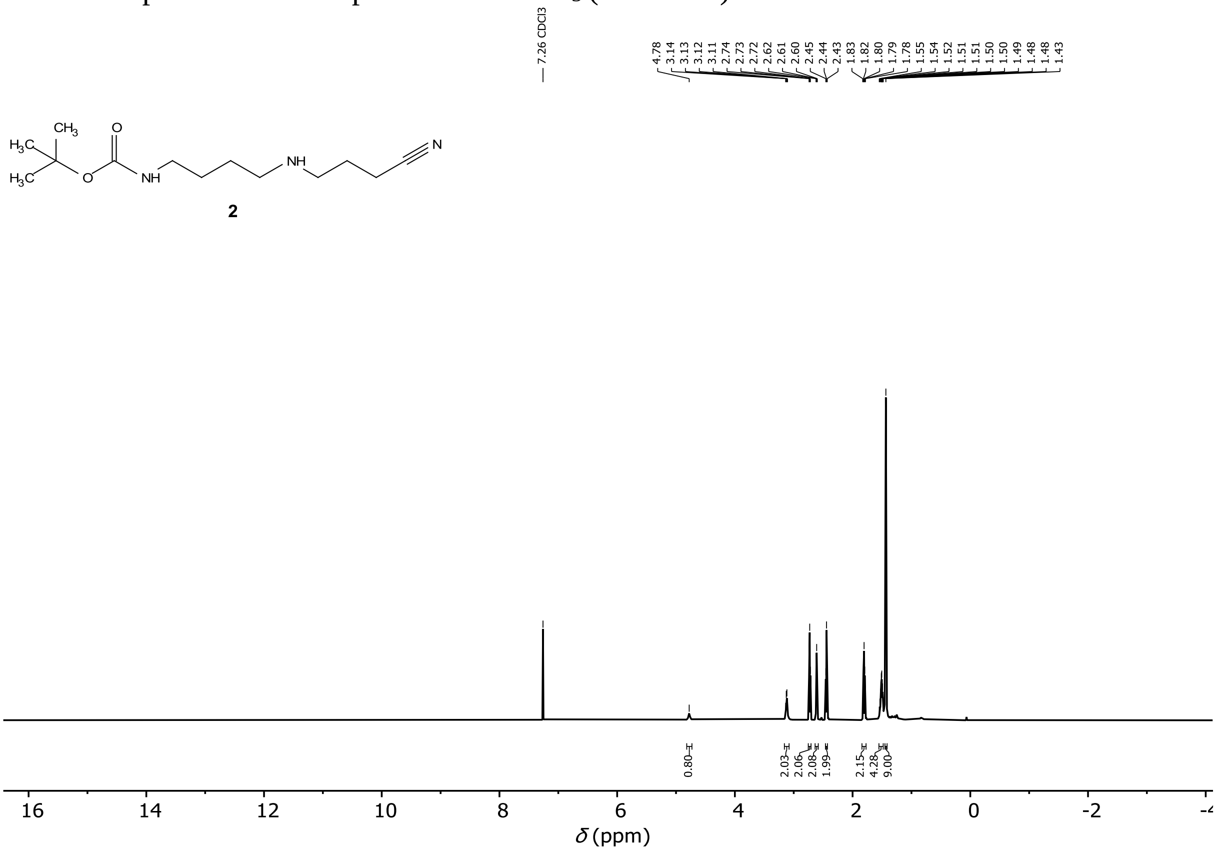

^{13}C NMR Spectrum of Compound **2** in $CDCl_3$ (151 MHz)

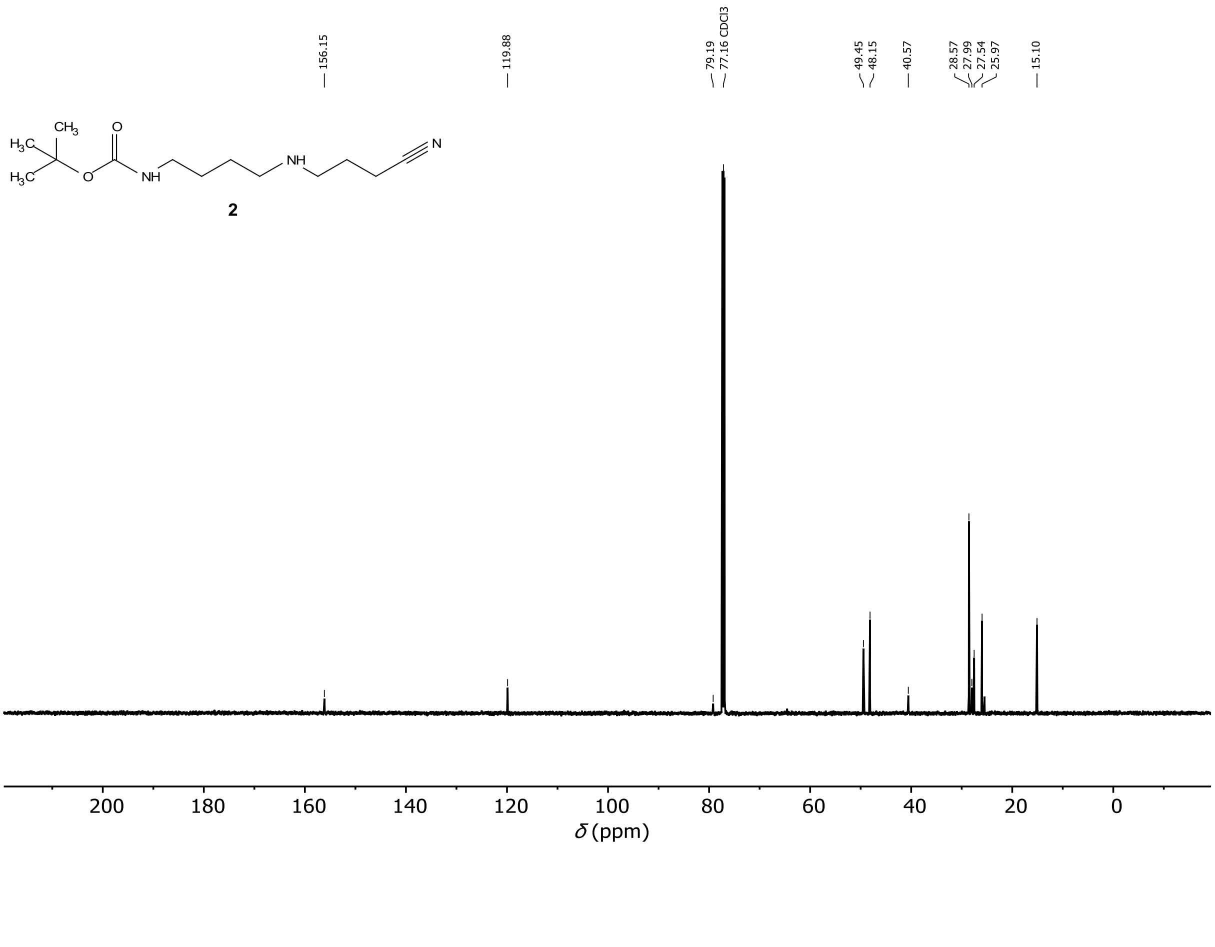

^{1}H NMR Spectrum of Compound **3** in $CDCl_3$ (600 MHz)

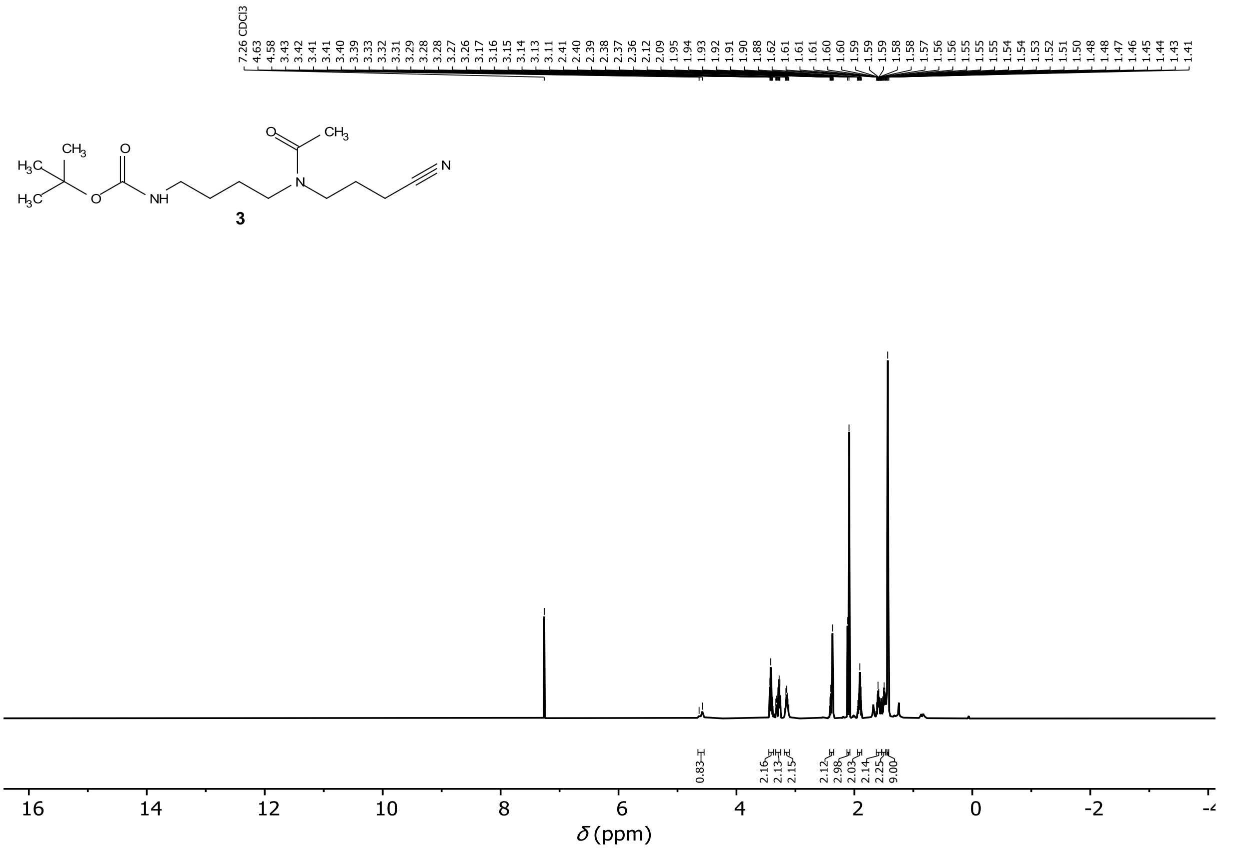

^{13}C NMR Spectrum of Compound **3** in $CDCl_3$ (151 MHz)

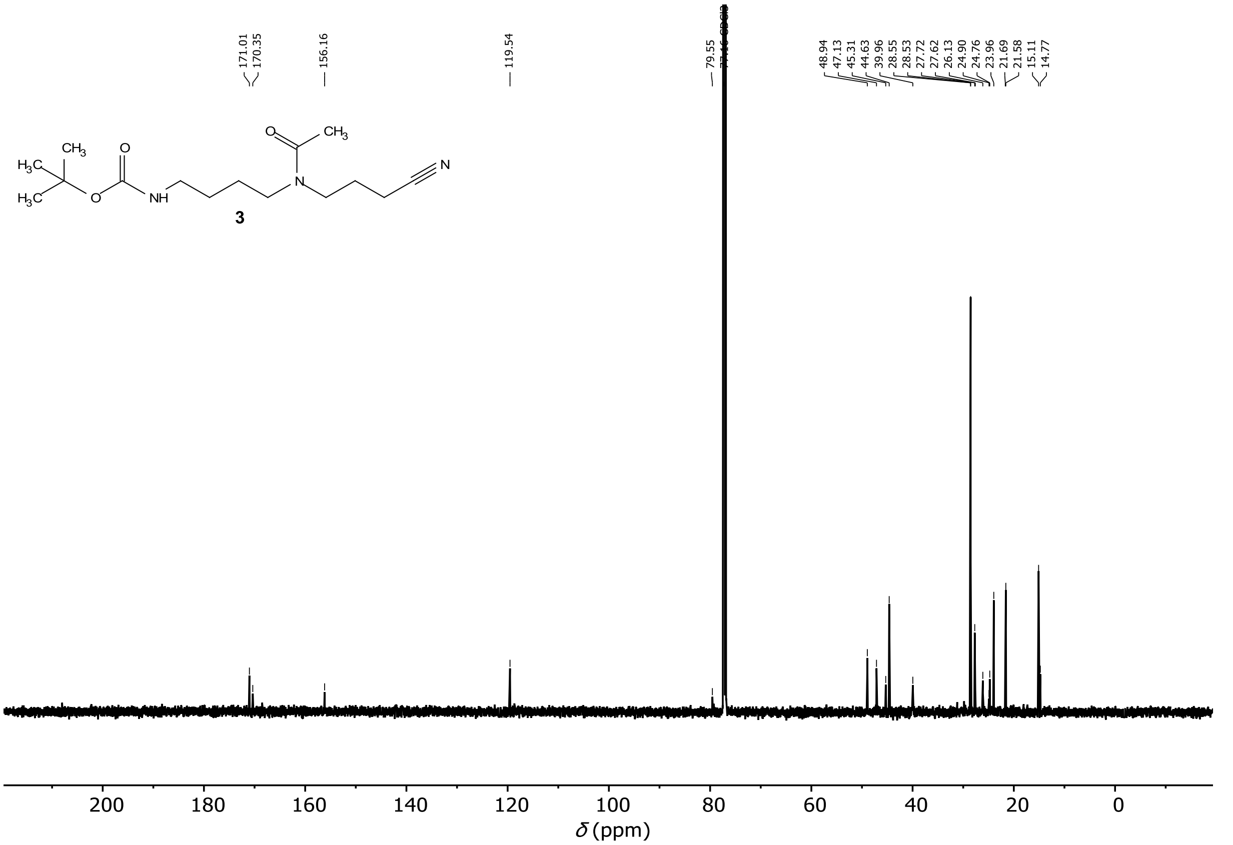

^{1}H NMR Spectrum of Compound **4** in $CDCl_3$ (600 MHz)

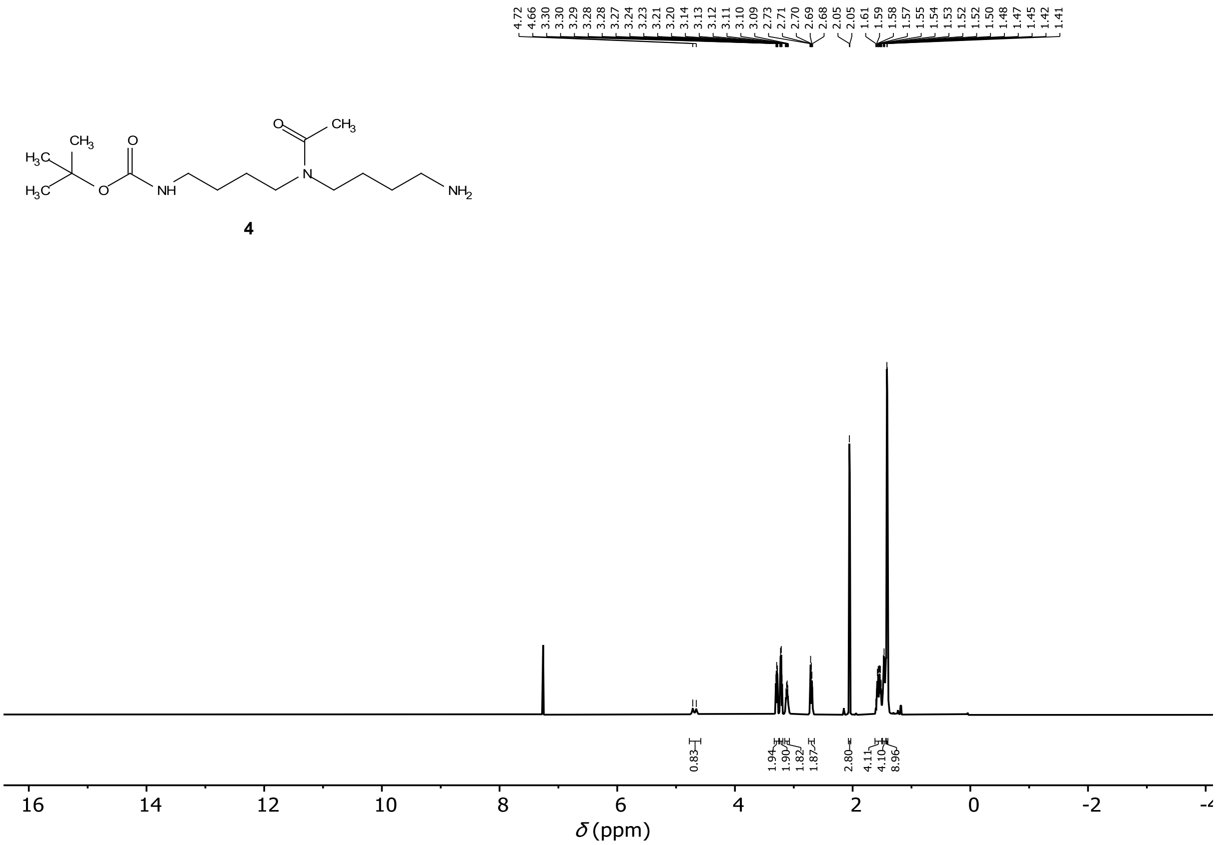

^{13}C NMR Spectrum of Compound **4** in $CDCl_3$ (151 MHz)

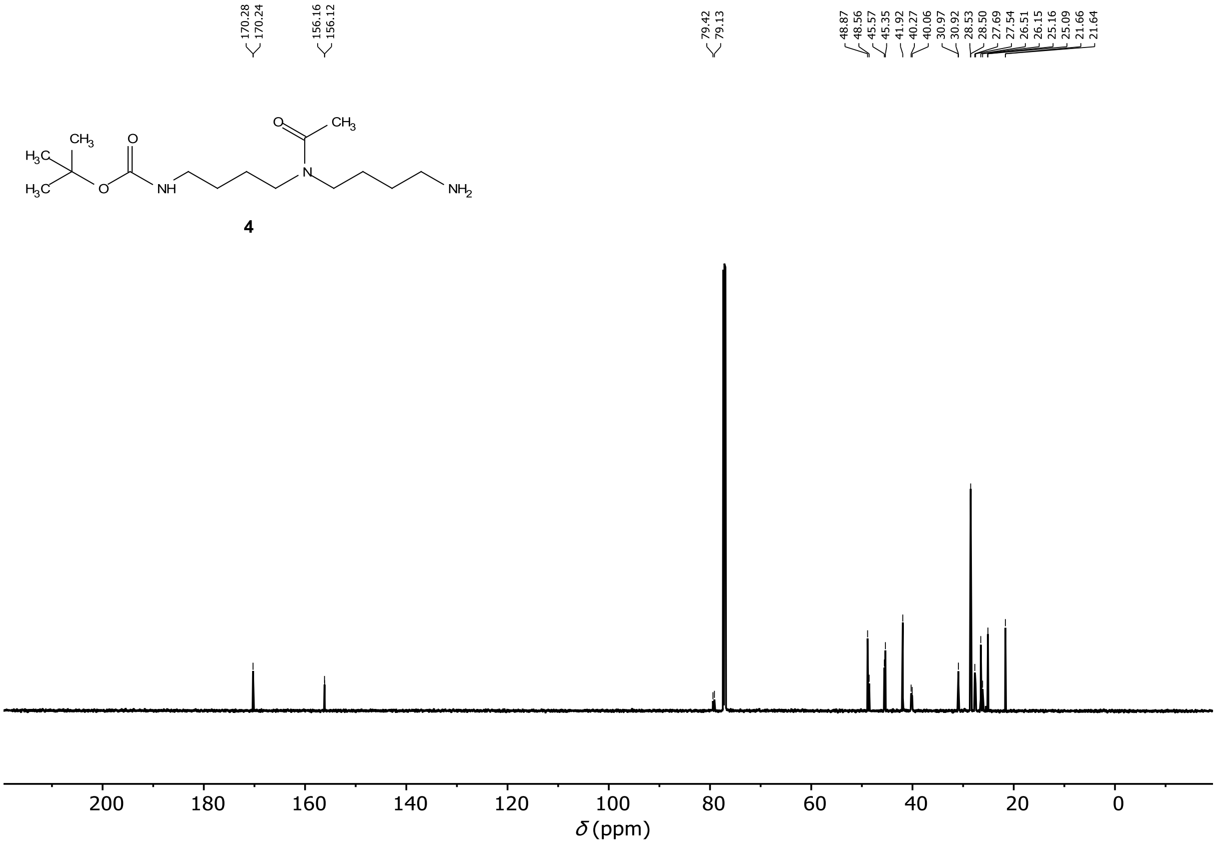

^{1}H NMR Spectrum of Compound **5a** in DMSO-d_6 (600 MHz)

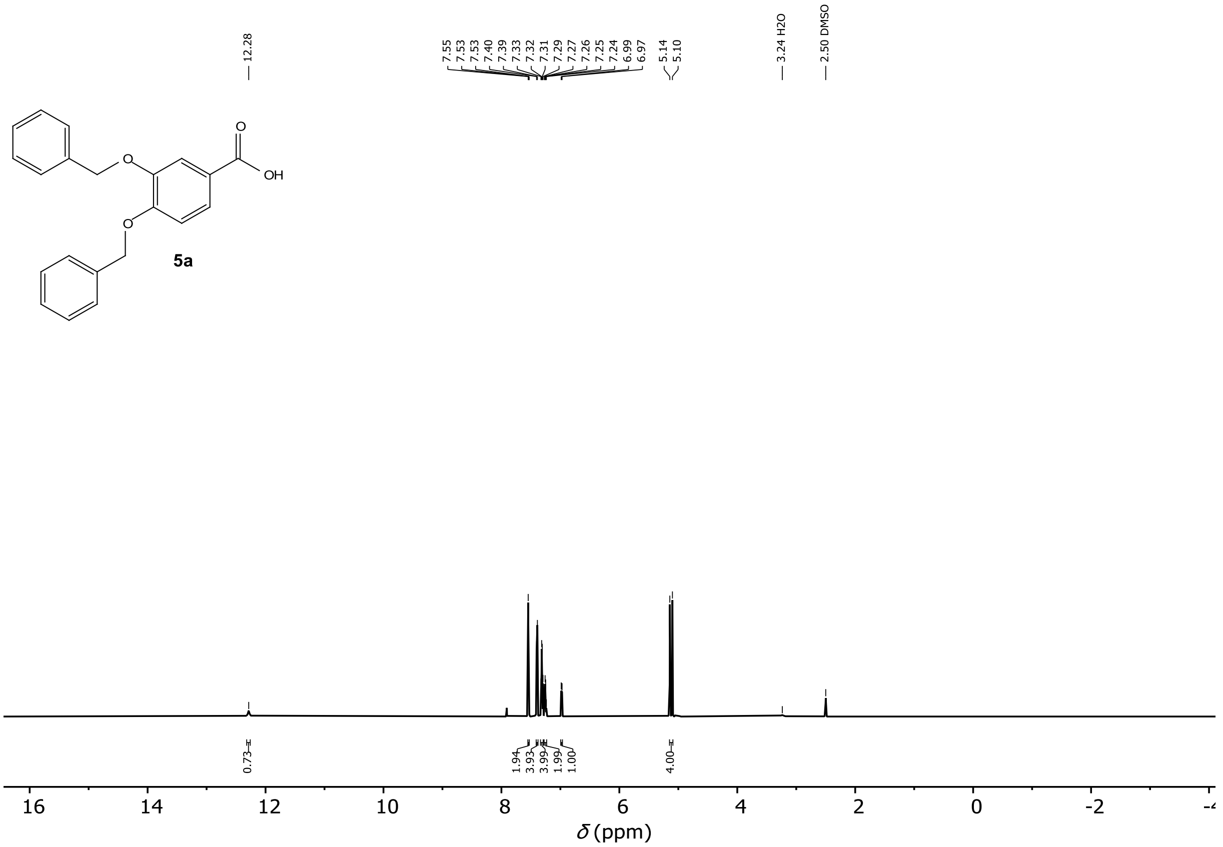

^{13}C NMR Spectrum of Compound **5a** in DMSO-d_6 (151 MHz)

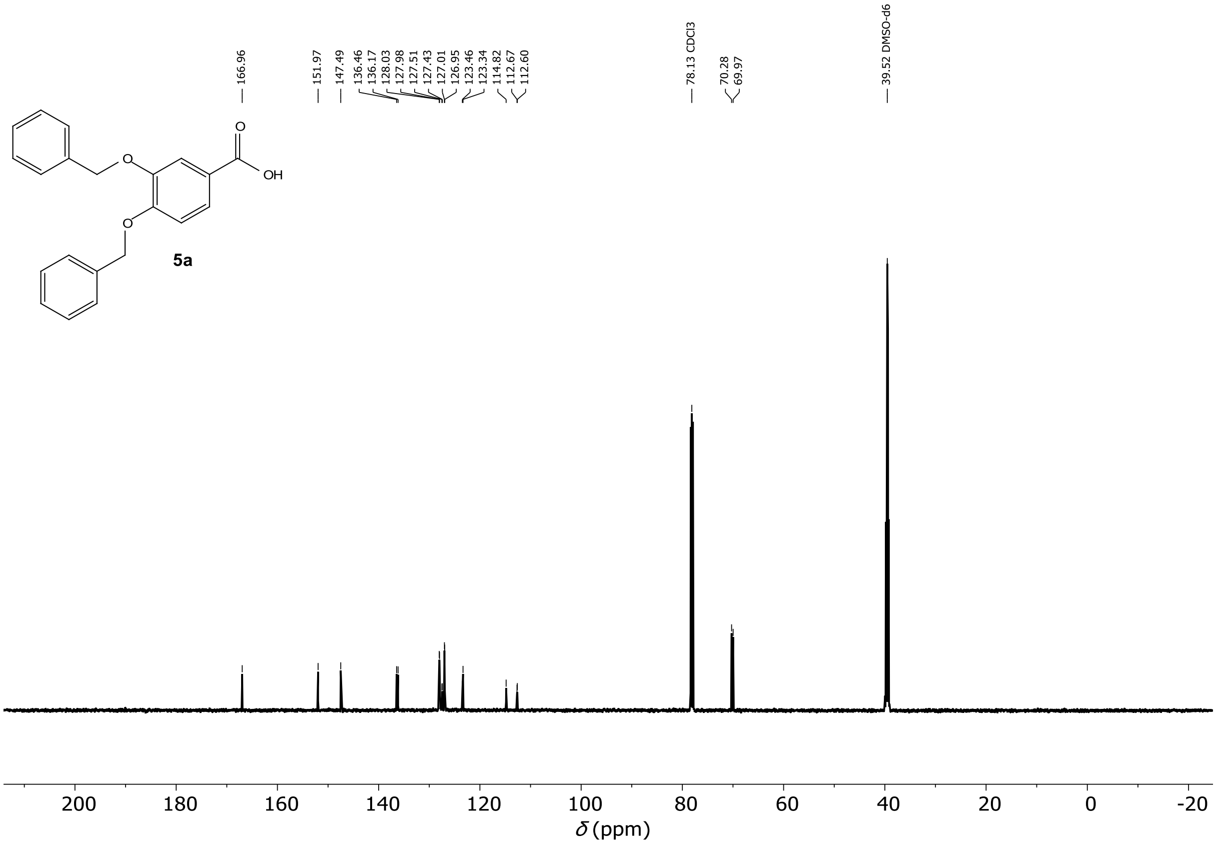

^{1}H NMR Spectrum of Compound **7a** in $CDCl_3$ (600 MHz)

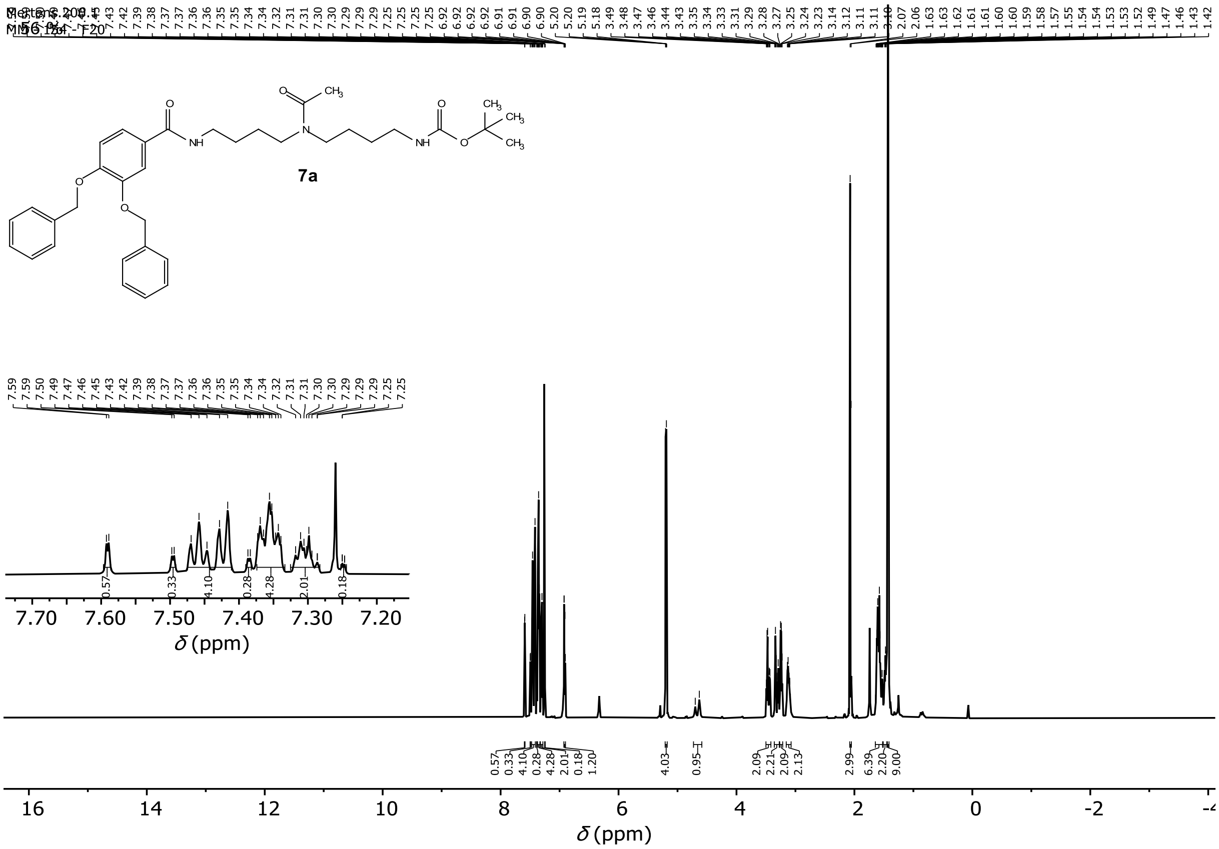

^{13}C NMR Spectrum of Compound **7a** in $CDCl_3$ (151 MHz)

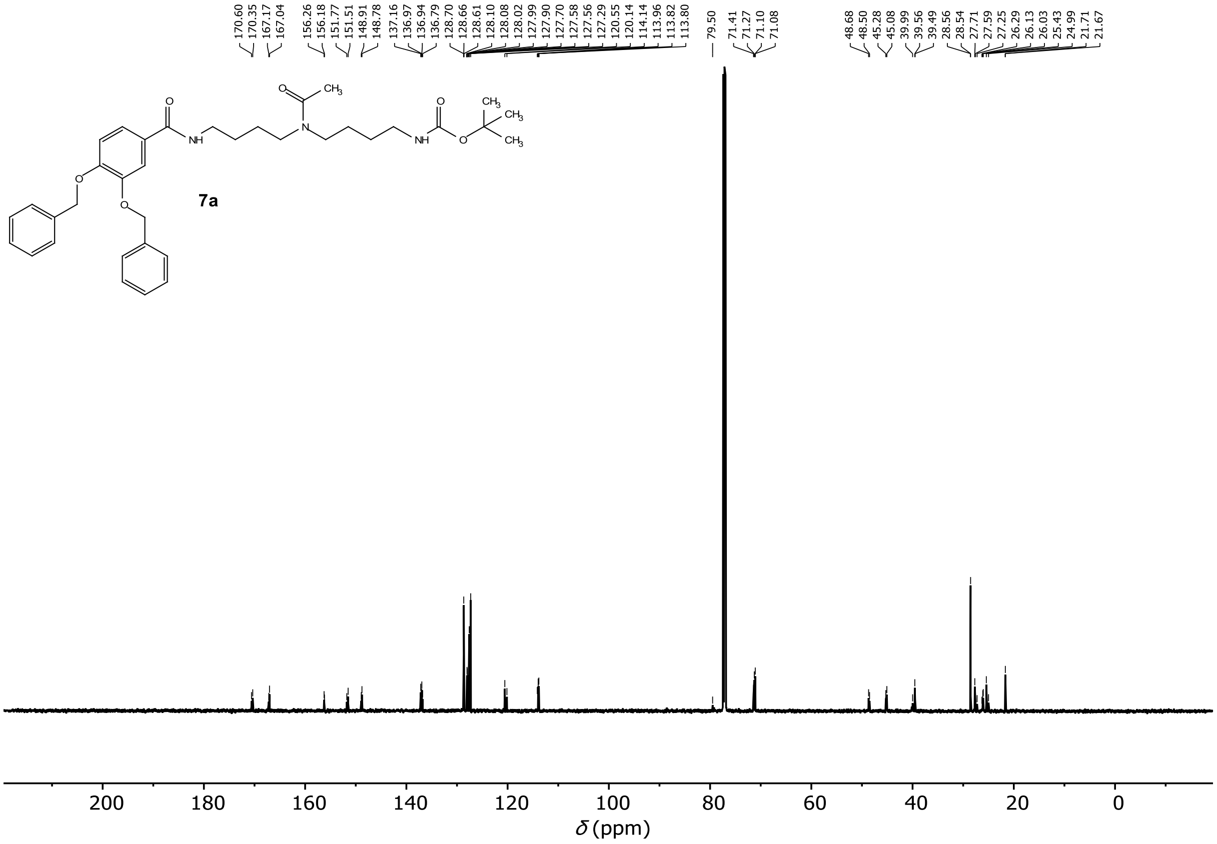

^{1}H NMR Spectrum of Compound **7b** in $CDCl_3$ (600 MHz)

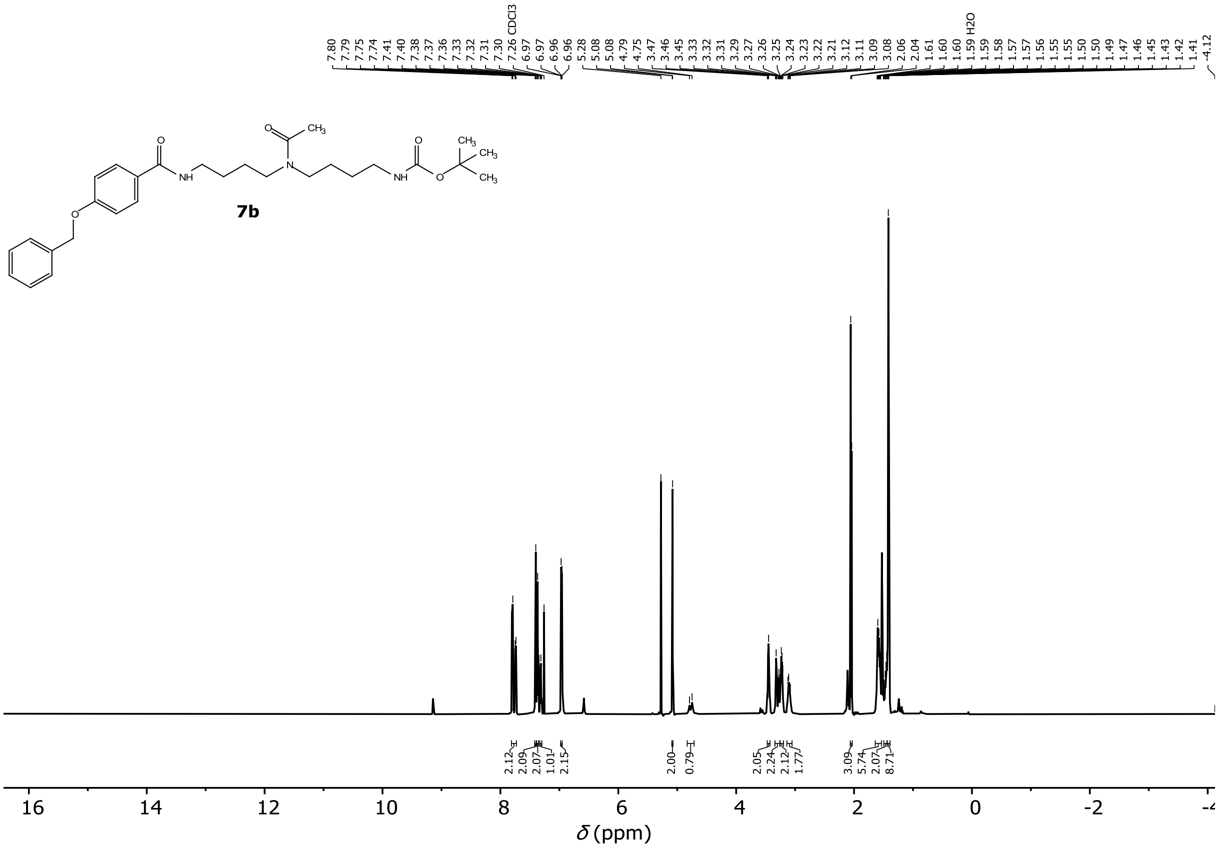

^{13}C NMR Spectrum of Compound **7b** in $CDCl_3$(151 MHz)

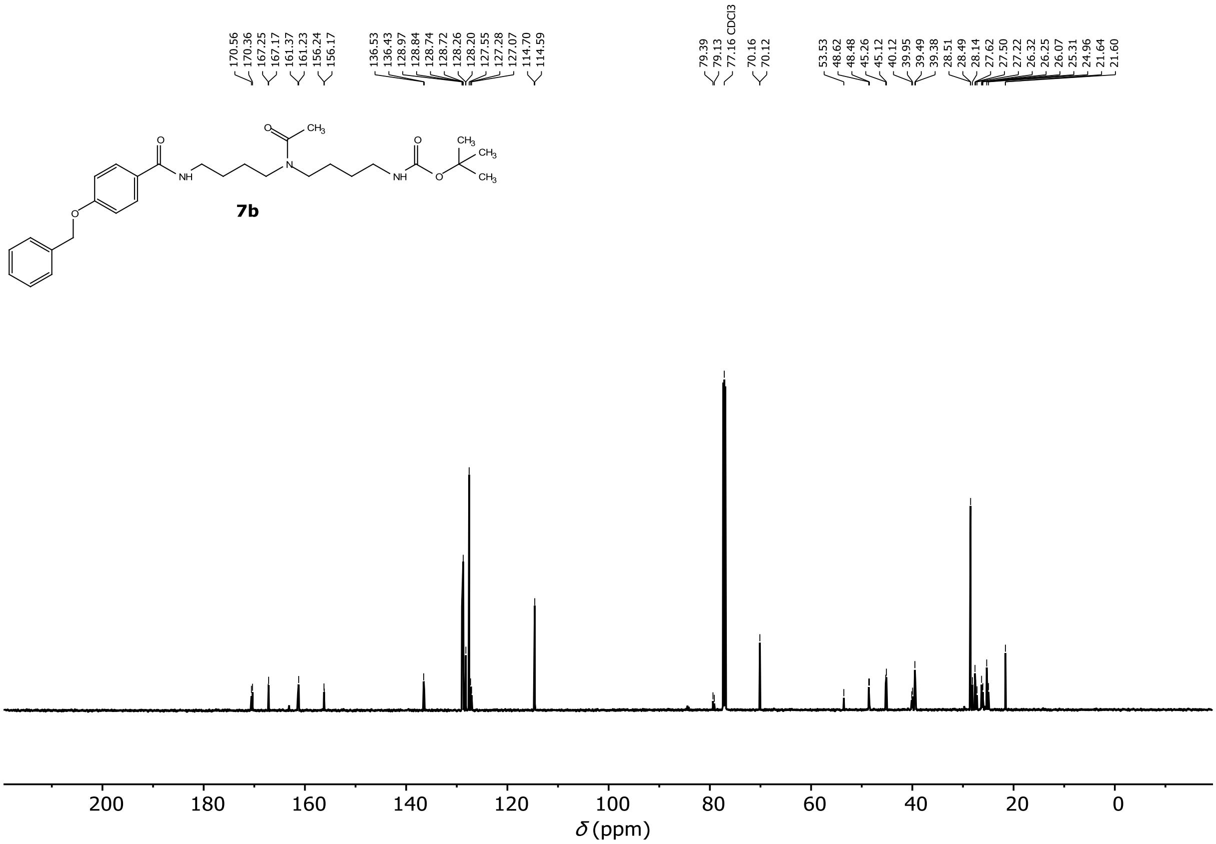

^{1}H NMR Spectrum of Compound **9** in MeOD-d_4 (400 MHz)

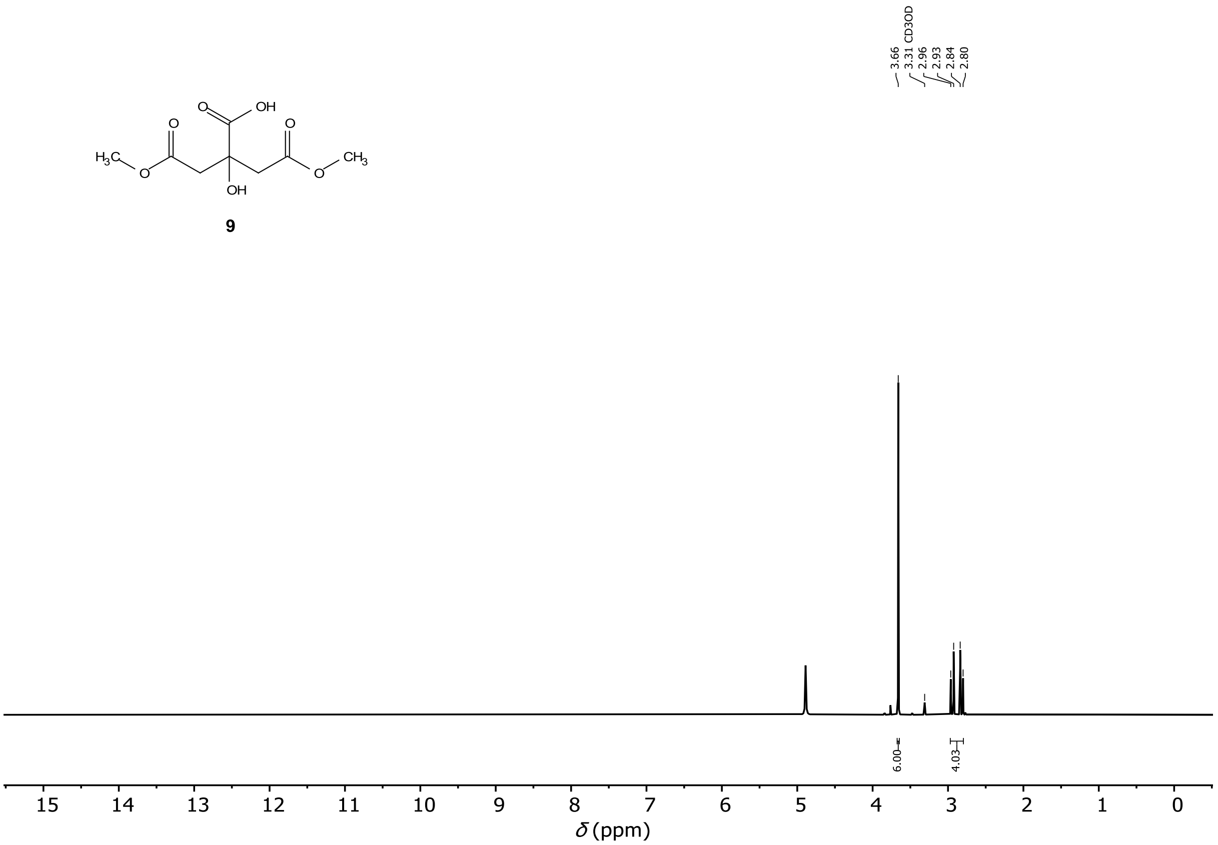

^{1}H NMR Spectrum of Compound **10** in $CDCl_3$ (400 MHz)

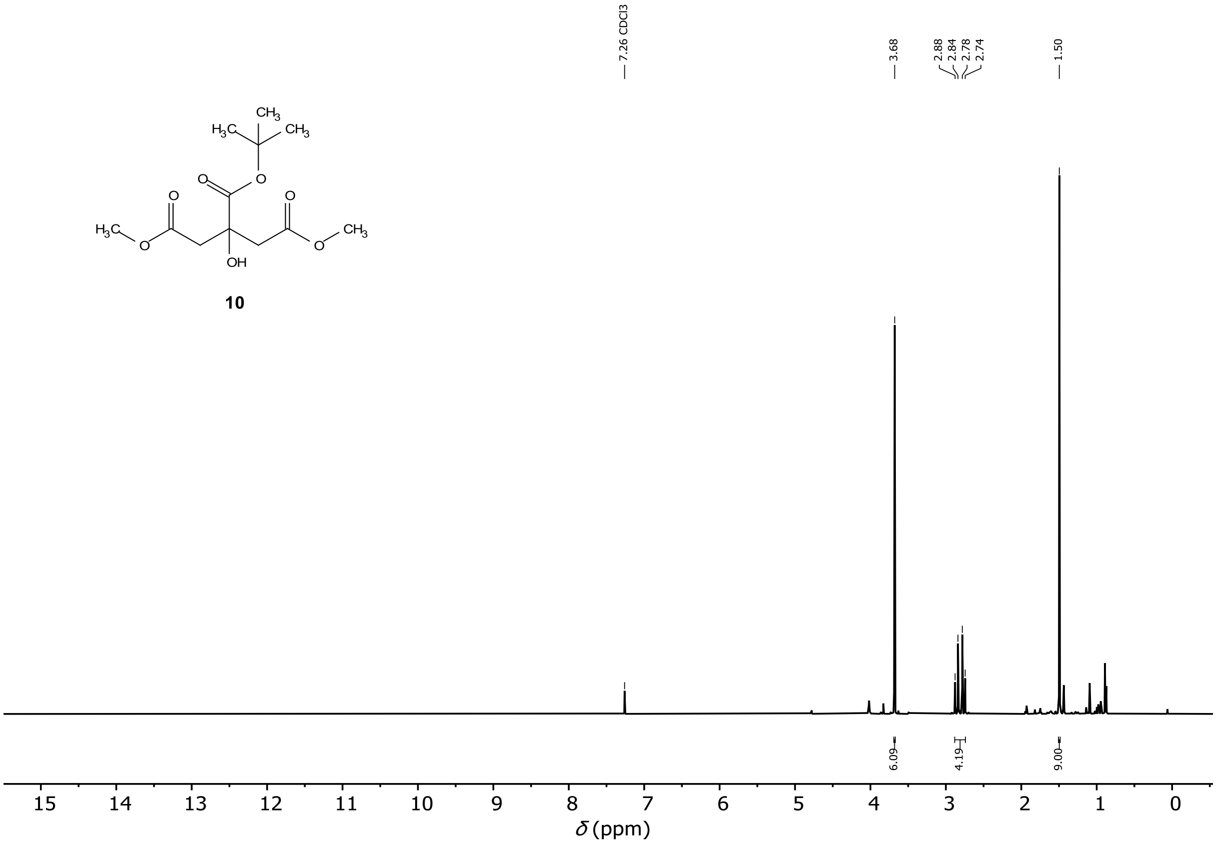

^{1}H NMR Spectrum of Compound **11** in MeOD-d$_4$ (400 MHz)

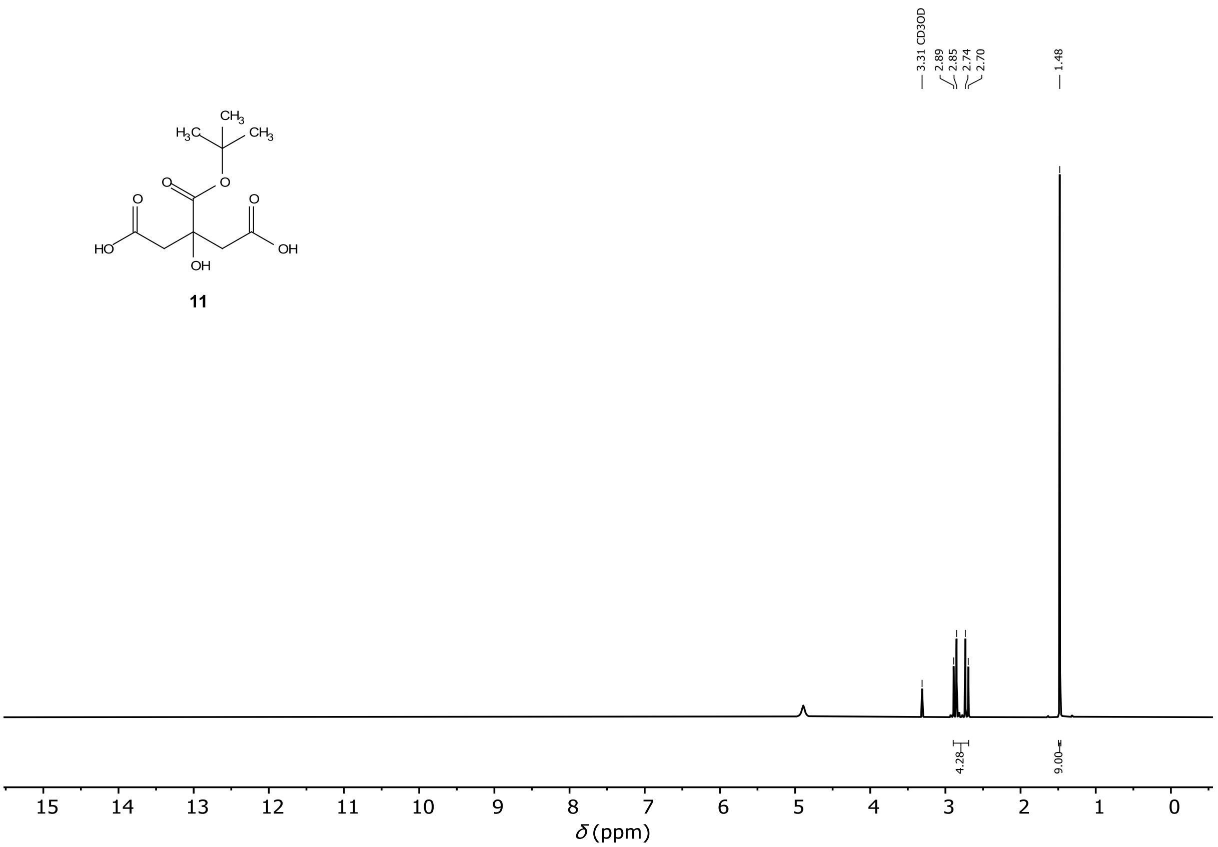

^{1}H NMR Spectrum of Compound **12a** in $CDCl_3$ (600 MHz)

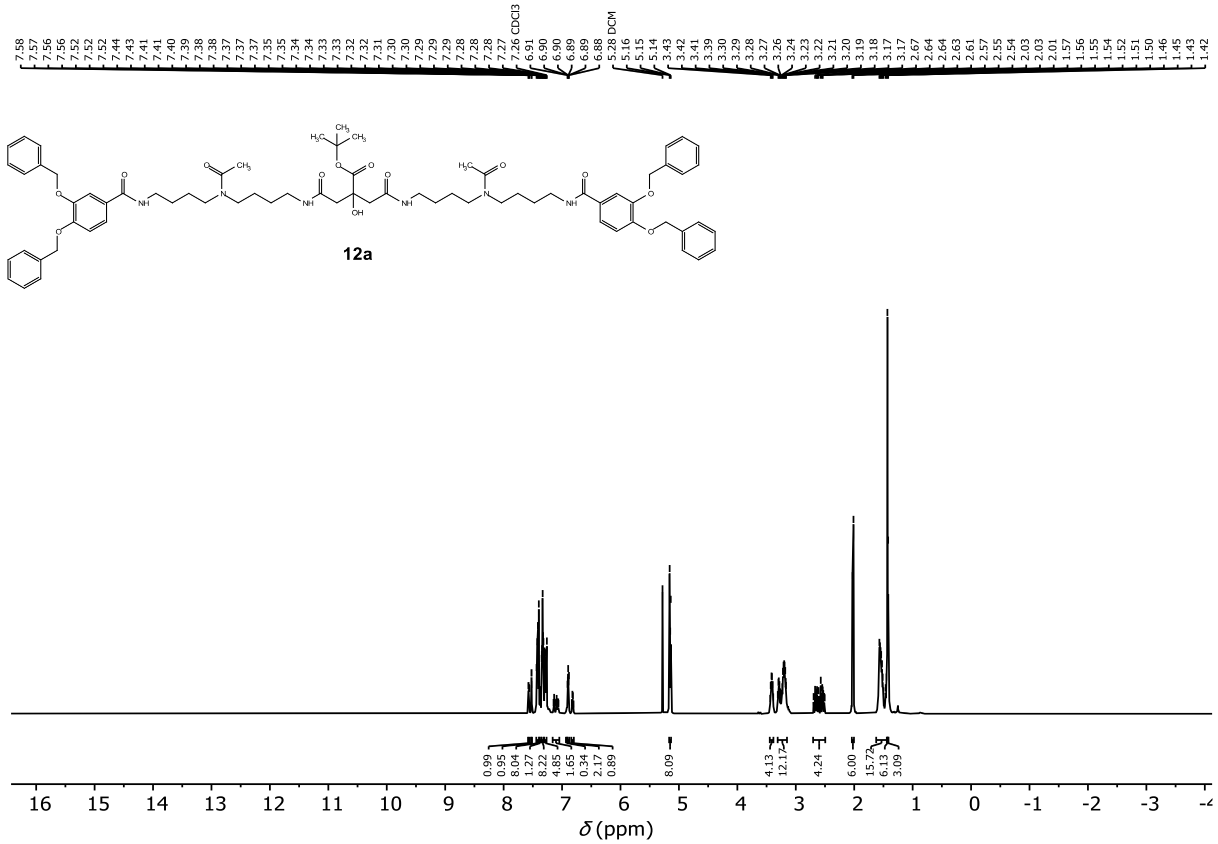

^{13}C NMR Spectrum of Compound **12a** in $CDCl_3$ (151 MHz)

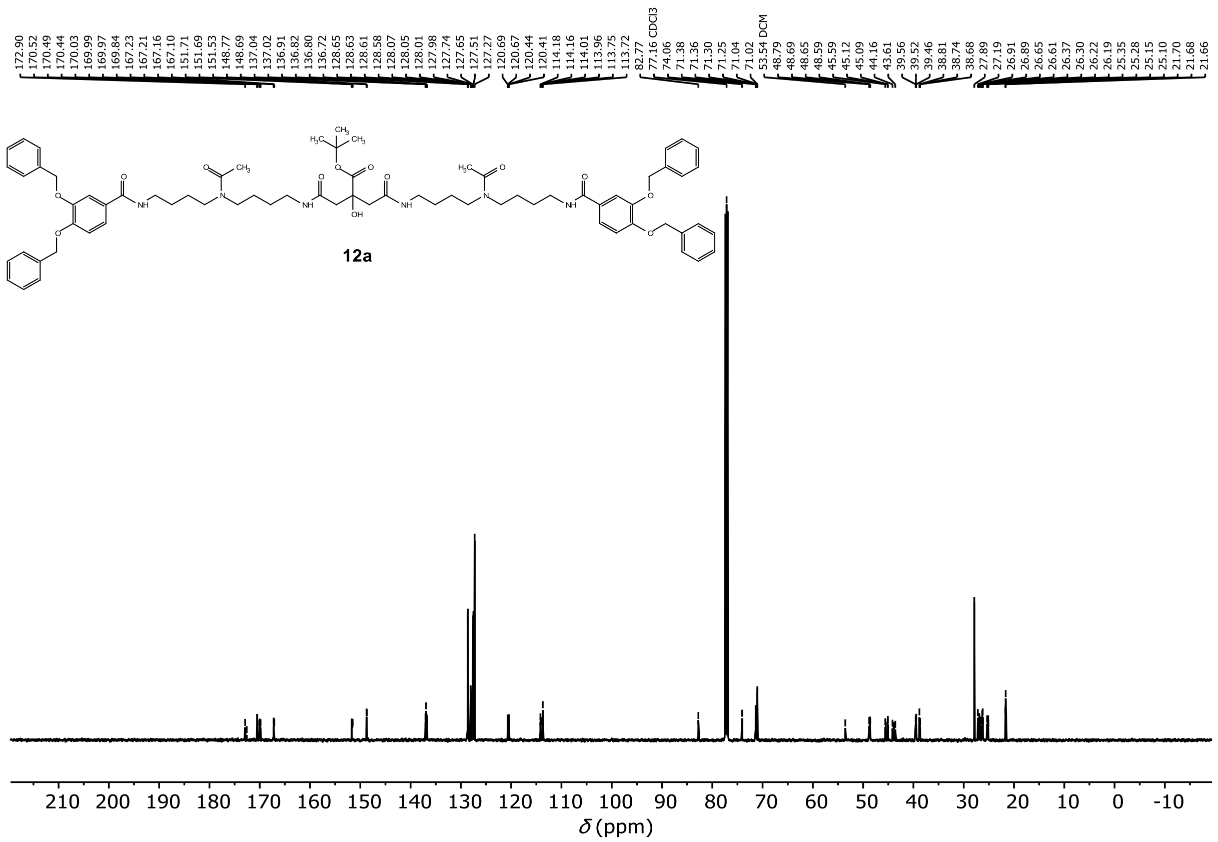

^{1}H NMR Spectrum of Compound **12b** in $CDCl_3$ (600 MHz)

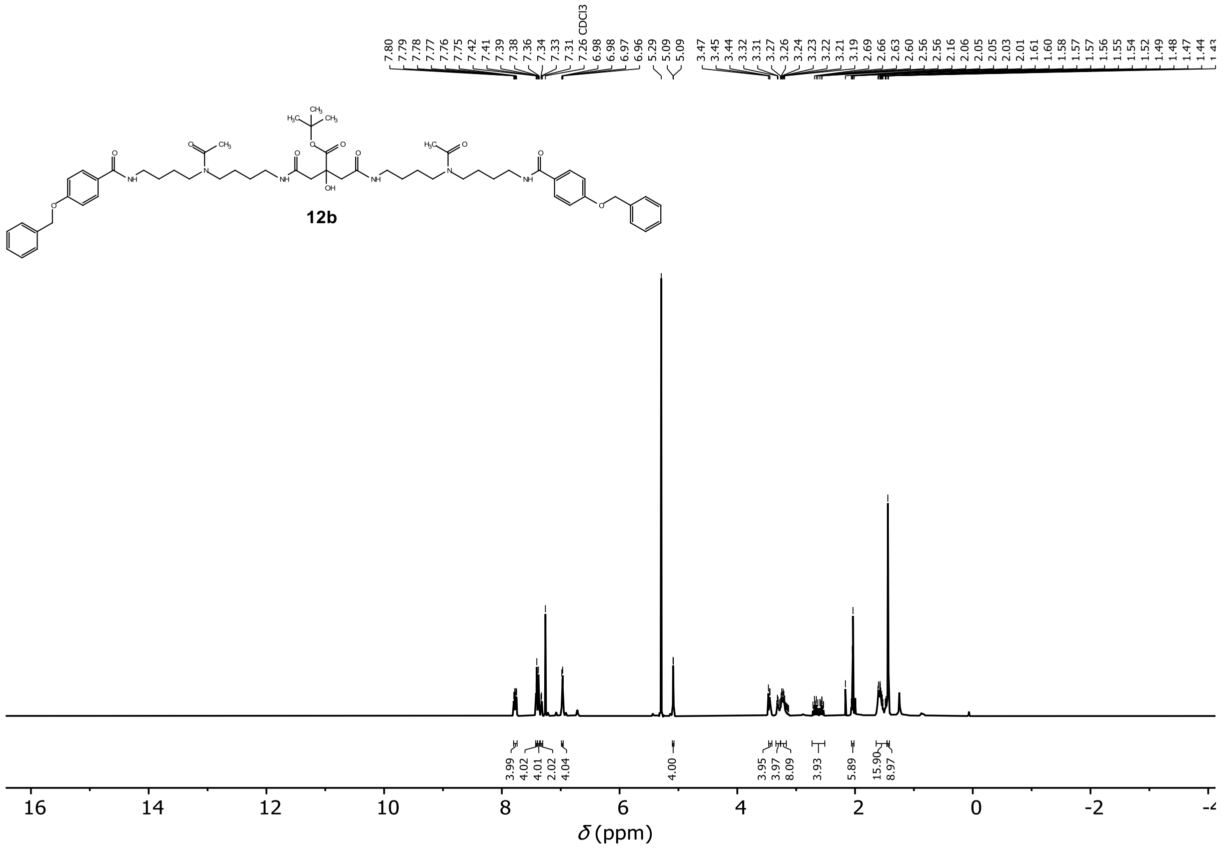

^{13}C NMR Spectrum of Compound **12b** in $CDCl_3$ (151 MHz)

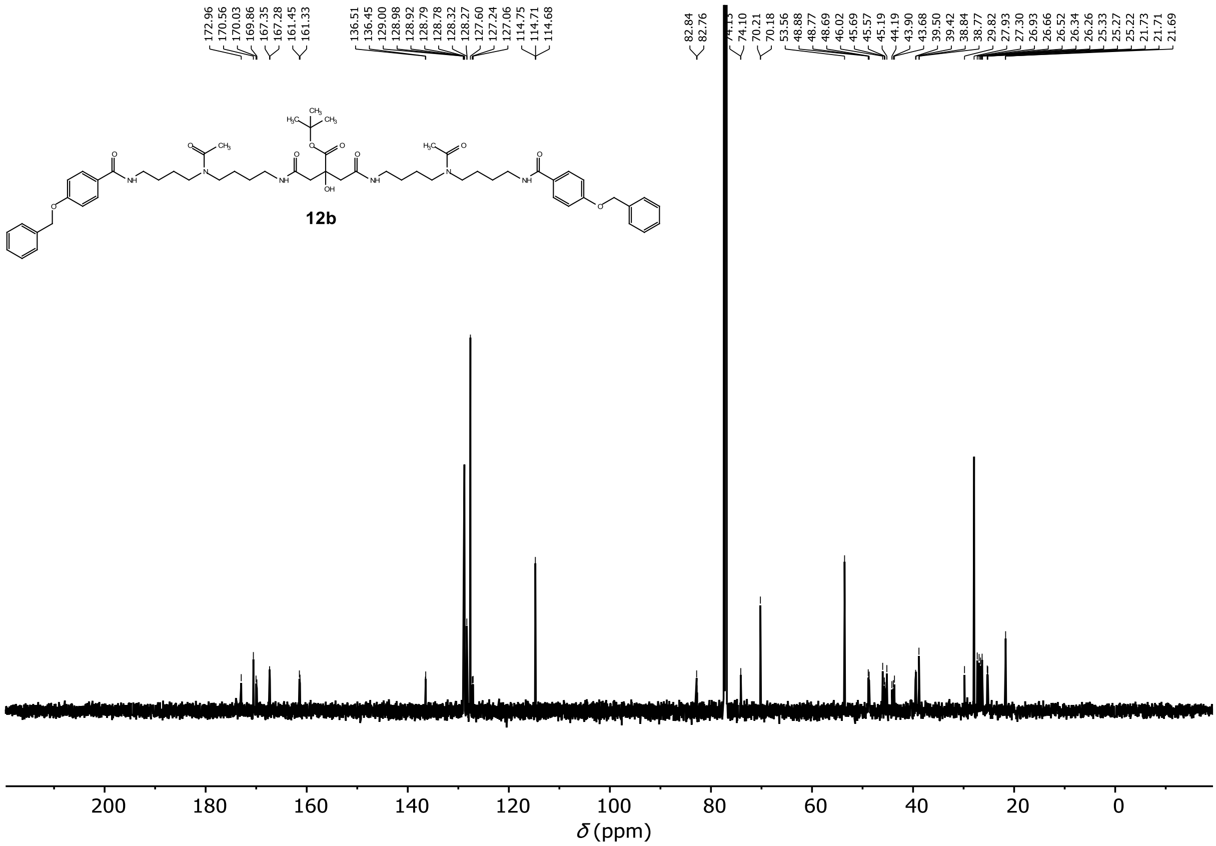

^{1}H NMR Spectrum of Compound **13a** in DMSO-d_6 (800 MHz)

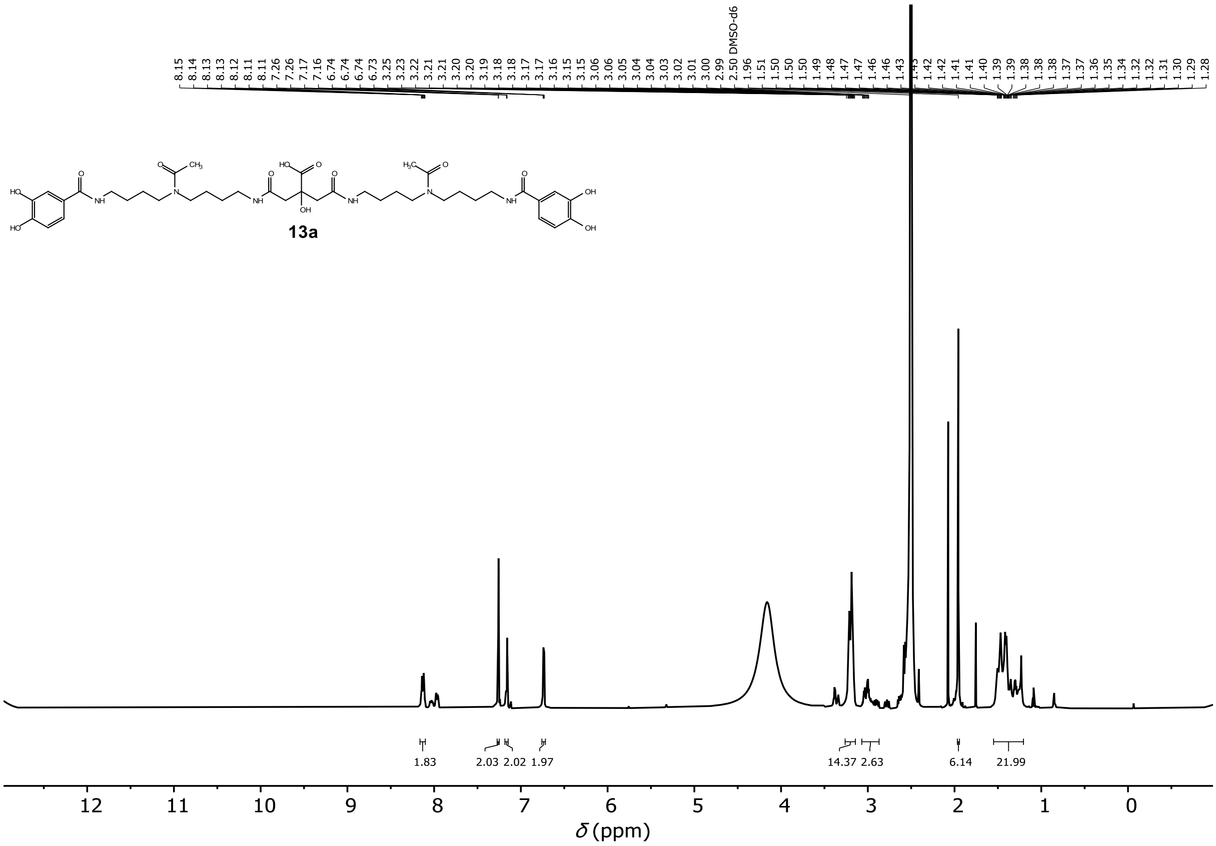

^{13}C NMR Spectrum of Compound **13a** in DMSO-d_6 (201 MHz)

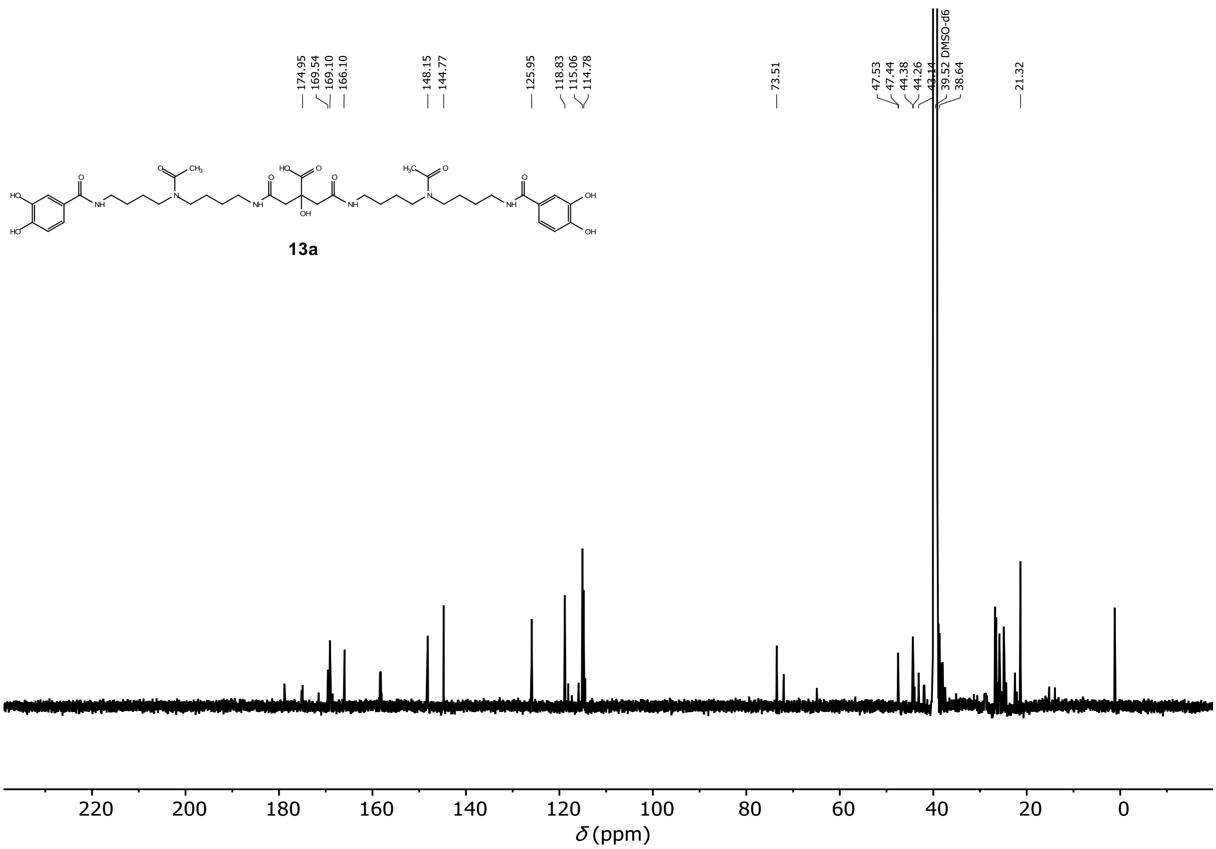

^{1}H NMR Spectrum of Compound **13a** in DMSO- d_6 (400 MHz)

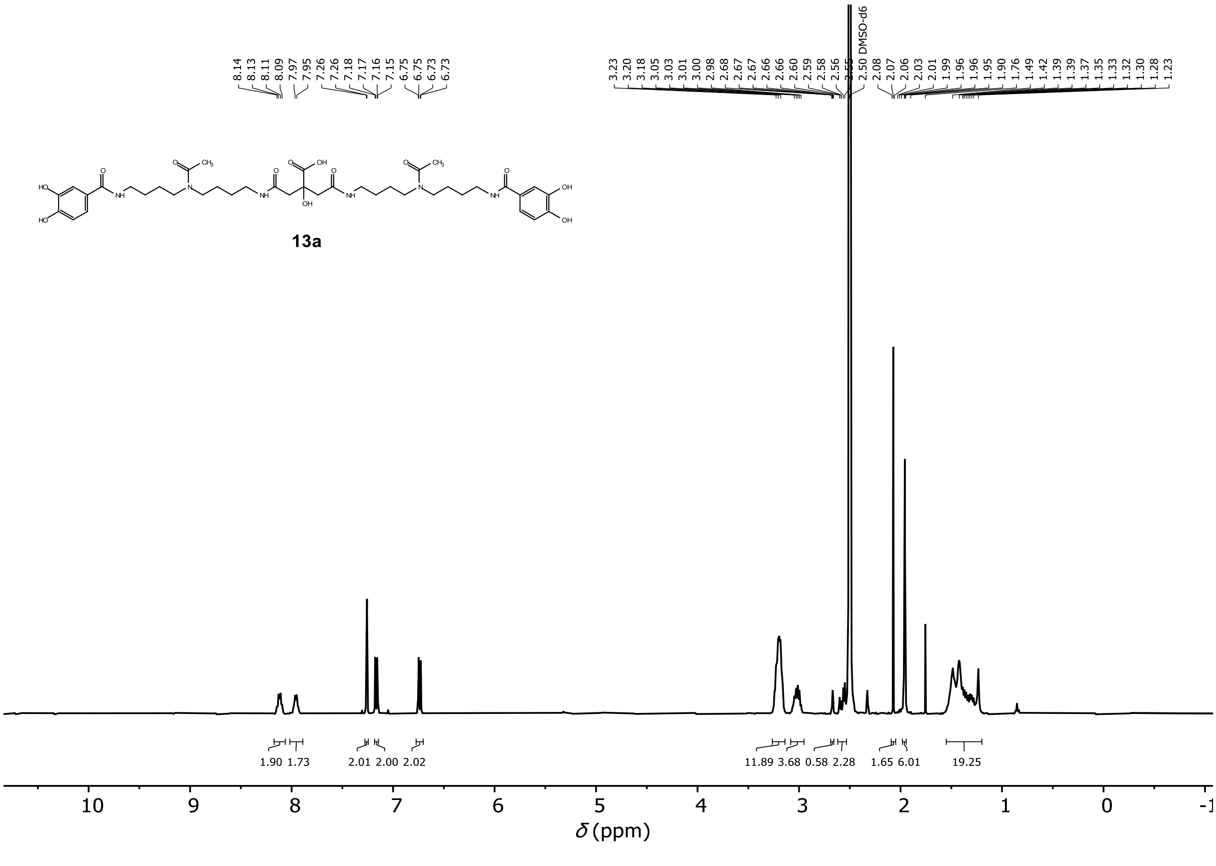

^{13}C NMR Spectrum of Compound **13a** in DMSO- d_6 (101 MHz)

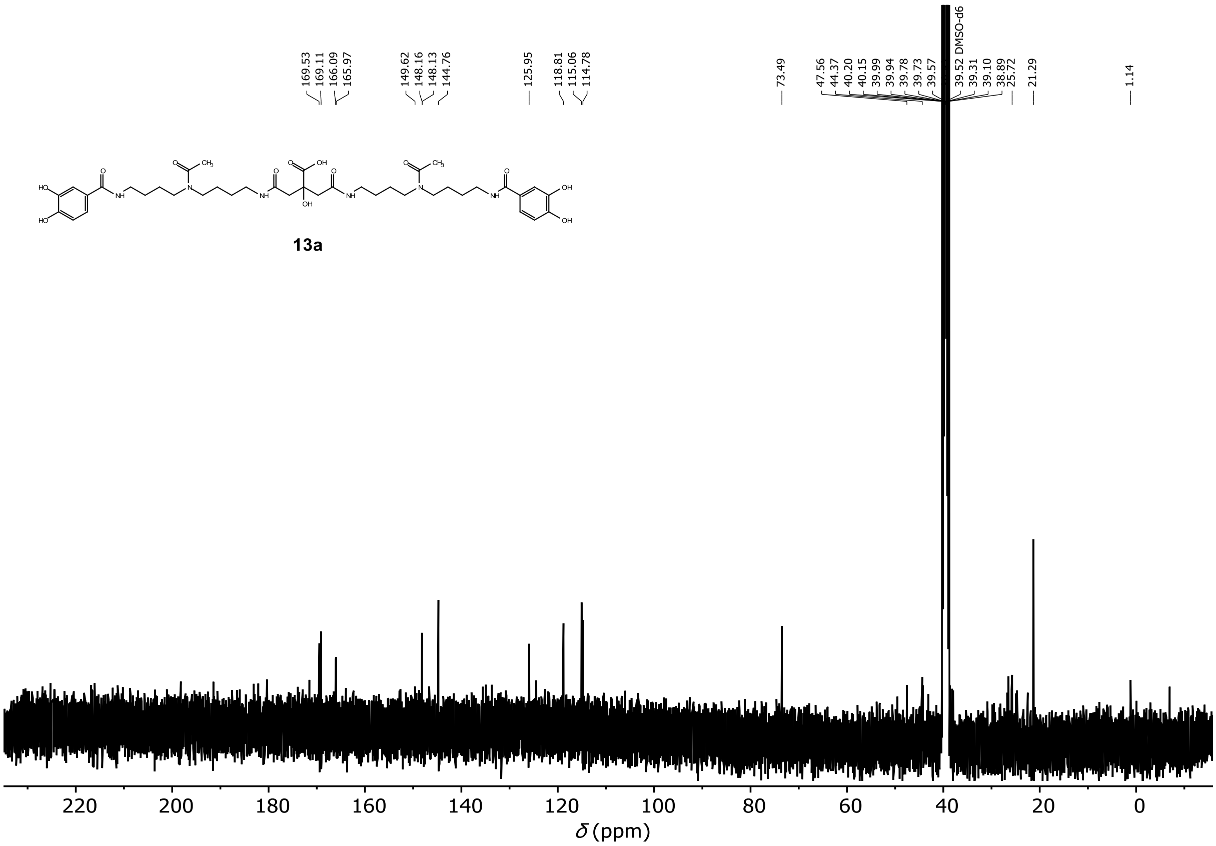

^{1}H NMR Spectrum of Compound **13b** in D_2O+0.003% TMSP-d_4 (800 MHz)

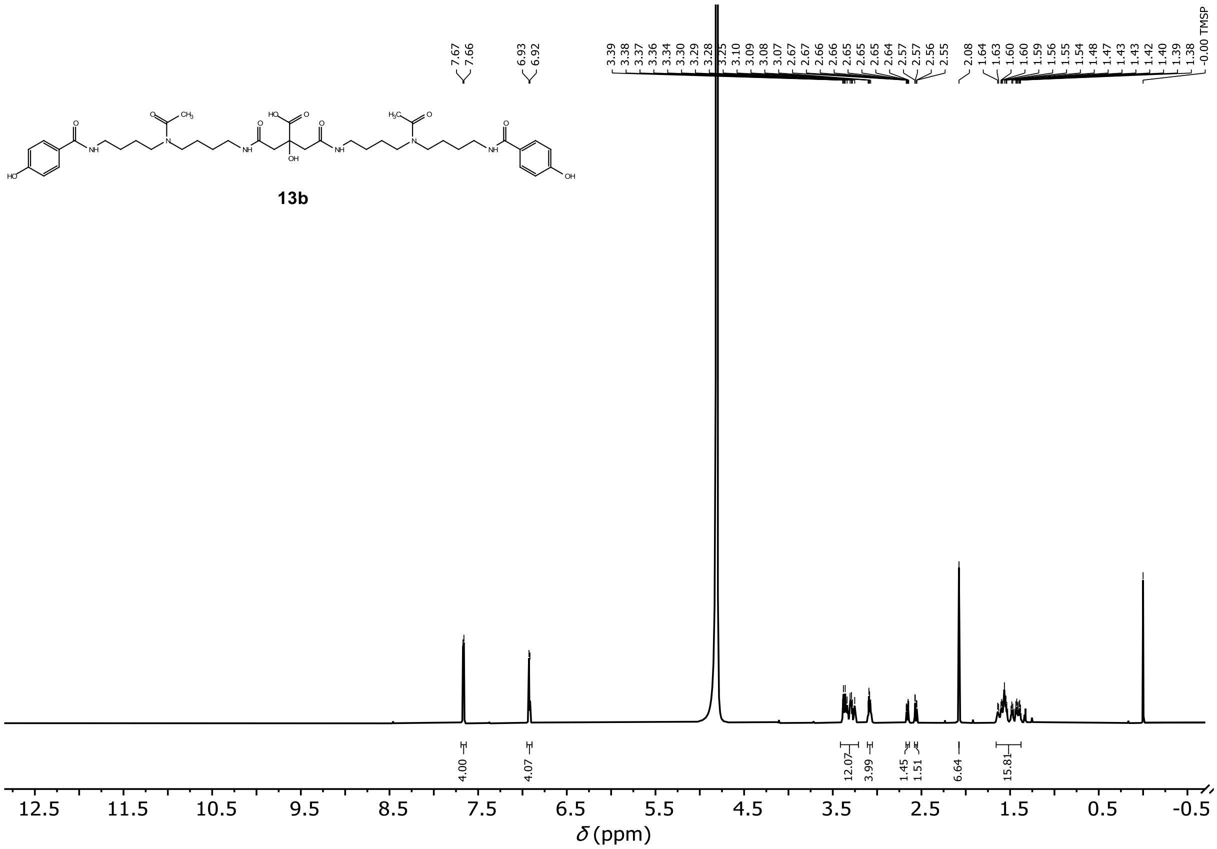

^{13}C NMR Spectrum of Compound **13b** in D_2O+0.003% TMSP-d_4 (201 MHz)

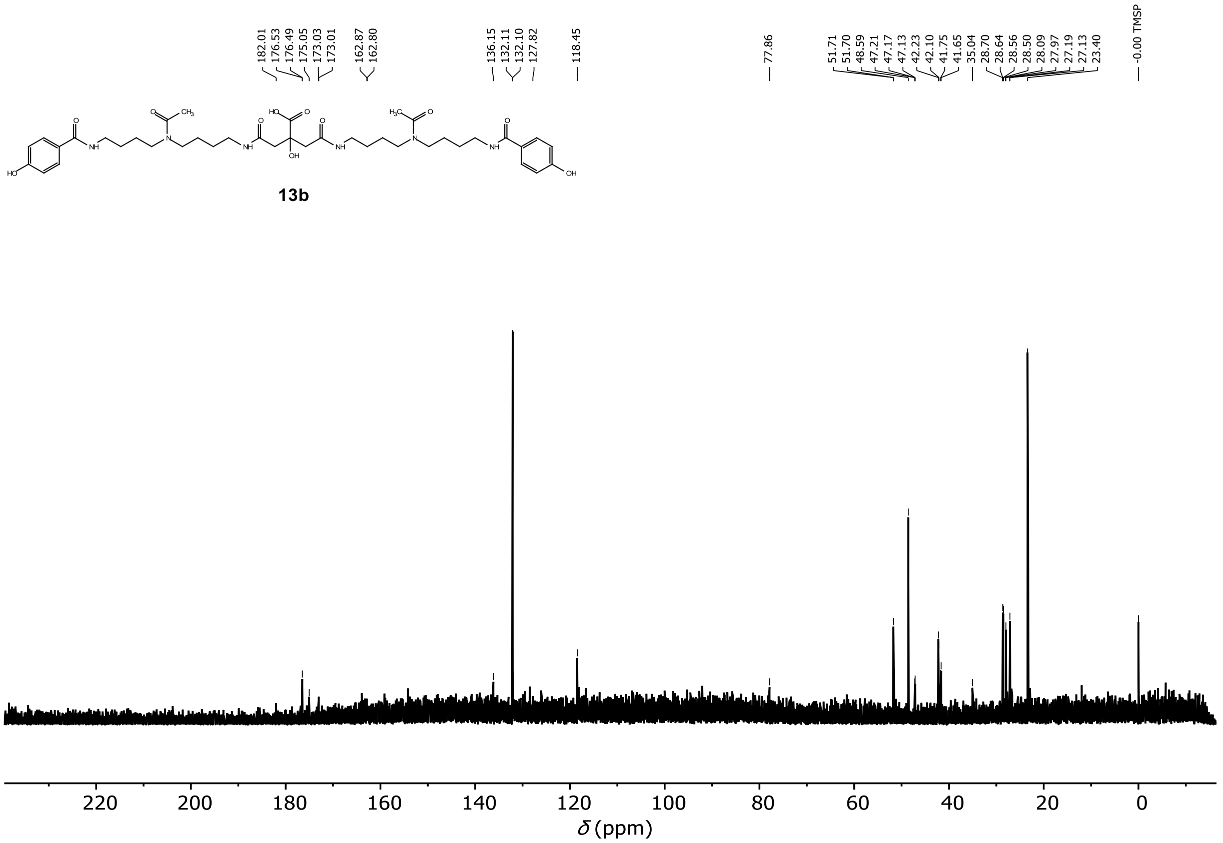

2 Binding Studies

2.1 General working methods

For all experiments ultrapure water (type 1, pH 5.6, 18.2 MΩ * cm at 25 °C) was used. Chemicals were obtained from commercial sources (*VWR, Merck KGaA, Sigma-Aldrich, abcr, ACROS Organics, Chempur, Deutero*) and used, unless stated otherwise, as supplied and handled according to potential hazards and toxicity. Metal salts for binding studies were used in trace metal basis (99.99% purity or greater).

Isolation and Purification of MLL from *Methylobacterium extorquens* AM1

MLL was purified from *M. extorquens* AM1 WT (wild-type) and *ΔmxaF* mutant with a procedure described in the literature.[4] The concentration of stock solutions in water was determined by UV-vis using the extinction coefficient from the literature.[4]

Preparation of RPB B stock solutions

The concentration of synthesized RPB B dissolved in water was determined by *q*NMR using a relaxation delay of 60 s as described elsewhere.[4] The used NMR spectrometer is described in section 2.3. The corresponding *q*NMR spectrum is shown in Figure S1.

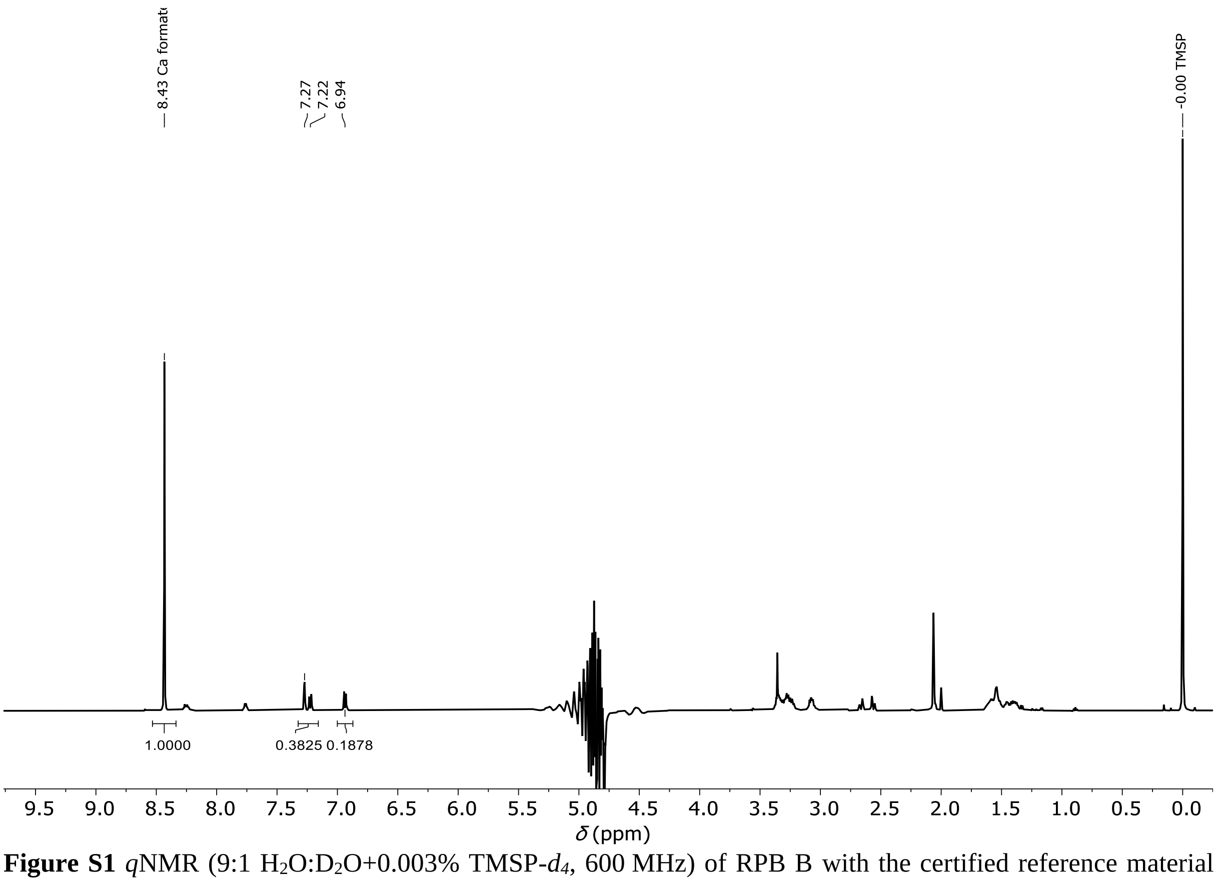

Figure S1 *q*NMR (9:1 H_2O:D_2O+0.003% TMSP-*d_4*, 600 MHz) of RPB B with the certified reference material calcium formate. The sum of the integrals of the aromatic signals was used for the quantification using a procedure described elsewhere.[4]

Metal stock solutions

$LnCl_3 \cdot n\ H_2O$ solutions were prepared as 10 mM stock solutions in water. $FeCl_3$ solutions were prepared as 10 mM stock solutions in 10 mM HCl. All stock solutions were stored at −20 °C until further usage.

2.2 UV-vis Spectroscopy

UV-vis spectroscopy was performed on an *Agilent* Cary 60 instrument with Peltier element at 25 °C. Spectra were recorded in a *Hellma* QS Ultra-Micro cell or in *Brand* UV micro cuvettes, both with 1 cm pathlength; all spectra were blank corrected with a spectrum of the used solvent. The scanning rate was 600 nm/min in 1 nm steps.

Lanthanide to Ligand Titration Experiments

Metal titration experiments were performed by adding a 1 mM $LnCl_3$ solution in increasing amounts over 5 steps (0 µM, 5 µM, 10 µM, 20 µM, 30 µM) to a buffered solution containing 10 µM MLL or RPB B, respectively. Both, the metal-containing titrant, as well as the ligand solution in the cuvette were prepared in buffer (10 mM MOPSO, 100 mM KCl, pH 6.0). Due to potential precipitation because of metal-addition the ligand was not added to the metal-titrant in order to keep the ligand concentration within the experiment constant. To keep the dilution negligible (start ligand concentration: 10 µM; end ligand concentration due to dilution effects: 9.7 µM) small volumes were added. After each titration step, the sample was mixed with a pipette and incubated for 10 min before the next measurement. For some titration series, the final titration step was centrifuged (21 000 × g, 15 min, 25 °C) and the obtained supernatant re-measured, for others the concentration of the final titration step was mixed directly by adding the volumes of all steps at once, followed by an incubation time of 10 min. Then the sample was measured and the supernatant re-measured after a centrifugation (21 000 × g, 15 min, 25 °C).

Chromeazurol S (CAS) Competition Experiment in Cuvettes

All stock solutions and solutions in general were prepared in plastic containers to avoid contamination with Fe. Metal-binding solutions containing 75 µM CAS and 200 µM hexadecyltrimethylammonium bromide (HDTMA) as well as 75 µM of the respective metal ($FeCl_3$ or $NdCl_3$) were mixed in water and incubated in the dark for 1 h at room temperature (r.t.). For Fe^{3+} it is important to use the stock solution in HCl directly without any prior dilutions in water to avoid decreasing Fe^{3+} concentrations due to precipitation as $Fe(OH)_3$. A mix without a metal as control was obtained analogously by only using CAS and HDTMA. Tested ligands were prepared as 75 µM stock solutions in water. The final samples were obtained by mixing

the incubated CAS/HDTMA solutions with buffer (stock: 50 mM MOPSO, 500 mM KCl, pH 6.0) and the respective ligand stock in water to yield the following final concentrations: 18.75 µM CAS, 75 µM metal and ligand; 50 µM HDTMA, 10 mM MOPSO, 100 mM KCl. After an incubation of 2 h in the dark and at r.t., spectra were recorded.

2.3 Metal to Ligand NMR Titration Experiments

NMR experiments were performed on a *Bruker* Avance III-600 14.1 T system corresponding to a 600 MHz ^{1}H frequency. Measurements were performed in a D_2O:H_2O mixture (1:9) + 0.003% TMSP-d_4 using water suppression (*zgesgp* pulse sequence). Metal salts were directly dissolved in the aforementioned mixture as 10 mM stocks and either used directly in this concentration or diluted 1:1 in the same solvent mixture. Titrations were either performed with $LaCl_3$, $La(NO_3)_3$ or $Ga(NO_3)_3$ in 5 steps (ligand to metal ratio: 1:0, 1:0.5, 1:1, 1:2 and 1:3). The ligand concentration was either 30 µM or 60 µM and the added volumes were kept minimal to keep the dilution during the titration neglectable (starting ligand concentration: 30 µM/60 µM, end ligand concentration due to dilution: 29.5/58.9 µM). Due to potential precipitation caused by metal-addition, the ligand was not added to the metal-titrant in order to keep the ligand concentration within the experiment constant.

2.4 Time-resolved laser-induced fluorescence spectroscopy (TRLFS)

Caution: When working with actinides special precautions must be taken. All samples containing actinides were handled in a radioactive controlled area following the official regulations.

Preparation of the Cm(III) (^{248}Cm (97.3%)/ ^{246}Cm (2.6%)/ ^{245}Cm (0.04%)/ ^{247}Cm (0.02%)/ ^{244}Cm (0.009%)) stock solution

A 7.3 mM stock solution of long-lived Cm isotopes in 1.0 M $HClO_4$ was converted to a 7 mM Cm(III) solution in 1 mM HCl (concentration determination *via* liquid scintillation counting) by adding 5 mM NaOH (1.5 mL) to the perchlorate solution (500 µL) until an alkaline pH was reached. After centrifugation (10 min, 13 000 rpm), the supernatant was discarded and the solid residue re-dissolved in 1 M HCl (500 µL). This process was repeated four times.

Used TRLFS Setups

Setup 1: Laser (*Spectra-Physics*, Quantaray Lab 170-20; *GWU-Lasertechnik Vertriebsgesellschaft mbH*, OPO: flexiScan); spectrometer (*Oriel Instruments*, MS257 Modell 77700A, grating: 600 line/mm) and ICCD camera (*Andor Technology*, iStar DH720-18H-13).

Setup 2: Laser (*Ekspla*, NT 230, ~5 ns pulse); spectrograph (*Andor Technology*, SR-303i-A) and ICCD camera (*Andor Technology*, i Star DH320T-18U-63).

For Eu^{3+} experiments the excitation wavelength was set to 394 nm and for Cm(III) experiments to 396 nm. Metal-titration experiments were performed at 25 °C in MOPSO buffer (10 mM, 100 mM KCl, pH 6.0 or pH 6.6) in stirred (1200 rpm) 4 mL or 2 mL *Hellma Analytics* quartz cells equipped with a screw cap and stirring bar.

Data Analysis

TRLFS data sets were analysed in MATLAB® using PARAFAC (N-way toolbox for MATLAB[5]) with modifications described in previous publications[6,7]. Raw data was loaded using the sif-reader.[8]

Ligand to Eu^{3+}-titration (1 µM Setup)

Used parameters: initial delay: 13 µs, linear step-size: 3+3x µs, width: 200 µs, slit width: 300 µm, 400 accumulations, kinetic series length: 21, gain: 4095.

MLL (20 µM titrant solution) was added stepwise over 20 additions (0-8.6 µM MLL) to a 1 µM $EuCl_3$ solution; the metal, buffer and salt concentration was kept constant during the experiment.

MLL to Cm^{3+}- titration

Used parameters: initial delay: 5 s, linear step-size: 0+1x µs, width: 200 µs, slit width: 300 µm, 200 accumulations, kinetic series length: 21, gain: 4095.

MLL (20 µM titrant solution) was added stepwise over 20 additions (0-8.5 µM MLL) to a 100 nM Cm^{3+} solution; the buffer and salt concentration was kept constant during the experiment. In order to facilitate the handling of radioactive samples, the titrant solution didn't contain any Cm^{3+} and thus the Cm^{3+} was diluted within the series. The dilution of the Cm^{3+} was taken into account during the data analysis.

Ligand to Eu^{3+} -titration (200 nM Setup)

Used parameters: initial delay: 10 or 5 µs, linear step-size: 3+3x µs, width: 400 or 1000 µs, slit width: 2000 µm, 400 accumulations, kinetic series length: 12 or 21, gain: 4095.

RPB B or MLL (40 µM titrant solution) was added stepwise over 20 additions (0-17.2 µM RPB B/ MLL) to a 200 nM $EuCl_3$ solution; the metal, buffer and salt concentration was kept constant during the experiment. After the last titration step the sample was centrifuged (10 min, 16 100 × g, r. t.) and the supernatant re-measured.

Competition experiment: MLL *vs.* nitrilotriacetic acid (NTA) with Eu^{3+}

Used parameters: initial delay: 15 µs, linear step-size: 3+3x µs, width: 200 µs, slit width: 300 µm, 200 accumulations, kinetic series length: 21, gain: 4095.

In three independent series (series 1-3), four buffered samples (10 mM MOPSO, 100 mM KCl, pH 6.0) were prepared containing 2 µM $EuCl_3$, 2 µM MLL and 0, 1, 2 or 4 µM NTA, respectively. Additionally, a control series (series 4) without MLL was mixed. After the mixing of MLL and $EuCl_3$, the samples were incubated at 25 °C for 24 h before NTA was added and the samples were incubated for another 24 h to ensure that thermodynamic equilibrium was reached. Due to inhomogeneities in the solutions containing Eu^{3+}-MLL complexes, direct spectroscopic evaluation was inconclusive (see Figure S2). To remove the particulate Eu^{3+}-MLL complex, the samples were centrifuged (20 000 × g, 20 min, r.t.), and the supernatant measured by TRLFS. At this point, no spectroscopically detectable Eu^{3+}-MLL signal remained in solution. Following the initial measurement, each sample was acidified by adding 1 µL concentrated HCl, which dissociated any remaining Eu^{3+} complexes and converted all Eu^{3+} to its aquo ion form. The variation in the aquo ion signal across the NTA concentration series provides thermodynamic information on the Eu^{3+}-MLL interaction, taking into account the well-characterised affinity of Eu^{3+} for NTA.[9] In total, the experiment comprises 35 samples, as listed in Table S2.

Table S2 Overview of the used sample numbers and corresponding samples.

Sample	Series	Subset	Description
1–8	Series 1	1–4	Series 1 acidified after centrifugation
		5–8	Series 1 centrifuged
9–16	Series 2	9–12	Series 2 acidified after centrifugation
		13–16	Series 2 centrifuged
17–24	Series 3	17–20	Series 3 acidified after centrifugation
		21–24	Series 3 centrifuged
25–32	Series 4 (Control)	25–28	Control Series without centrifugation
		29–32	Control Series acidified
33–35	Series 1–3	0 µM NTA samples	Samples without centrifugation

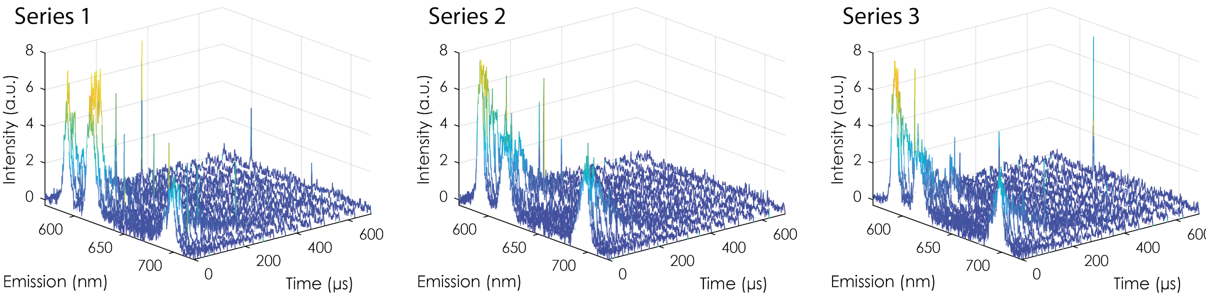

Figure S2 Matrix of the 0 µM NTA samples (Samples 33-35) series 1 to 3 before centrifugation with irregular scattering and high noise level due to the presence of precipitated MLL-Eu^{3+} complex. Therefore, the data of the samples prior to centrifugation could not be used for the analysis.

Thermodynamic Interpretation of the Eu^{3+}–MLL–NTA Competition:

As demonstrated in the literature,[7,10] PARAFAC combined with integrated speciation analysis is a powerful tool for the interpretation of TRLFS data. The PARAFAC results obtained from the sample set are shown in Figure S3.

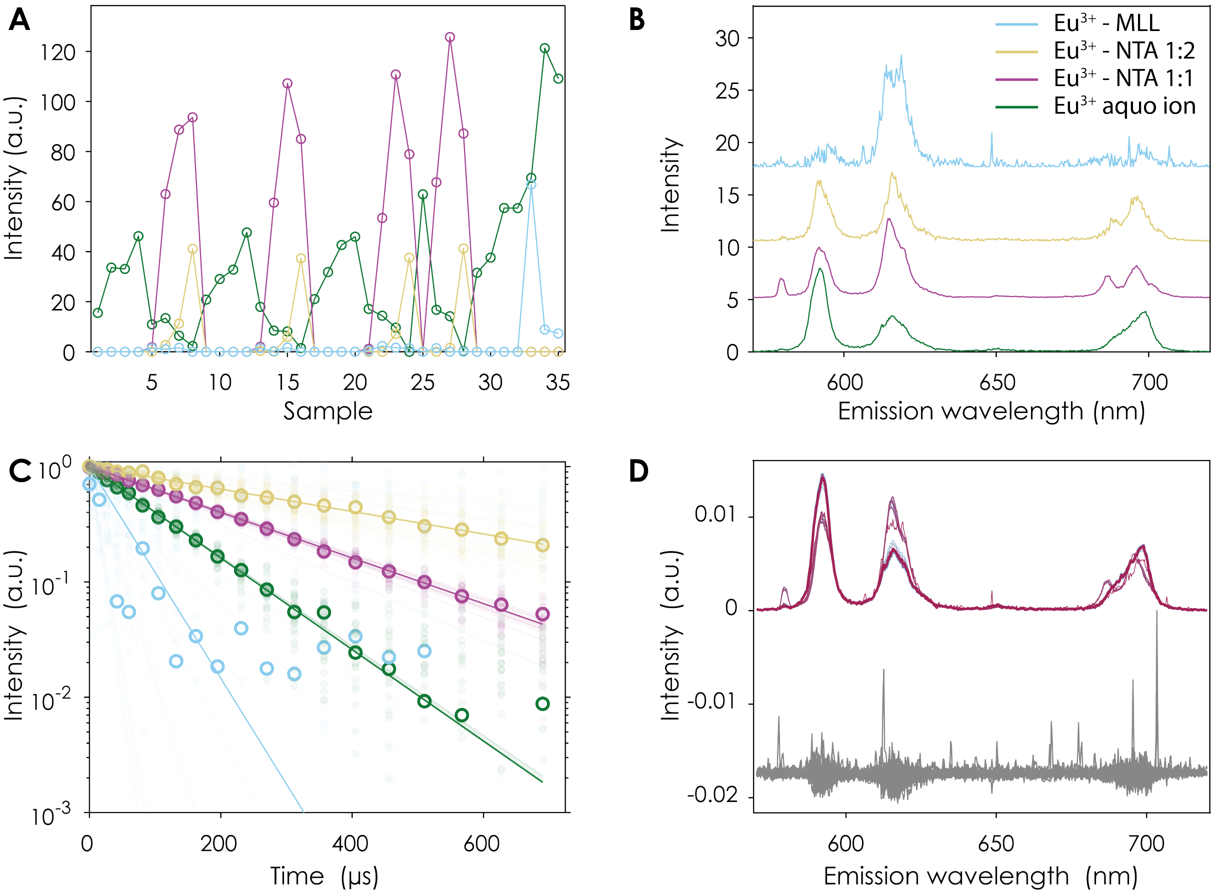

Figure S3 PARAFAC results of the sample set listed in Table S2. **(A)** Sample distribution corresponding to those listed in Table S2. It is clearly visible that no MLL complex can be detected in any of the samples—except for those that were not centrifuged (Samples 33–35). Furthermore, in the samples containing 4 µM NTA (Samples 4, 8, 12 ,16 ,20 ,24 ,28 ,32), a significant fraction of the 1:2 Eu^{3+}-NTA complex is present. These samples were therefore excluded from the speciation analysis. **(B)** Stacked emission spectra of the observed species within the sample set and **(C)** luminescence decay traces matching the expected patterns for the respective species. **(D)** Data versus noise: The residuals show low variance without any systematic deviations showing the robustness of the overall setup.

The Eu^{3+}-NTA system is well described in the literature.[9] The 1:1 complex has a formation constant of log β = 11.45, which corresponds to an affinity of log K_a = 7.9 (pH 6.0, I = 100 mM KCl). With increasing NTA concentration, this ligand competes with MLL for Eu^{3+} and effectively removes Eu^{3+} from the Eu^{3+}-MLL complex. Since the Eu^{3+}-MLL complex precipitates and is removed by centrifugation, higher NTA concentrations result in more europium remaining in solution. The signal observed after acidification therefore reflects both the europium that was initially present as free ion and the fraction that was complexed with NTA. The NTA-dependent trend of this signal can be modeled using a five-component speciation system, consisting of: Eu^{3+}-aquo ion uncomplexed MLL, uncomplexed NTA, 1:1 Eu^{3+}-MLL and 1:1 Eu^{3+}-NTA. As foundation for the speciation analysis workflow in MATLAB the work of Smith was used.[11] The representing stoichiometry matrices were defined as follows:

For the components in solution

Eu^{3+}	MLL^{3-}	NTA^{3-}
1	0	0
0	1	0
0	0	1
1	0	0

For the solid component (Eu^{3+}-MLL)

Eu^{3+}	MLL^{3-}	NTA^{3-}
1	1	0

This reaction network has the uncomplexed components (Eu^{3+}, MLL^{3-}, NTA^{3-}) as basis species which form neutral 1:1 complexes according to

(1) K_a $Eu^{3+} + NTA \rightleftharpoons Eu^{3+}\text{-}NTA$

(2) K_{solid} $Eu^{3+} + MLL \rightleftharpoons Eu^{3+}\text{-}MLL$

Instead of the formation constant of a solid phase, its reciprocal is usually given, referring to the dissolution process.

(3) $K_{sp} = 1/K_{solid}$ $Eu^{3+} + MLL \rightleftharpoons Eu^{3+}\text{-}MLL$

The 1:2 Eu^{3+}–NTA complex is not included in the model, as the samples with 4 μM NTA – where this species is significant – were excluded from the analysis. The first three samples of series 1 to 3 were analyzed globally using this model. The corresponding speciation is shown in Figure S4.

Using this speciation model as the chemical basis, the first three data points of the acidified samples from each series (samples 1–3, 9–11, and 17–19) can be fitted as the sum of the Eu^{3+}-aquo ion and the 1:1 Eu^{3+}-NTA complex, as shown in Figure S4A. Since the solubility product K_{sp} of the Eu^{3+}-MLL complex is the only unknown parameter in this system, it can be fitted directly to best reproduce the PARAFAC-derived data points (Figure S4B).

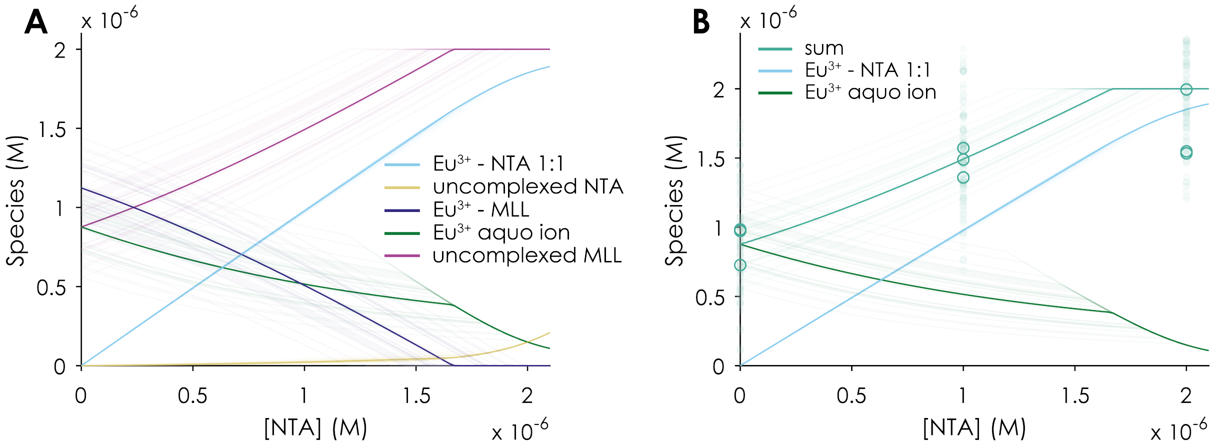

Figure S4 Analysis of the competition experiment with respect to the thermodynamic parameters of the Eu–MLL system. (A) The speciation for the investigated system comprises three different Eu^{3+} species and two uncomplexed ligands. In this system the solubility product of the Eu^{3+}-MLL complex is the only unknown parameter. Adjustment of this parameter therefore affects the entire speciation. The ligands themselves do not produce any fluorescence signal, and the Eu^{3+}–MLL complex is removed from the solution by centrifugation. (B) The sum of the two remaining species can be fitted to the PARAFAC and MC data (solid and transparent symbols), corrected for fluorescence efficiency.

Europium Species in ½ Hypho Medium

Used parameters: initial delay: 5 µs, linear step-size: 3+3x µs, slit width: 200 µm, kinetic series length: 21, gain: 4095.

Samples were mixed from the stock solutions (Table S3) usually used to prepare ½ Hypho medium based on the literature-known Hypho medium[12] with modifications described elsewhere.[4] In total five (4 mL) samples were prepared, always with the final concentration of the respective media component and 2 µM $EuCl_3$. Sample 1: only contained the P-Solution; Sample 2: P- and S-Solution, Sample 3: P- and S-Solution and Trace A, Sample 4: P- and S-Solution and Trace A+B, Sample 7: P- and S-Solution and Trace A+B, 125 mM MeOH. In comparison, autoclaved ½ Hypho medium containing 2 µM $EuCl_3$ and 125 mM MeOH (Sample 8) as well as an aqueous 2 µM $EuCl_3$ solution (Sample 9) were measured. The samples are summarised in Table S4 and the data and PARAFAC results are shown in Figure S5.

Table S3 Overview of the stock solutions used to prepare ½ Hypho medium as well as the final concentration in the mixed full medium.

Stock solution	Final Concentration in ½ Hypho medium
P-Solution (100X)	7.25 mM K_2PO_4, 9.4 mM NaH_2PO_4
S-Solution (50X)	0.8 mM $MgSO_4$, 3.8 mM $(NH_4)_2SO_4$
Trace A (1000X)	3.59 µM $FeSO_4$, Na_2EDTA 26.86 µM
Trace B (1000X)	9.98 µM $CaCl_2$, 5.11 µM $MnCl_2$, 0.18 µM $(NH_4)_6Mo_7O_{24}$, 1.26 µM $CuSO_4$, 6.74 µM $CoCl_2$, 15.3 µM $ZnSO_4$

Table S4 Overview of the investigated samples and their composition corresponding to the results shown in Figure S5.

Sample Number	Sample composition; always including 2 µM $EuCl_3$
1	P-Solution
2	P-Solution, S-Solution
3	P-Solution, S-Solution, Trace A
4	Sample 3 + 10 mM HCl (10 µL)
5	Sample 3 + 10 mM HCl (20 µL)
6	P-Solution, S-Solution, Trace A+B
7	P-Solution, S-Solution, Trace A+B,125 mM MeOH
8	Pre-mixed and autoclaved ½ Hypho
9	Basis spectrum Eu-aquo ion

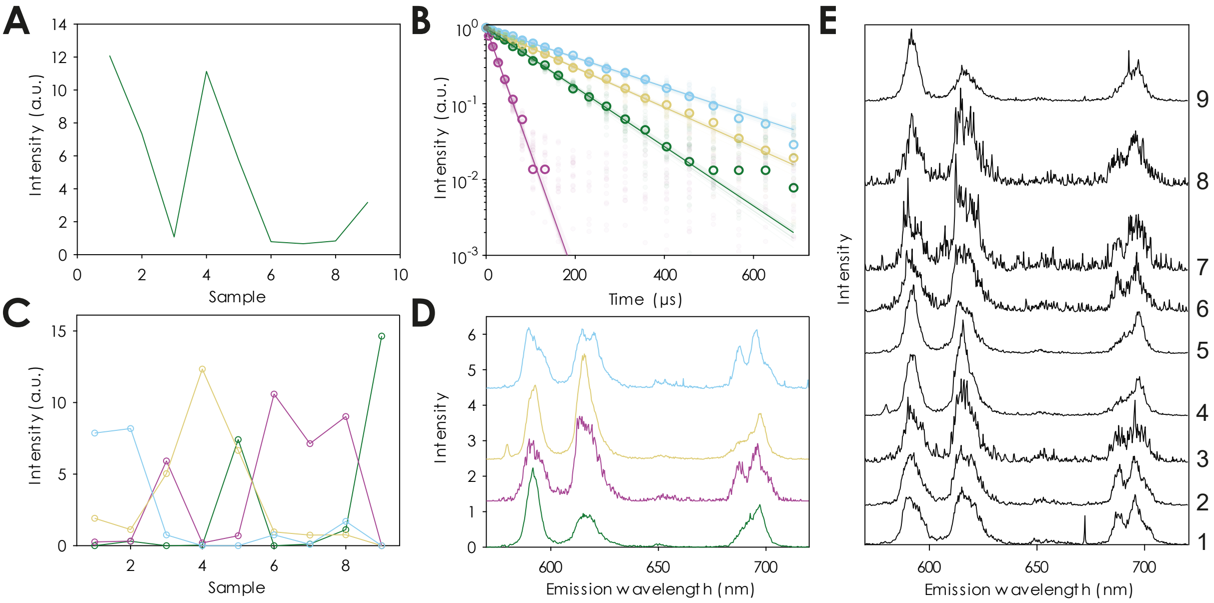

Figure S5 TRLFS measurements of 2 µM $EuCl_3$ in ½ Hypho medium (see Table S3) testing the different media components. Table S4 gives an overview on the sample numbers and corresponding composition that are reflected in the intensity distribution (A) and species distribution (C). **(A)** Intensity distribution of the tested samples with significant variations depending on sample composition, partly attributable to Fe-induced quenching. The data set was normalized prior the PARAFAC analysis to ensure equal weighting. **(B)** Luminescence decay curves of the observed species. **(C)** PARAFAC species distribution. **(D)** Emission spectra of the resolved species. The green spectrum is assigned to the Eu^{3+}-aquo ion (110.8±2.8 µs), and the blue spectrum to inorganic phosphate (223.5±5.3 µs).[13] The magenta spectrum (26.5±0.7 µs) shows nearly identical spectral features to the blue phosphate species, but with a significant shorter luminescence lifetime and lower intensity in the presence of Fe. This deviation from trilinearity requires a separate factor in the PARAFAC analysis, even though both spectra represent the same underlying Eu^{3+}-phosphate species. The yellow species, which predominates after acidification of the sample, is assigned to a EDTA-Eu^{3+}-like species (166.5±3.4 µs).[9] **(E)** Spectra of volume normalised emission t_0 emission spectra of all nine sample (the corresponding sample number is given right to the respective spectrum). Normalisation was performed with respect to the individual TRLFS matrix volume.

Comparison of Availability of $EuCl_3$ and Eu_2O_3 in ½ Hypho medium

Used parameters: initial delay: 5 µs, linear step-size: 3+3x µs, slit width: 600 µm, 400 accumulations, kinetic series length: 12, gain: 4095.

Autoclaved ½ Hypho medium was spiked with either 2 µM $EuCl_3$ or 1 µM Eu_2O_3 and incubated overnight at 30 °C. Samples were measured directly after vortexing the samples in a stirred cuvette (1200 rpm). Then the samples were centrifuged (10 min, 16 100 × g, r.t.) and the supernatant measured using the same settings.

2.5 Chemical Microscopy

Chemical Microscopy was performed according to the literature[13] on a *Horiba* LabRAM Raman microscope using a 532 nm external Nd-YAG laser as light source with an output energy of 50 mW which was coupled to an *Olympus* BX-40 microscopy (10-fold, 50-fold or 100-fold magnification). Both, Eu^{3+} luminescence and Raman signals were captured with the same objective and guided through a pinhole (200 µm) to a spectrometer (300 l mm^{-1} grating; 200 µm entrance slit width) before reaching a Peltier cooled CCD (−70 °C). Data acquisition

was performed between 560 nm and 725 nm using the *Horiba* LabSpec 5 software. Samples were obtained after centrifugation and put on a slide and covered with a coverslip. Datasets were background corrected and deconvoluted; the spectra of the factors and distributions were restricted to being non-negative (non-negative iterative factor analysis (NIFA)). For more details for the data treatment and analysis see literature.[13]

2.6 Cyclic Ion Mobility Spectrometry-Mass Spectrometry (cIMS-MS)

Samples were prepared by mixing an aqueous solution of MLL (1 µM, 30 µL) with an aqueous solution of the respective metal (100 µM, 30 µL) which was then further diluted with 100 µL MeOH. Arrival time distributions (ATD) were measured in a *Waters* Select Series Cyclic instrument. The instrument comprises an electrospray ion source, a quadrupole mass filter, a cIMS cell, and a high-resolution time-of-flight mass spectrometer. In the IMS cell the ion packet is guided by an oscillating electrical field („travelling wave") through 1.7 mbar of N_2 as drift gas. The wave parameters used for $[MLL{-}H + M]^{2+}$ and $[RPB\ B{-}H{+}M]^{2+}$ were: wave height 22 V and wave speed 375 m/s. Either 20 or 50 cycles were measured. The corresponding collision cross sections (CCS) are usually termed $^{TW}CCS_{N2}$ since the values depend on the drift gas (N_2) and (slightly) on the method (TW = travelling wave) used to determine the arrival time distribution. The measured arrival times consist of the drift time of the ions in the IMS cell and the time the ions spend outside the cell prior to detection. In order to eliminate these end effects and to get highly accurate $^{TW}CCS_{N2}$ we measured ATDs after 1, 5, 10, 15 and 20 cycles and determined the peak position by fitting a gaussian to the distributions. Next, we plotted the arrival time peaks against the number of cycles and determined the time-per-cycle by a linear fit. Finally, we repeated the same procedure with a set of calibrant ions („agilent tune mix" (*Agilent*, G1969-8500)) with known CCS.[14] With this procedure we obtain highly reproducible $^{TW}CCS_{N2}$ with statistical errors well below 0.5%. Since the series of all tested metals was measured within one run, the relative errors are below 0.25%. This is sufficient to detect the lanthanide contraction in the complexes, *i.e.* the small CCS decrease from ca. 307 Å^2 (La) to 302-303 Å^2 (Lu) *i.e.* a relative difference of 1.2-1.5 % due to the decreasing size of the metal centres (Table S5).

3 Investigation of Nd Cell Accumulation and MLL Excretion

3.1 General Working Methods

For all experiments ultrapure water (type 1, pH 5.6, 18.2 MΩ * cm at 25 °C) was used. Chemicals were obtained from commercial sources (*Sigma-Aldrich, Carl-Roth, Chempur*) and used as supplied unless stated otherwise and handled according to potential hazards and toxicity. For bacterial cultivation chemicals suitable for microbiology were used and consumables were either purchased sterile or autoclaved prior to use. For autoclaving a *VWR* VAPOUR-Lineeco 25 or a *Systec* VX75 autoclave was used. For liquid chromatography coupled to mass spectrometry (LC-MS) analysis solvents in LC-MS purity were used.

3.2 Cultivation in 48-well plates

Cultivations of *M. extorquens* AM1 WT (wild-type) and *M. extorquens* AM1 Δ*mxaF* were started from a frozen stock by streaking out on MP+S (MP medium[12] + 15 mM succinate) agar plates and subsequent incubation for 4-7 days at 29 °C. Overnight cultures (3 mL) were grown in MP+S (MP medium[12] + 15 mM succinate) medium in 14 mL *Greiner* culture tubes with vented caps after inoculation with 5-7 single colonies (17-22 h, 29 °C, 250 rpm). The cells were separated by centrifugation (2 min, 3000 × g, 25 °C) and washed (3 × 2mL) with ½ Hypho medium described elsewhere[4] (Table S2) which was prepared without $FeSO_4$ and supplemented with 50 mM MeOH (LC-MS grade). After washing, the cells were resuspended in 1 mL Fe-limiting medium and used to inoculate Fe-replete ½ Hypho medium as well as Fe-limiting ½ Hypho medium with an optical density at 600 nm (OD) of 0.1 (*Brand* PS semi-micro cuvettes; 10 mm pathlength; *Fisher Scientific* Fisherbrand cell density meter). Growth experiments were then performed in 48-well plates (*Corning*, Falcon) in 625 µL per well consisting of 600 µL of the respective cell suspension (+/−Fe) and 25 µL of a 50 µM $NdCl_3$ solution for a final Nd concentration of 2 µM or autoclaved water (−Ln conditions). Growth experiments were performed in an *Agilent* epoch 2 microplate spectrophotometer: 29 °C, orbital shaking (548 rcf), readings at 600 nm every hour. Cells were harvested in the early stationary phase. To obtain enough supernatant/cells for samples for LC-MS and scICP-MS four wells of the same condition were pooled to yield one sample. The obtained samples were centrifuged (10 min, 7000 × g, 25 °C), the supernatant syringe filtered (0.2 µm, PES) and stored at −20°C upon sample preparation for LC-MS measurements and the cells fixed for scICP-MS.

3.3 Cell Fixation with Glutaraldehyde

Cells were fixed overnight at 4 °C (300 µL, 2.5% glutaraldehyde in 100 mM cacodylate buffer, pH 7.3). Then the cell suspension was centrifuged (10 min, 7000 × g, 25 °C), the supernatant discarded and the cells washed with buffer (3 × 600 µL, 100 mM cacodylate, pH 7.3); between the washing steps the cells were incubated 5, 10 and 15 min. After the final washing step, the cell pellet was resuspended in 300 µL buffer (100 mM cacodylate, pH 7.3) and the cell number estimated using a counting chamber.

3.4 Sample Preparation and LC-MS analysis

Supernatant samples (1.5 mL) were extracted using HLB solid-phase extraction (SPE) columns (*Macherey-Nagel* Chromabond, 60 µm, 30 mg). The columns were conditioned with MeOH (2 × 1 mL) and equilibrated with water (2 × 1 mL) before the sample was applied. The column was washed with water (1 × 1.5 mL), dried under reduced pressure and eluted in rinsed 15 mL centrifugation tubes (rinsed with water and MeOH and dried under reduced pressure before use) with MeOH (2 × 1 mL). MeOH was removed using a SpeedVac (*Thermo Fisher Scientific*, SPD 120, 35 °C, 1.5 h) and residual water by lyophilisation.

Samples were dissolved in water:MeCN (1:1, 150 µL) and syringe filtered (0.2 µm, PTFE). A calibration curve of MLL with samples between 0 µM and 2.5 µM was prepared. Of all samples 8 µL were injected into an *Agilent* QTOF 6530 C coupled to an *Agilent* HPLC 1260 Infinity II instrument (G7115A 1260 DAD WR, G7116A 1260 MCI, G7167A 1260 multisampler, G7104C 1260 flexible pump) equipped with an *Agilent* Poroshell 120 EC-C18 column (3.0 × 150 mm, 2.7 µm). Column separation was performed at 30 °C with a flowrate of 0.7 mL/min using water + 0.1% FA (solvent A) and MeCN + 0.1% FA (solvent B) as a mobile phase. The following method was used: 2 min isocratic at 98:2% (A:B), followed by a gradient from 2% B to 98% B in 18 min, followed by a 6-min washing step at 98% B and 3 min re-equilibration. The retention time of MLL was 8.9 min. MS measurements were performed in positive mode (extended dynamic range *m/z* 1700) using the following settings: Gas Temp: 250 °C, Drying Gas: 11 L/min, Nebuliser 45 psi, Sheath Gas Temp 350 °C, Sheath Gas Flow 12 L/min; VCap 3500 V, Fragmentor 100 V, Skimmer 65 V, Oct 1 RF Vpp 750 V; mass range: 100-1700; rate 5 spectra/s; time 200 ms/spectrum; transients/spectrum 2641; Used reference masses: 121.0509 and 922.0098.

For the quantification of MLL the extracted ion chromatogram (EIC) of $[MLL+H]^+$ (*m/z* 799.4236) was used (+/−20 ppm). The EICs of samples with known MLL concentration (calibration samples) were integrated (integrator: Chemstation) and the area under the peak

plotted against the concentration and fitted using the following equation: $y = ax+bx^2$. After obtaining a and b the equation was used to determine the MLL concentration in the measured samples which were extracted and integrated analogously to the calibration samples. SPE samples were 10-times more concentrated than the original supernatant samples, thus the determined concentration was divided by 10. Then, the obtained concentration was divided by the average OD of the pooled wells in order to correct for different cell densities. Mass spectrometric data were analysed using *Agilent* MassHunter 10.0, and further evaluation and statistical analyses were performed with *OriginPro* 2021b.

3.5 scICP-MS

scICP-MS analyses were conducted on an *Analytik Jena* single-quadrupole ICP-MS PlasmaQuant Elite MS equipped with Ni sampler and skimmer cones. A specialised cell introduction kit consisting of a *KD Scientific* syringe pump and a combination of low-volume nebulizer and total-consumption-on-axis spray chamber (CytoNeb/CytoSpray) from *Elemental Scientific* was used. Sample flow rate was set to 10 µL/min. RF power was at 1500 W, sheath and nebulizer gas flow rates were set to 0.93 L/min and 0.20 L/min, respectively. The isotopes ^{146}Nd and ^{56}Fe were monitored consecutively at a dwell time of 100 µs each for a total of 180 s. For calibration, aqueous ionic standards containing 0 ng/mL to 100 ng/mL Nd or Fe were prepared from the respective NISTcertified ICP-MS standard solution (TraceCert®; *Sigma-Aldrich*). Transport efficiency was determined using Au nanoparticles (*nanoComposix,* 50 nm citrate-coated NanoXact) *via* the particle frequency method proposed by Pace *et al.*[15] Cell samples were diluted to approximately 5.0×10^5 cells/mL using water and immediately analysed. As for the Δ*mxaF* samples, similar to the WT samples, no significant differences between the event intensities of the two different sample conditions (+/−Fe) were observed, thus the Fe quantification in fg/cell was omitted. Data analysis was carried out using the in-house developed software spTool2. A detailed description of the event detection algorithm can be found elsewhere.[16] Masses per cell were calculated from event signal intensities and visualised using *OriginPro* 2021b and *Microsoft* Excel version 2108.

4 Quantum Chemical Calculations

Candidate structures of $[Ligand\text{-}H+La]^{2+}$ and $[Ligand+2H]^{2+}$ ions were obtained in two steps: First, the conformational space using the CREST package,[17] which is based on the semiempirical XTB program[18] was explored. Then reasonable structures were optimised with the DFT method as implemented in the TURBOMOLE package.[19,20] All calculations were performed with the def-SV(P) basis set. We tested three different functionals: BP-86[21–23] B3-

LYP[21,23–25] and TPSS.[26,27] The geometries and relative energies are very similar, therefore we focused on the BP-86 results. Based on the DFT-optimised structures we performed trajectory method (TM) calculations with the IMoS package.[28,29] The calculations simulate a large number of interactions between nitrogen molecules and the drifting ion. By integration of the scattering angles a theoretical CCS is obtained that can be directly compared with the experimental value in order to confirm or rule out a candidate structure. The simulations are based on Lennard-Jones type and ion-induced dipole interactions between ion and N_2. The partial atomic charges are obtained by Mulliken population analysis. Based on the approximations within this model, deviations between experiments of 2-5% are common.[30]

5 Supplementary Tables and Figures

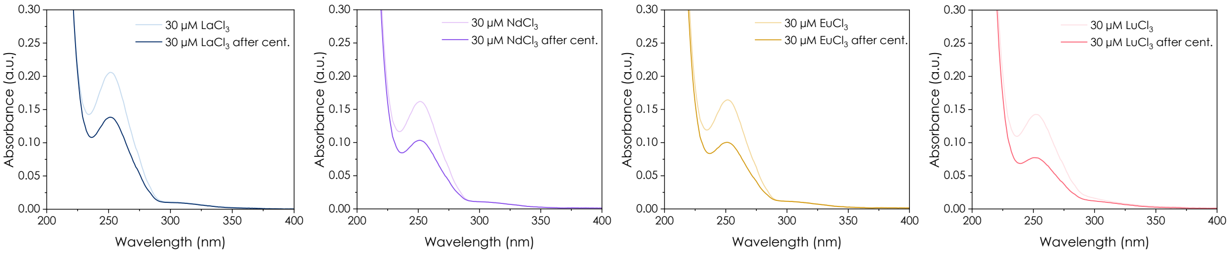

Figure S6 UV-vis spectra of a $LnCl_3$ (Ln = La, Nd, Eu and Lu) to MLL titration experiment using 10 µM MLL in buffer (10 mM MOPSO, 100 mM KCl, pH 6.0). After the addition of a 3-fold excess the sample was centrifuged and measured again showing a significant decrease in absorbance. The spectra correspond to the spectra shown in Figure 2. Within the UV-vis spectra subtle trend from La^{3+} to Lu^{3+}. can be observed. For La^{3+}, the smallest reduction of absorption (with and without centrifugation (Figure 2A) is observed. In contrast, Nd^{3+} and Eu^{3+} exhibit similar changes, while Lu^{3+} shows the most significant decrease in absorbance after centrifugation.

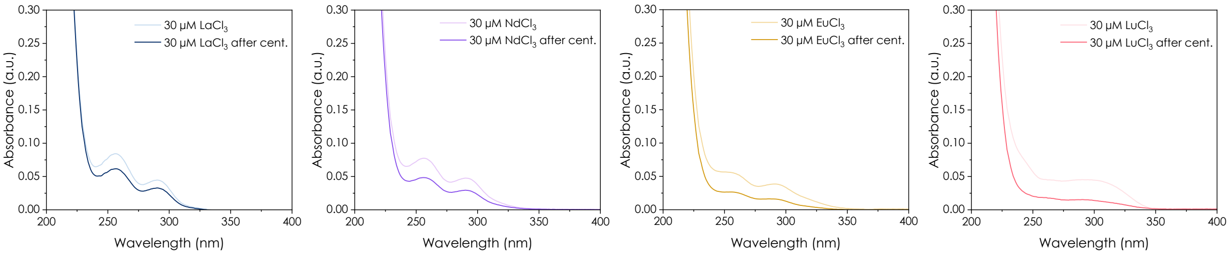

Figure S7 UV-vis spectra of RPB B (10 µM) in buffer (10 mM MOPSO, 100 mM KCl, pH 6.0) after the addition of 30 µM $LnCl_3$ (Ln = La, Nd, Eu, Lu, left to right) and after centrifuging the sample and measuring the obtained supernatant. A significant decrease in absorbance can be observed. The Eu spectra were measured directly after the titration series shown in Figure 2 while the spectra for La, Nd and Lu were obtained after adding the volumes for all four titration steps at once followed by a 10 min equilibration time.

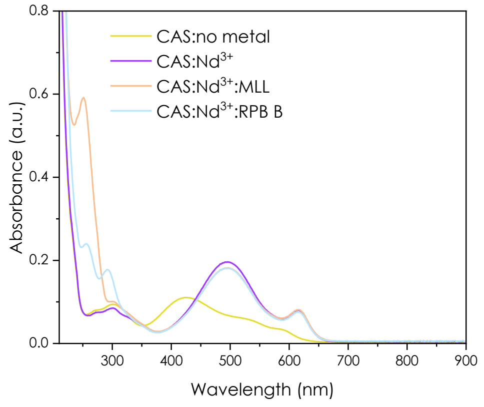

Figure S8 CAS competition experiment of RPB B and MLL in MOPSO buffer (10 mM, 100 mM KCl, pH 6.0) with $NdCl_3$ (final concentration: CAS/ HDTMA (18.75 µM/ 200 µM), $NdCl_3$ (18.75 µM), Ligand (18.75 µM)).

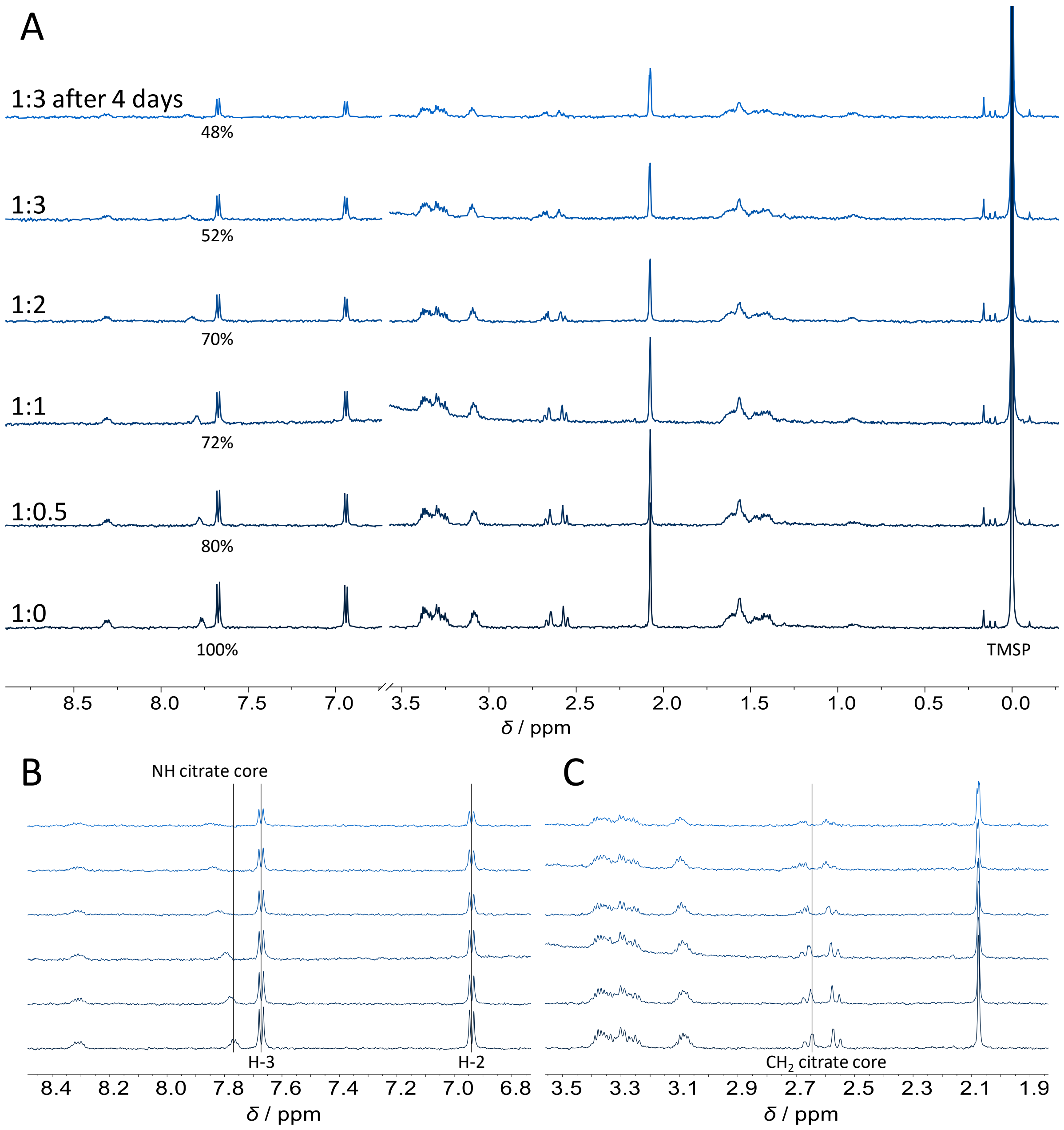

Figure S9 Titration of $LaCl_3$ to MLL (30 µM) in increasing concentrations (0 µM to 90 µM) in D_2O:H_2O (1:9) + 0.003% TMSP-d_4 (600 MHz). All spectra were normalised to the signal of the internal standard TMSP-d_4. **(A)** Overview of the full titration series; the ligand to metal ratio is given next to each spectrum. A steady decrease of the signals associated with MLL[4] with an increasing La^{3+}-concentration can be observed. The percental reduction of the MLL concentration (sample without metal was set to 100%) is based on the comparison of the integrals of H-3 at 7.67 ppm and the internal standard TMSP-d_4. The spectral changes strongly suggest the precipitation of a MLL-La^{3+}-complex. **(B)** Zoom-in of the aromatic region showing no shift for both aromatic signals H-2 at 7.67 ppm and H-3 at 7.67 ppm (see Section 1.2 compound 13b for the numbering of MLL) and only a minor downfield shift for the proton associated with the NH (7.77 ppm) of the citrate core. **(C)** Zoom-in showing the signals of the diastereotropic CH_2 of the citrate core (2.56, 2.66 ppm) for which a slight downfield shift can be observed. For both the NH as well as the CH_2 signals the shift could be caused by slight pH changes during the experiment, as the solution was not buffered.

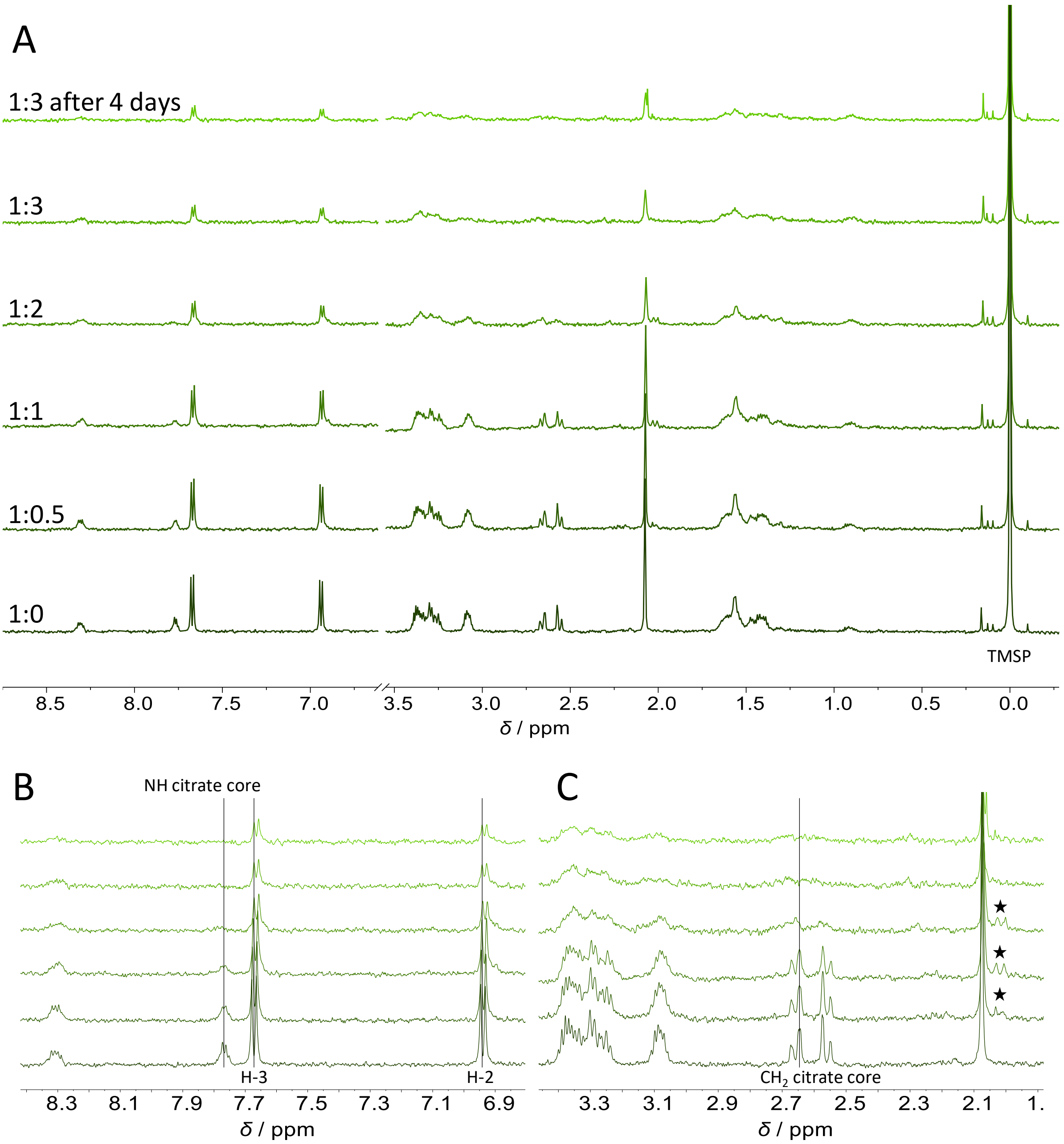

Figure S10 Titration of $Ga(NO_3)_3$ to MLL (30 µM) in increasing concentrations (0 µM to 90 µM) in D_2O:H_2O (1:9) + 0.003% TMSP-*d*$_4$ (600 MHz). All spectra were normalised to the signal of the internal standard TMSP-*d*$_4$. (**A**) Overview of the full titration series; the ligand to metal ratio is given next to each spectrum. The spectral changes suggest that MLL can form a soluble complex with Ga^{3+}. **(B)** Zoom-in of the titration series showing slight upfield shifts for the aromatic signals H-3 and H-2 (see Section 1.2 compound 13b for the numbering of MLL) while the signal of the NH (7.77 ppm) of the citrate core shows no significant shift, but decreases, gets broader and vanishes at a 1:3 ligand to metal concentration. **(C)** Zoom-in of the titration series showing that the signals of the CH_2 (2.56, 2.66 ppm) signals of the citrate core do not shift, but decrease, get broader and vanish at a 1:3 ligand to metal concentration. In addition, next to the signal of the acetyl group (2.08 ppm) a doublet (marked with a star) appears from ligand to metal ratios of 1:0.5 to 1:2 and vanishes at 1:3.

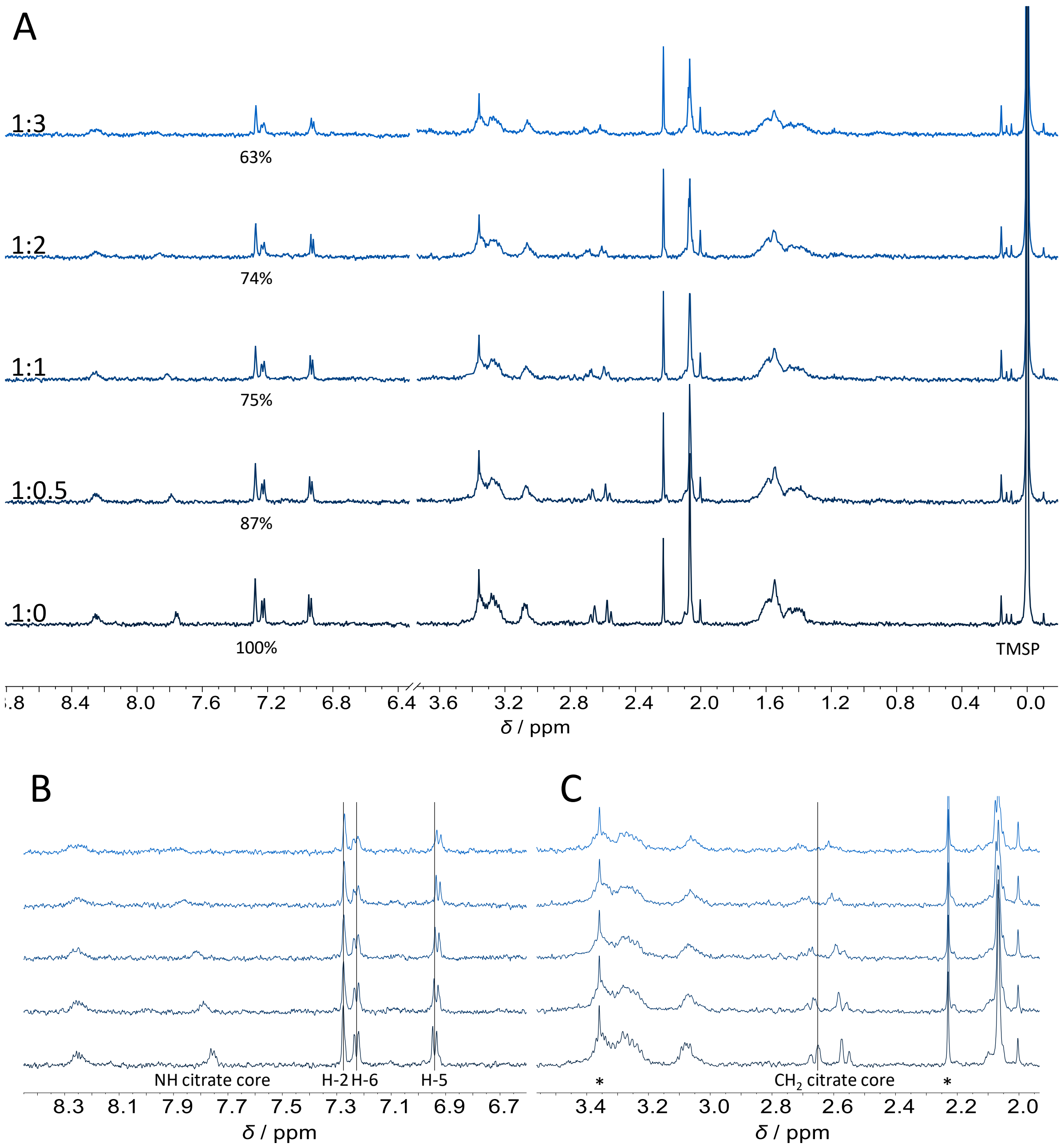

Figure S11 Titration of $La(NO_3)_3$ to RPB B (60 µM) in increasing concentrations (0 µM to 180 µM) in D_2O:H_2O (1:9) + 0.003% TMSP-d_4 (600 MHz). All spectra were normalised to the signal of the internal standard TMSP-d_4. **(A)** Overview of the full titration series; the ligand to metal ratio is given next to each spectrum. A steady decrease of all signals associated with RPB B can be observed. The percental reduction of the RPB B concentration (sample without metal was set to 100%) is based on the comparison of the integrals of H-2 and H-6 and the internal standard TMSP-d_4. The spectral changes suggest the presence of a small amount of soluble RPB B-La^{3+}-complex as well as the possible precipitation of a complex. **(B)** Zoom-in of the titration series showing that the signals H-2 (7.28 ppm) and H-5 (6.94 ppm), next to the catechol hydroxyl groups, shift slightly upfield, but no shift for H-6 can be observed. Furthermore, a minor downfield shift can be observed for the protons associated with the NH of the citrate core. **(C)** Zoom-in of the titration series showing a slight downfield shift of signals associated with the diastereotropic CH_2 of the citrate core (2.56, 2.66 ppm). For both the NH (7.76 ppm) as well as the CH_2 signals the shift could be caused by slight pH changes during the experiment, as the solution was not buffered. Solvent impurities are marked with an asterisk.

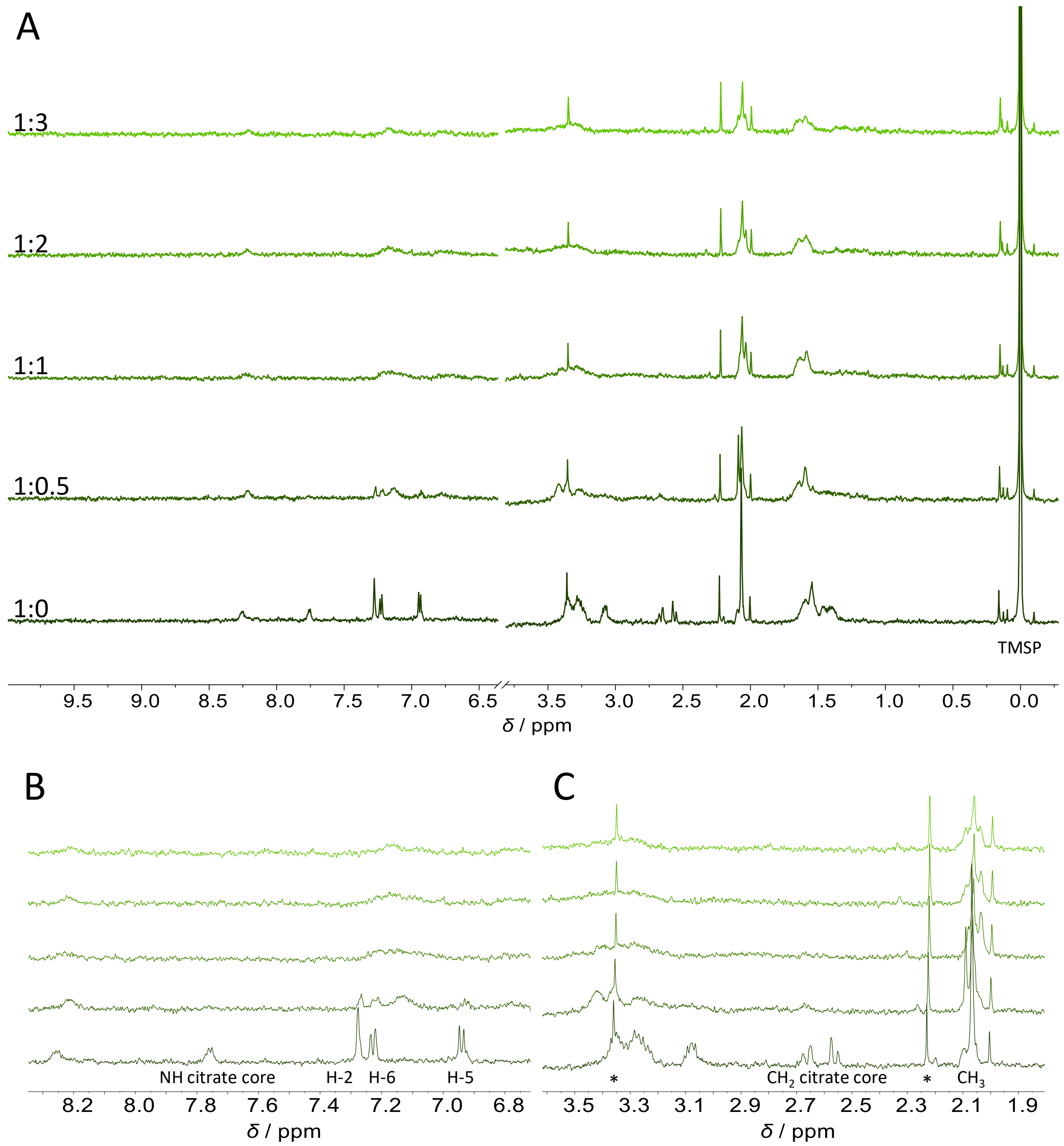

Figure S12 Titration of $Ga(NO_3)_3$ to RPB B (60 µM) in increasing concentrations (0 µM to 180 µM) in D_2O:H_2O (1:9) + 0.003% TMSP-d_4 (600 MHz). All spectra were normalised to the signal of the internal standard TMSP-d_4. **(A)** Overview of the full titration series; the ligand to metal ratio is given next to each spectrum. Already the addition of 0.5 equiv. Ga^{3+} induces major spectral changes involving signal broadening, shifts and new signal sets. The biggest effect can be observed for the signals of the citrate centre **(C)** and the aromatic signals **(B)** fitting the suggested binding motifs of RPB B. Solvent impurities are marked with an asterisk.

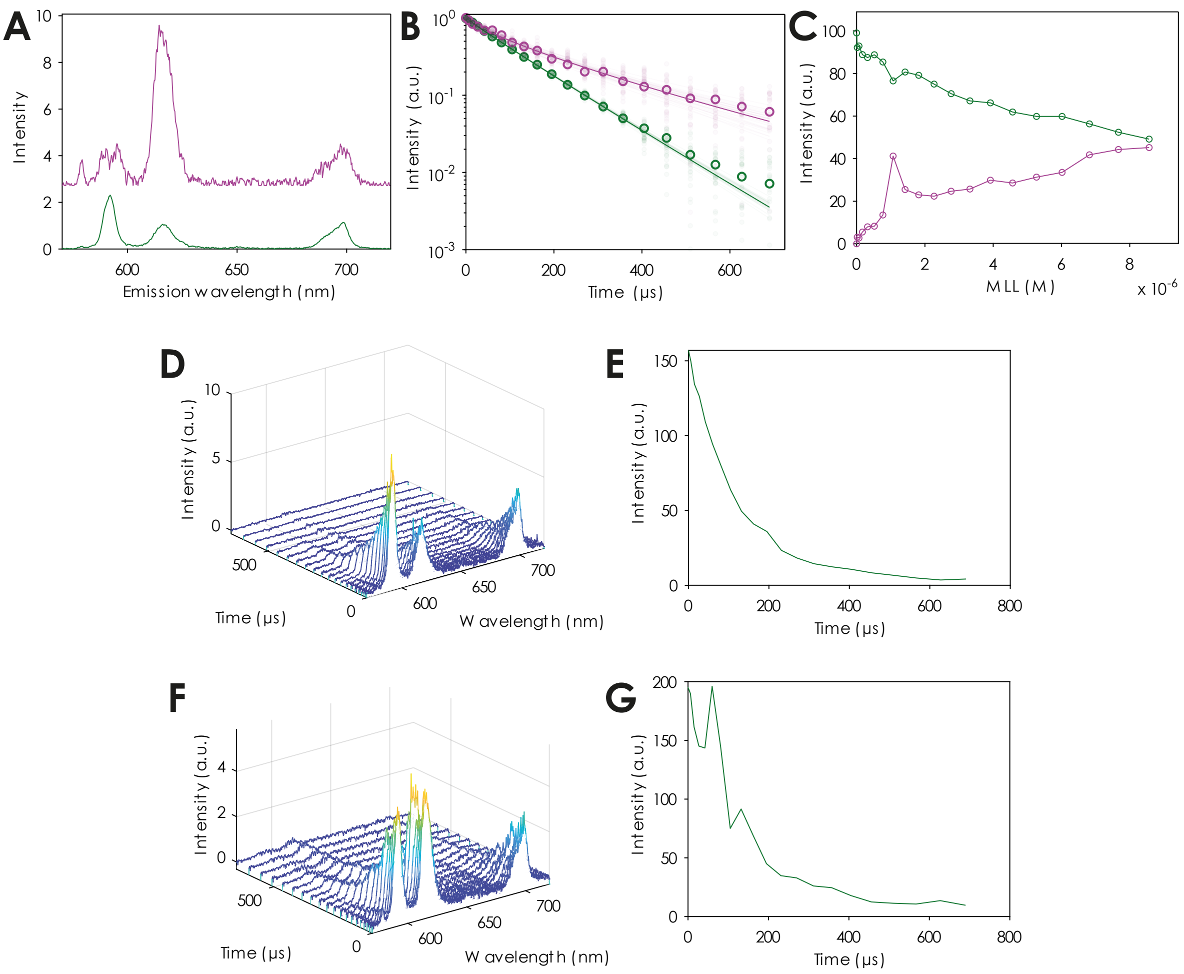

Figure S13 MLL to 1 µM Eu^{3+} titration experiment (10 mM MOPSO, 100 mM KCl, pH 6.6). The concentration of MLL was increased stepwise from 0 µM to 8.6 µM. The data set as a whole indicates an MLL concentration-dependent particle-containing suspension. **(A)** Stacked spectra of the observed species, clearly showing the formation of a MLL-Eu^{3+} complex; magenta: MLL-Eu^{3+}-species; green: Eu^{3+}-aquo ion. The obtained data for MLL is unexpectedly noisy – usually Eu^{3+}-complexes have a higher quantum yield and thus a better signal-to noise ratio than the Eu^{3+}-aquo ion. **(B)** Luminescence decay of the observed species; green: Eu^{3+}-aquo ion (112 µs); pink: MLL-Eu^{3+}-species with non-monoexponential (here stretched exponential) luminescence decay. **(C)** Distribution of the titration series as extracted by PARAFAC; magenta: MLL-Eu^{3+}-species; green: Eu^{3+}-aquo ion. **(D)** Matrix of the first sample (Eu^{3+}-aquo ion) and corresponding luminescence decay of the $^5D_0 \rightarrow {}^7F_2$ transition shown in **(E).** **(F)** Matrix of the last titration step (highest MLL concentration) with irregular scattering and corresponding luminescence decay of the $^5D_0 \rightarrow {}^7F_2$ transition shown in **(G).**

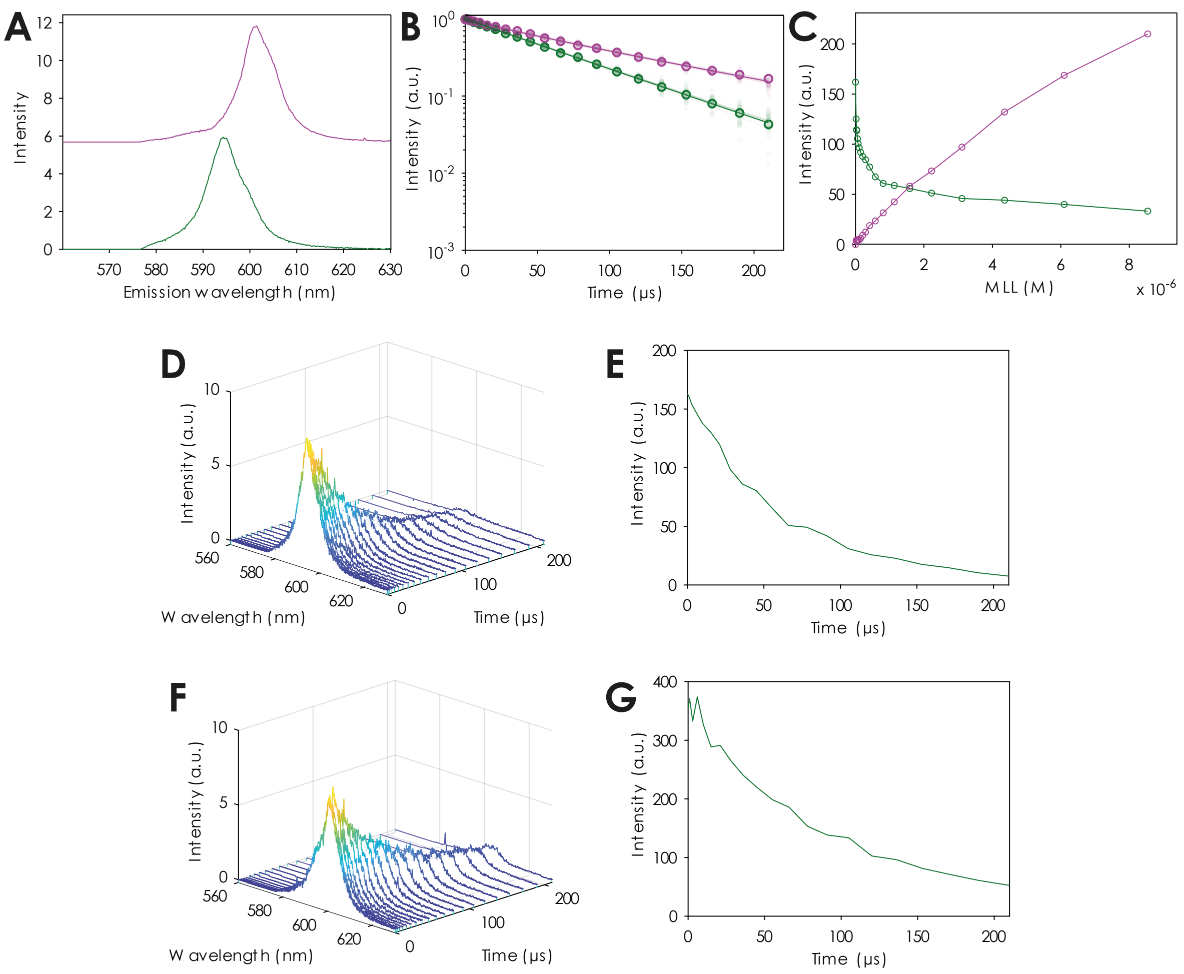

Figure S14 MLL to 100 nM Cm^{3+} titration experiment (10 mM MOPSO, 100 mM KCl, pH 6.0). The concentration of MLL was increased stepwise from 0 µM to 8.5 µM. This data set also points to a particle-containing suspension. **(A)** Stacked spectra of the observed species, clearly showing the formation of a MLL-Cm^{3+}-complex; magenta: MLL-Cm^{3+}-species; green: Cm^{3+}-aquo ion. **(B)** Luminescence decay of the observed species; magenta: MLL-Cm^{3+}-species; green: Cm^{3+}-aquo ion. MLL species with non-monoexponential luminescence decay. **(C)** Distribution of the titration series; magenta: MLL-Cm^{3+}-species; green: Cm^{3+}-aquo ion. Inconsistent distribution showing an unexpected progression for the decrease of the Cm^{3+}-aquo ion in combination with an emerging MLL-Cm^{3+}-species. Especially well observable over the first titration steps in which a strong decrease of the signal associated with the Cm^{3+}-aquo ion can be seen, not matching the slow increase of the signal associated with the MLL-Cm^{3+}. **(D)** Matrix of the first sample (Cm^{3+}-aquo ion) and corresponding luminescence decay in **(E)**. **(F)** Matrix of titration step 18 (out of 20; excess of MLL) with irregular scattering and corresponding luminescence decay shown in **(G)**.

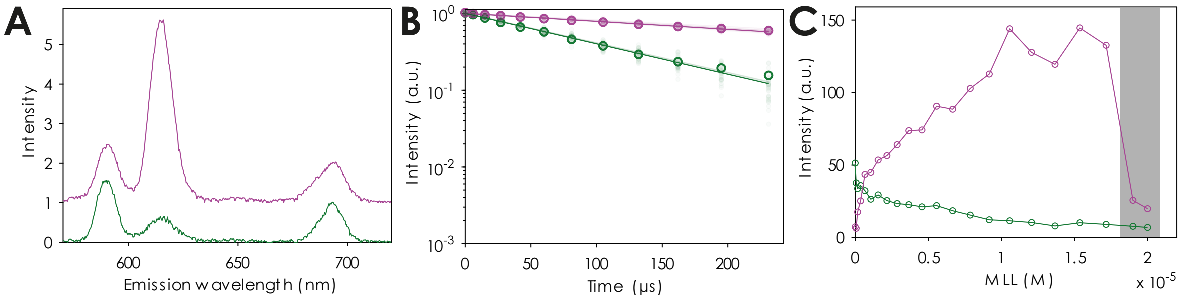

Figure S15 MLL to 200 nM Eu^{3+} titration experiment (10 mM MOPSO, 100 mM KCl, pH 6.0). The concentration of MLL was increased stepwise from 0 µM to 17.2 µM. **(A)** Stacked spectra of the observed species, clearly showing the formation of a MLL-Eu^{3+}-complex; magenta: MLL-Eu^{3+}-species; green: Eu^{3+}-aquo ion. **(B)** Luminescence decay of the observed species; magenta: MLL-Eu^{3+}-species; green: Eu^{3+}-aquo ion. **(C)** Distribution of the titration series; magenta: MLL-Eu^{3+}-species; green: Eu^{3+}-aquo ion. In the last two data points highlighted in grey the concentration of MLL was not further increase, but the solution obtained from the last titration step centrifuged and the supernatant measured two times. A significant drop for the previously observed MLL-Eu^{3+}-species can be seen, indicating that the complex precipitated and was almost completely removed by centrifugation.

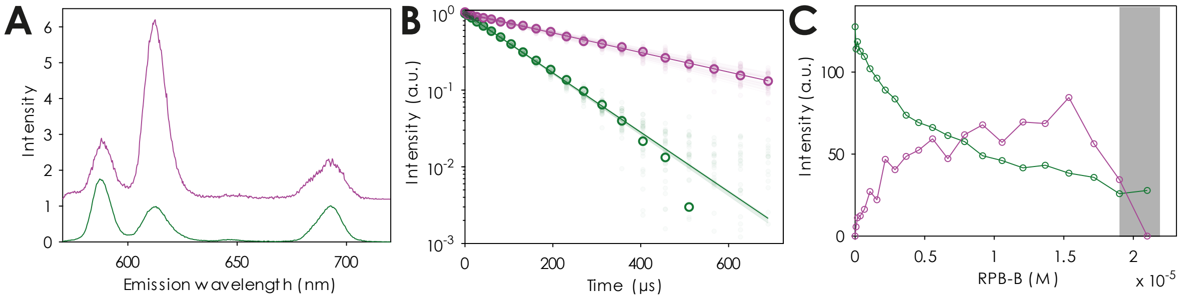

Figure S16 RPB B to 200 nM Eu^{3+} titration experiment (10 mM MOPSO, 100 mM KCl, pH 6.0). The concentration of RPB B was increased stepwise from 0 µM to 17.2 µM. **(A)** Stacked spectra of the observed species, clearly showing the formation of a RPB B-Eu^{3+}-complex; pink: RPB B-Eu^{3+}-species; green: Eu^{3+}-aquo ion. **(B)** Luminescence decay of the observed species; magenta: RPB B-Eu^{3+}-species; green: Eu^{3+}-aquo ion. **(C)** Distribution of the titration series; magenta: RPB B-Eu^{3+}-species; green: Eu^{3+}-aquo ion. Inconsistent and scattering distribution. In the last data point, highlighted in grey, the concentration of RPB B was not further increased, but the solution obtained from the last titration step centrifuged and the supernatant measured again. A significant drop of the previously observed RPB B-Eu^{3+}-species can be seen, indicating that the complex precipitated and was removed by centrifugation.

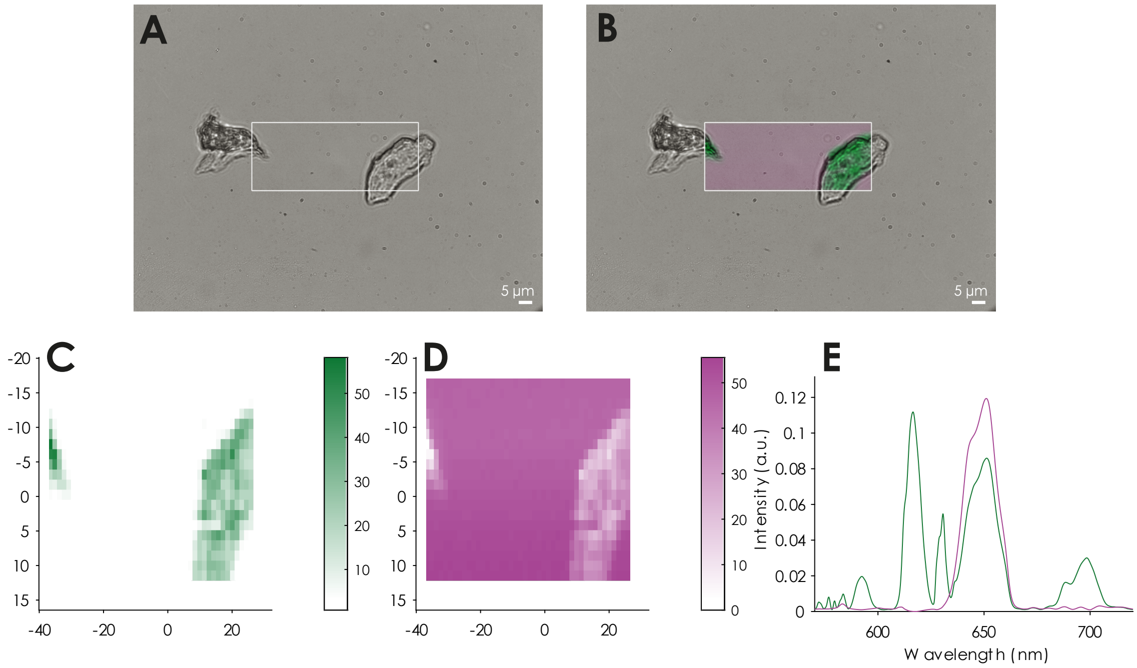

Figure S17 Chemical microscopy of presumably MLL-Eu^{3+}-complex. **(A)** Microscopic image (60-fold magnification) of particles from centrifuged MLL-Eu^{3+}-mixture highlighting the mapped region of interest (ROI, white box). **(B)** Overlay with the deconvoluted maps shown in **(C)** and **(D)**. **(E)** Single component spectra; magenta: Raman band of water; green: Eu^{3+}-MLL-complex. The emission spectrum of the MLL-complex is contaminated with the Raman bands from the organic matrix.

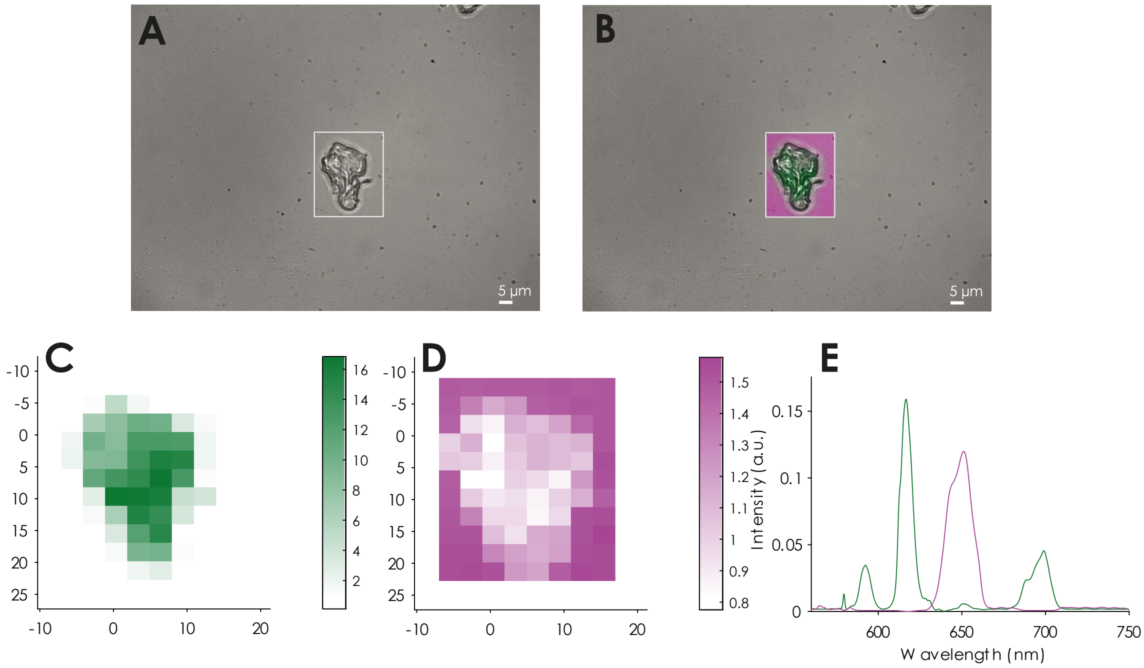

Figure S18 Chemical microscopy of presumably MLL-Eu^{3+}-complex. **(A)** Microscopic image (60-fold magnification) of particles from centrifuged MLL-Eu-mixture highlighting mapped region of interest (ROI, white box) **(B)** Overlay with the deconvoluted maps shown in **(C)** and **(D). (E)** Single component spectra; magenta: Raman band of water; green Eu^{3+}-MLL-complex.

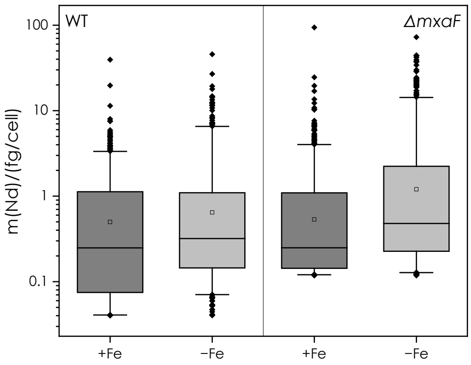

Figure S19 Boxplot of the distributions underlying Figure 3A. Biological replicates are grouped into a single sample per figure. Boxes fence values between 10th and 90th percentiles, whiskers represent 1st and 99th percentiles. Arithmetic mean (hollow squares) and median values (bars) are provided inside the boxes. Outliers are marked as filled squares.

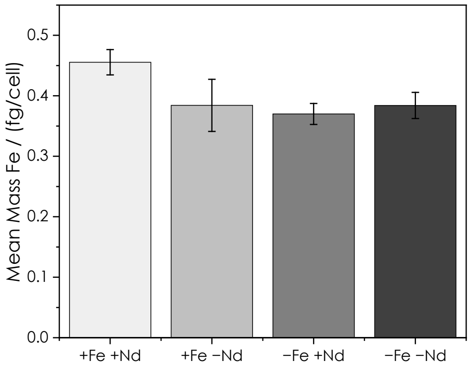

Figure S20 Bar-plot of the scICP-MS quantification of Fe in WT cells grown in Fe-replete and Fe-deplete medium (½ Hypho medium, 50 mM MeOH) in the presence and absence of 2 µM $NdCl_3$. Two-way ANOVA was conducted to examine the influence of the Nd and Fe concentration in the culture medium on intracellular mass of Fe per cell. No significant interaction was found ($F(1,7) = 2.04351$, $p = 0.19593$). Additionally, the effect of Fe concentration and Nd concentration were insignificant for intracellular Fe levels ($p = 0.19451$, $p = 0.36975$).

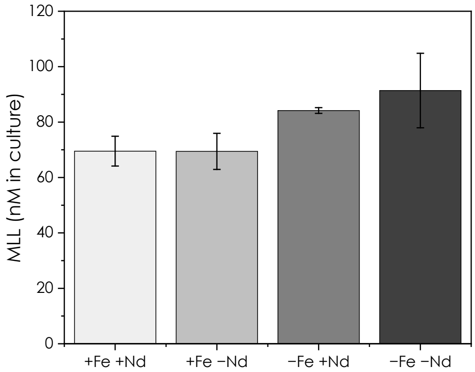

Figure S21 Bar-plot of the determined MLL concentration in WT supernatant samples grown in Fe-replete and Fe-deplete medium (½ Hypho medium, 50 mM MeOH) in the presence and absence of 2 µM $NdCl_3$. The error bars give the standard deviation between the separately analysed, pooled samples n(WT) = 3. Two-way ANOVA was conducted to examine the influence of Nd and Fe on the MLL concentration in the supernatant samples. No significant interaction between the applied Fe and Nd concentration was found ($F(1,8) = 0.42304$, $p = 0.\ 53365$). However, the Fe concentration significantly influences the MLL concentration ($p = 0.01161$) while no significant influence of the Nd concentration was found ($p = 0.54274$).

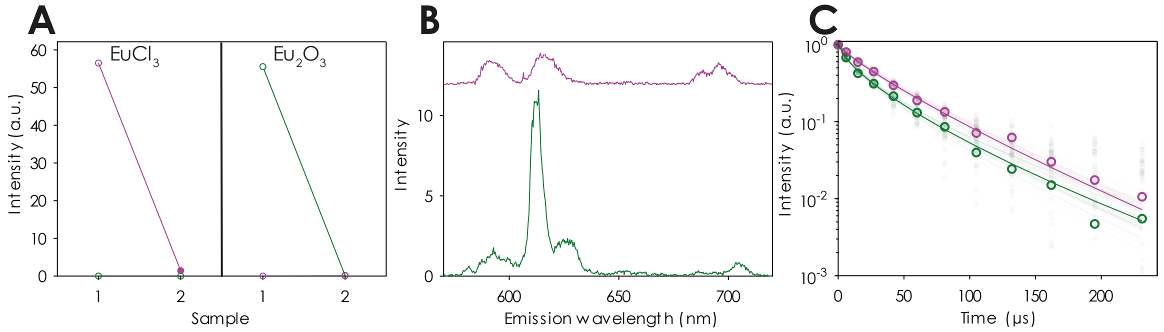

Figure S22 TRLFS measurements of 2 µM $EuCl_3$ versus 1 µM Eu_2O_3 in ½ Hypho medium after incubation at 30 °C overnight. **(A)** Species distribution. 1: incubated Sample measured after vortexing; 2: supernatant after centrifugation. **(B)** Corresponding spectra to the species distribution shown in A. **(C)** Corresponding lifetimes to the species distribution shown in A. Magenta: Phosphate-like species;[13] also observed in Figure S5. Green: solid Eu_2O_3.

Table S5 Experimental $^{TW}CCS_{N2}$ of $[MLL{-}H{+}M]^{2+}$ and $[RPB\ B{-}H{+}M]^{2+}$ (M = 3H, Fe, Sc, Y, La-Lu, except Pm)

	$[MLL{-}H\ {+}M]^{2+}$ $^{TW}CCS_{N2}$(Å^2)	$[RPB\ B{-}H\ {+}M]^{2+}$ $^{TW}CCS_{N2}$(Å^2)
**3H**	355.0	353.1
**Fe**	303.8	304.3
**Sc**	311.9	311.0
**Y**	303.3	304.3
**La**	306.5	306.8
**Ce**	306.1	306.5
**Pr**	305.2	306.0
**Nd**	304.8	305.7
**Sm**	304.4	305.0
**Eu**	303.7	304.8
**Gd**	303.4	304.6
**Tb**	303.6	304.3
**Dy**	302.8	304.0
**Ho**	302.5	303.8
**Er**	302.2	303.5
**Tm**	301.9	303.5
**Yb**	301.6	303.3
**Lu**	301.8	303.3

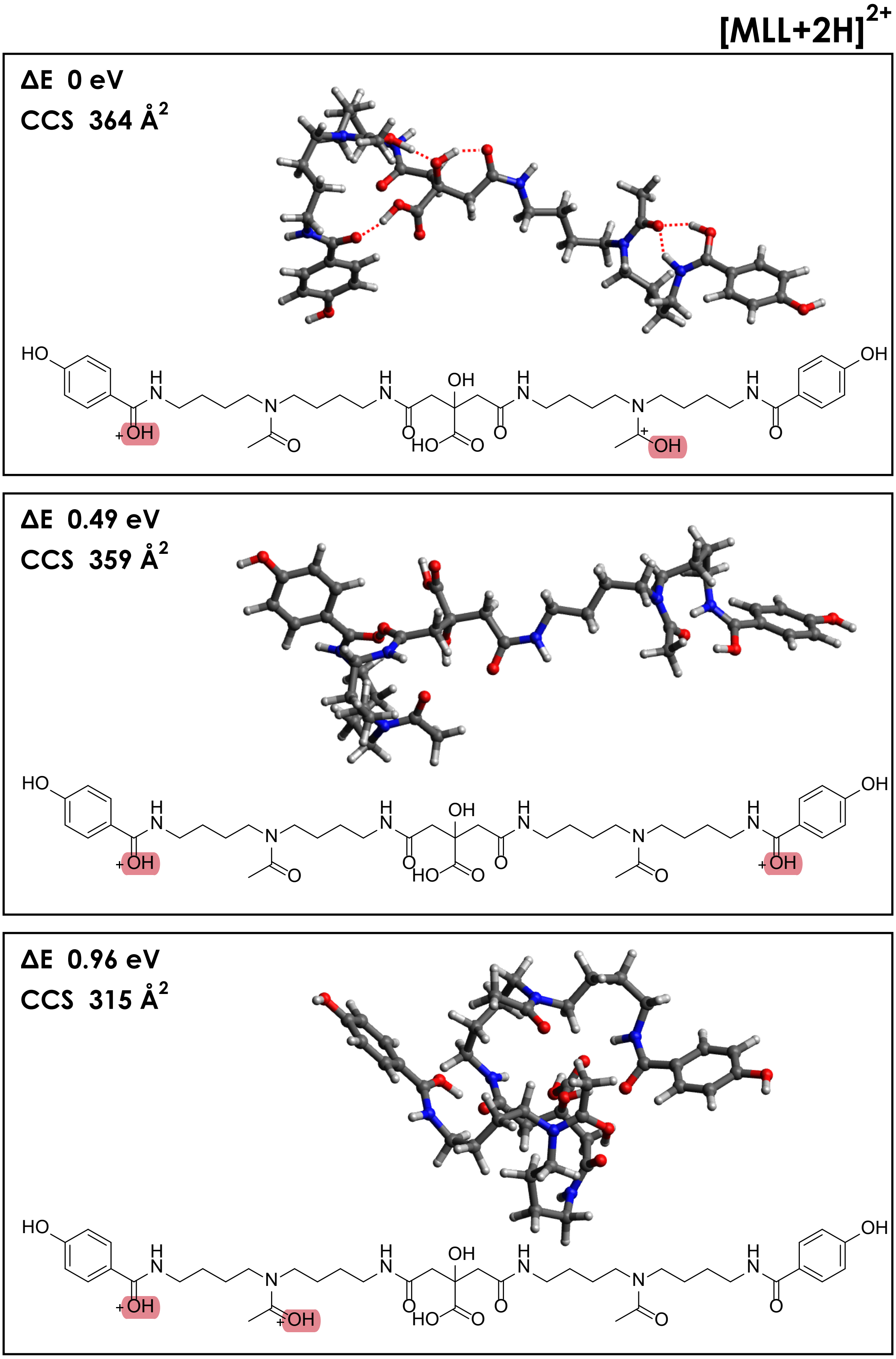

Figure S23 DFT optimised structure of $[MLL+2H]^{2+}$ as well as structural formula of MLL with protonation sites indicated in red.

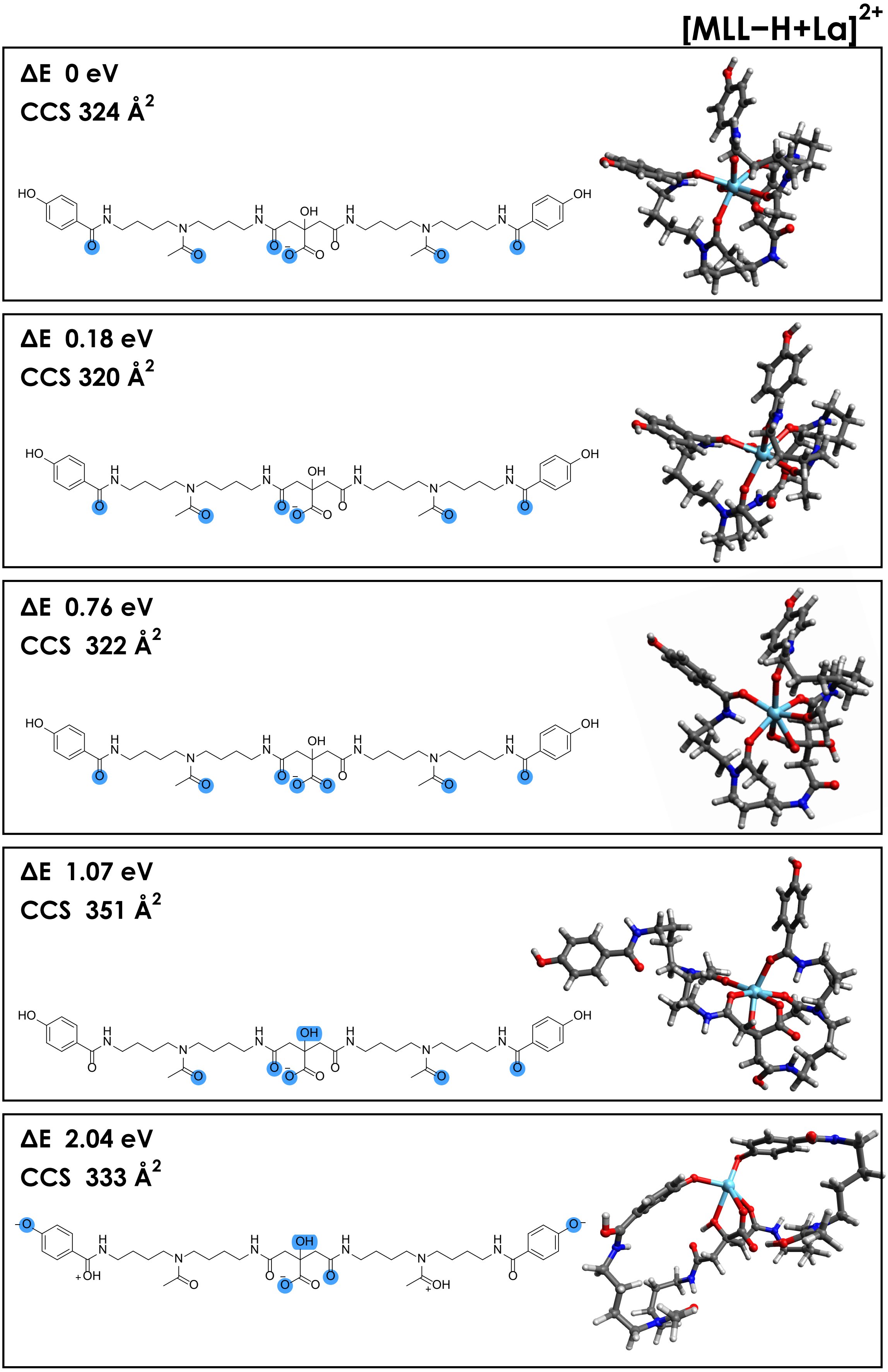

Figure S24 DFT-optimised structures of $[MLL{-}H{+}La]^{2+}$ with relative energies and calculated collision cross sections. Coordinating groups highlighted in blue.

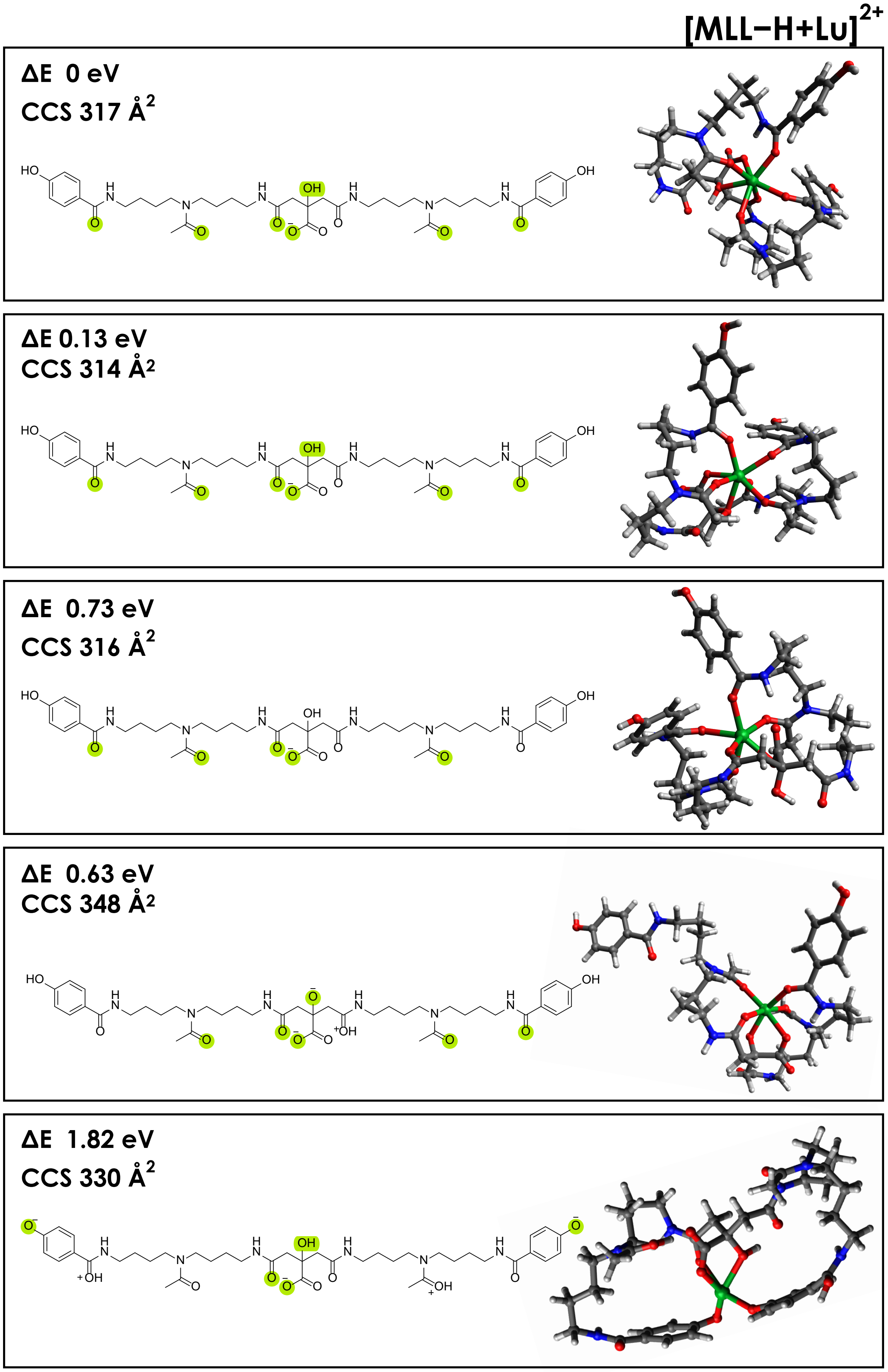

Figure S25 DFT Optimised structures of $[MLL{-}H{+}Lu]^{2+}$ with relative energies and calculated collision cross sections. Coordinating groups highlighted in green.

$[MLL-H+Sc]^{2+}$

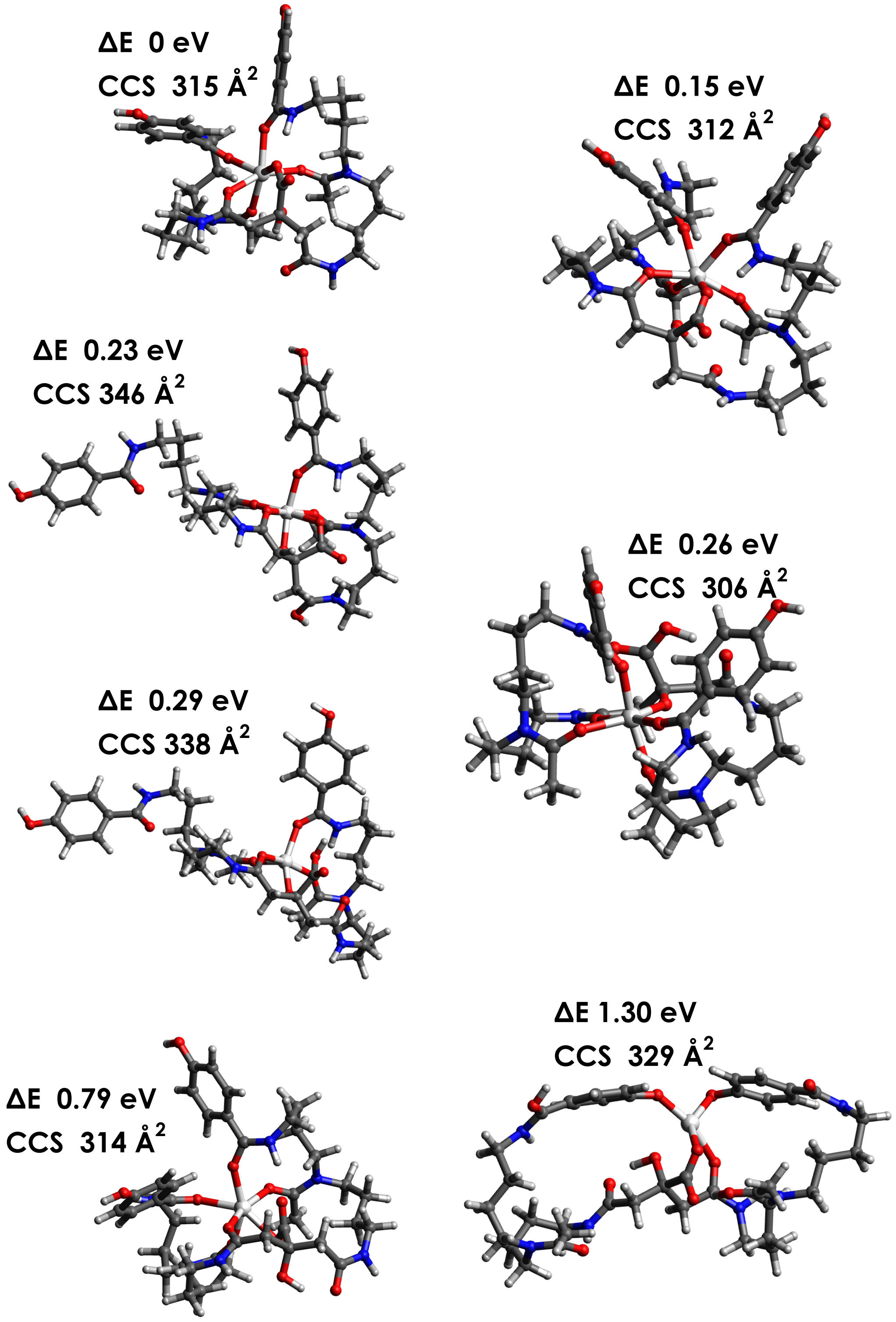

Figure S26 DFT Optimised structures of $[MLL-H+Sc]^{2+}$ and calculated collision cross sections.

$[RPB\ B+2H]^{2+}$

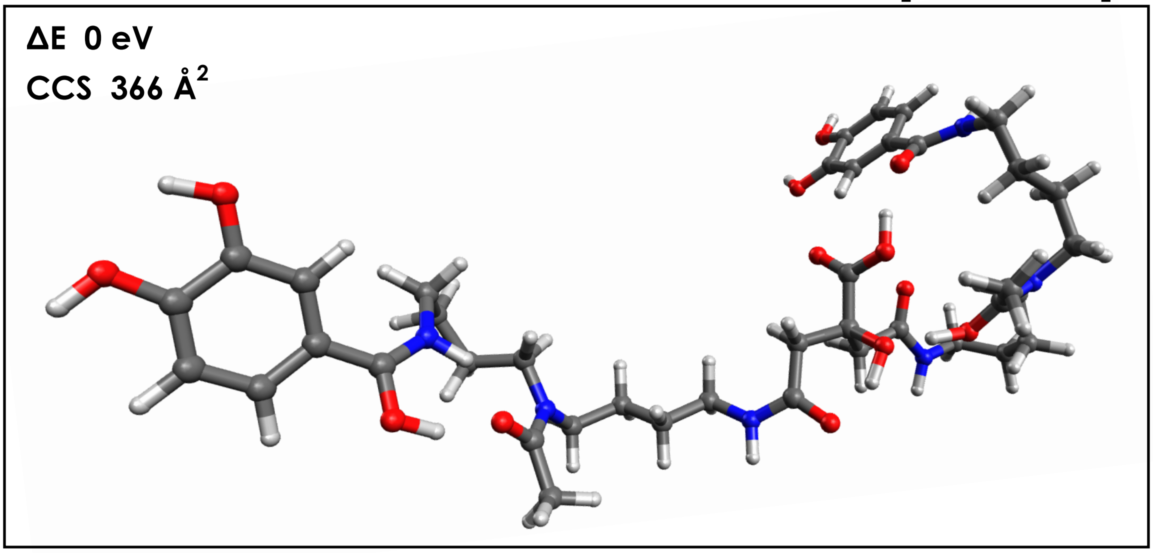

Figure S27 DFT optimised structures of $[RPB\ B+2H]^{2+}$ and calculated collision cross section.

$[RPB\ B{-}H{+}La]^{2+}$

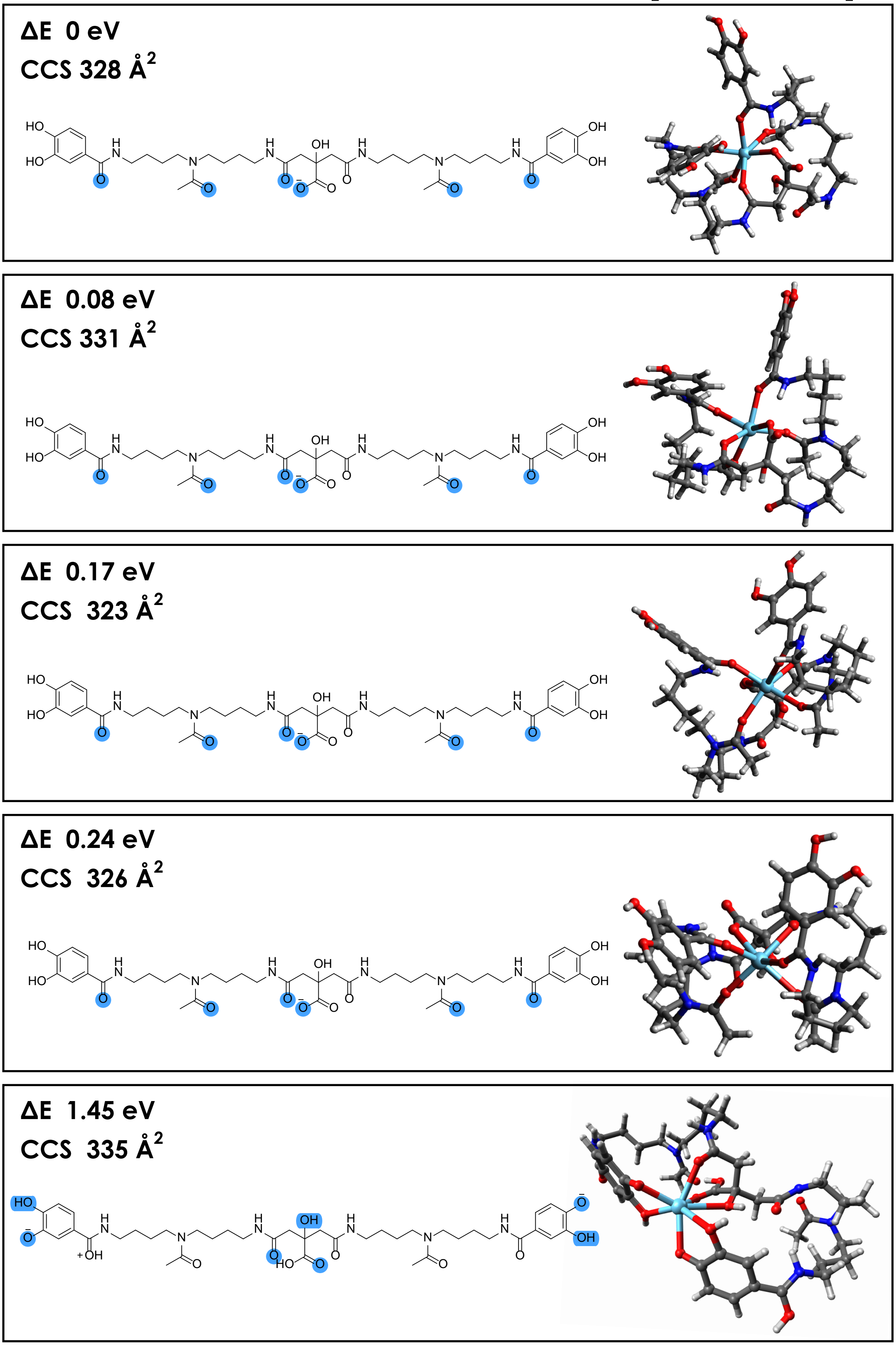

Figure S28 DFT Optimised structures of $[RPB\ B{-}H{+}La]^{2+}$ and calculated collision cross sections. Coordinating groups highlighted in blue.

[RPB B−H+Sc]$^{2+}$

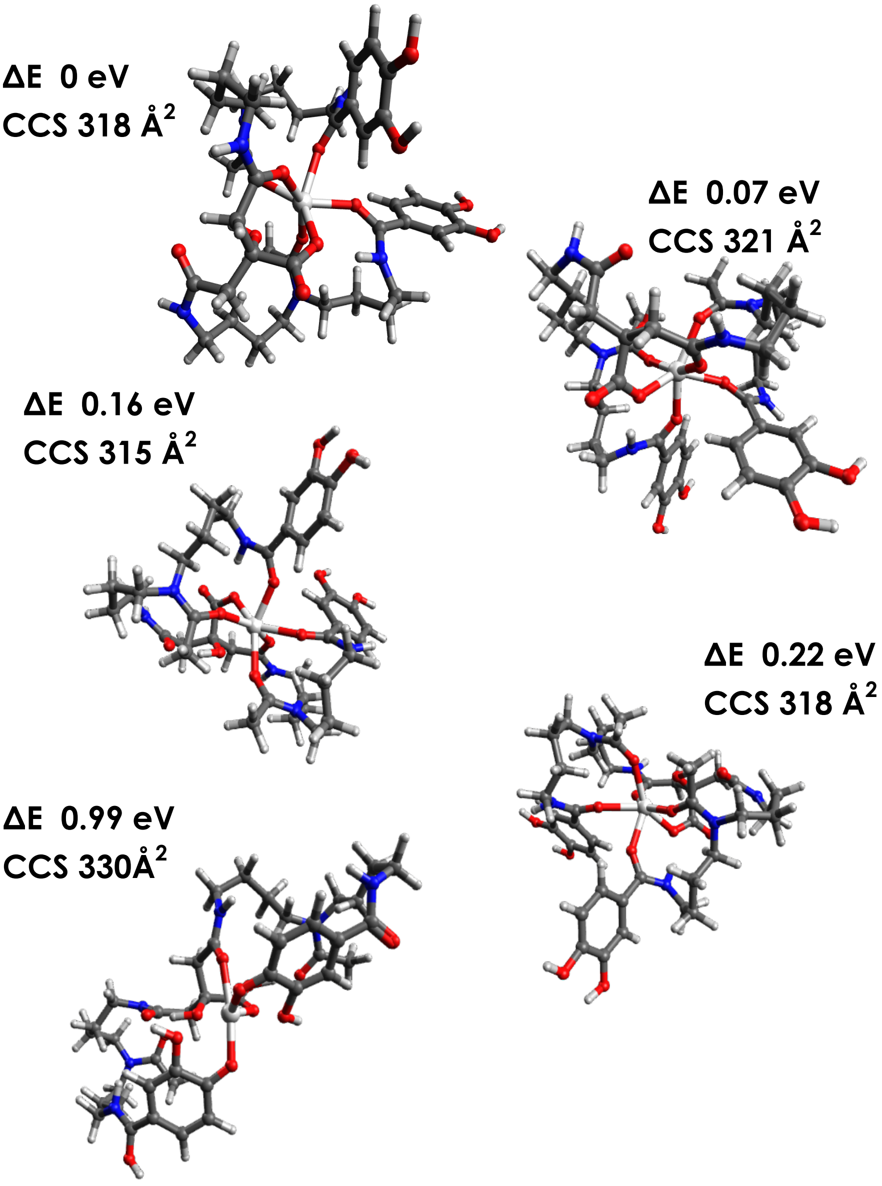

Figure S29 DFT Optimised Structures of [RPB B−H+Sc]$^{2+}$ and calculated collision cross sections.

6 Data Availability

Additional information on the synthesis as well as analysis of the synthesised compounds described within this Supporting Information is available at the Chemotion Online Repository[31] under: https://dx.doi.org/10.14272/collection/MWM_2024-11-19

UV-vis (.csv), TRLFS (.sif and processed), scICP-MS event (.csv), NMR (Bruker raw data) and chemical microscopy data (raw data and processed) as well as quantum chemical calculations (.xyz) shown in this manuscript and its Supplementary Information are available at the RODARE repository: https://doi.org/10.14278/rodare.3273

7 References

1 R. A. Gardner, R. Kinkade, C. Wang, and Phanstiel, Total Synthesis of Petrobactin and Its Homologues as Potential Growth Stimuli for Marinobacter hydrocarbonoclasticus, an Oil-Degrading Bacteria, *J. Org. Chem.*, 2004, **69**, 3530–3537.

2 P. A. Turhanen, J. Leppänen and J. J. Vepsäläinen, Green and Efficient Esterification Method Using Dried Dowex H+/NaI Approach, *ACS Omega*, 2019, **4**, 8974–8984.

3 H. Guo, S. A. Naser, G. Ghobrial, and Phanstiel, Synthesis and Biological Evaluation of New Citrate-Based Siderophores as Potential Probes for the Mechanism of Iron Uptake in Mycobacteria, *J. Med. Chem.*, 2022, **45**, 2056–2063.

4 A. M. Zytnick, S. M. Gutenthaler-Tietze, A. T. Aron, Z. L. Reitz, M. T. Phi, N. M. Good, D. Petras, L. J. Daumann and N. C. Martinez-Gomez, Identification and characterization of a small-molecule metallophore involved in lanthanide metabolism, *PNAS*, 2024, **121**, e2322096121.

5 C. A. Andersson and R. Bro, The N-way Toolbox for MATLAB, *Chemom. Intell. Lab. Syst.*, 2000, **52**, 1–4.

6 B. Drobot, R. Steudtner, J. Raff, G. Geipel, V. Brendler and S. Tsushima, Combining luminescence spectroscopy, parallel factor analysis and quantum chemistry to reveal metal speciation – a case study of uranyl(VI) hydrolysis, *Chem. Sci.*, 2015, **6**, 964–972.

7 B. Drobot, A. Bauer, R. Steudtner, S. Tsushima, F. Bok, M. Patzschke, J. Raff and V. Brendler, Speciation Studies of Metals in Trace Concentrations: The Mononuclear Uranyl(VI) Hydroxo Complexes, *Anal. Chem.*, 2016, **88**, 3548–3555.

8 M. Leutenegger, Andor SIF image reader https://www.mathworks.com/matlabcentral/fileexchange/11224-andor-sif-image-reader, 2021.

9 S. Friedrich, C. Sieber, B. Drobot, S. Tsushima, A. Barkleit, K. Schmeide, T. Stumpf and J. Kretzschmar, Eu(III) and Cm(III) Complexation by the Aminocarboxylates NTA, EDTA, and EGTA Studied with NMR, TRLFS, and ITC—An Improved Approach to More Robust Thermodynamics, *Molecules*, 2023, **28**, 4881.

10 L. Lopez-Odriozola, S. Shaw, L. Abrahamsen-Mills, C. Waters and L. S. Natrajan, Identification and Quantification of Multiphase U(VI) Speciation on Gibbsite with pH Using TRLFS and PARAFAC of Excitation Emission Matrices, *Environ. Sci. Technol.*, 2024, **58**, 17916–17925.

11 D. S. Smith, Solution of Simultaneous Chemical Equilibria in Heterogeneous Systems: Implementation in Matlab, *Chemistry Faculty Publications,* 2019, **14** https://scholars.wlu.ca/chem_faculty/14.

12 N. F. Delaney, M. E. Kaczmarek, L. M. Ward, P. K. Swanson, M.-C. Lee and C. J. Marx, Development of an Optimized Medium, Strain and High-Throughput Culturing Methods for Methylobacterium extorquens, *PLoS ONE*, 2013, **8**, e62957.
13 M. Vogel, R. Steudtner, T. Fankhänel, J. Raff and B. Drobot, Spatially resolved Eu(III) environments by chemical microscopy, *Analyst*, 2021, **146**, 6741–6745.
14 S. M. Stow, T. J. Causon, X. Zheng, R. T. Kurulugama, T. Mairinger, J. C. May, E. E. Rennie, E. S. Baker, R. D. Smith, J. A. McLean, S. Hann and J. C. Fjeldsted, An Interlaboratory Evaluation of Drift Tube Ion Mobility-Mass Spectrometry Collision Cross Section Measurements, *Anal. Chem.*, 2017, **89**, 9048–9055.
15 H. E. Pace, N. J. Rogers, C. Jarolimek, V. A. Coleman, C. P. Higgins and J. F. Ranville, Determining Transport Efficiency for the Purpose of Counting and Sizing Nanoparticles via Single Particle Inductively Coupled Plasma Mass Spectrometry, *Anal. Chem.*, 2011, **83**, 9361–9369.
16 M. Elinkmann, S. Reuter, M. Holtkamp, S. Heuckeroth, A. Köhrer, K. Kronenberg, M. Sperling, O. Rubner, C. D. Quarles, M. Hippler and U. Karst, Improving detection thresholds and robust event filtering in single-particle and single-cell ICP-MS analysis, *J. Anal. At. Spectrom.*, 2023, **38**, 2607–2618.
17 P. Pracht, F. Bohle and S. Grimme, Automated exploration of the low-energy chemical space with fast quantum chemical methods, *Phys. Chem. Chem. Phys.*, 2020, **22**, 7169–7192.
18 S. Grimme, C. Bannwarth and P. Shushkov, A Robust and Accurate Tight-Binding Quantum Chemical Method for Structures, Vibrational Frequencies, and Noncovalent Interactions of Large Molecular Systems Parametrized for All spd-Block Elements (Z = 1–86), *J. Chem. Theory Comput.*, 2017, **13**, 1989–2009.
19 R. Ahlrichs, M. Bär, M. Häser, H. Horn and C. Kölmel, Electronic structure calculations on workstation computers: The program system turbomole, *Chem. Phys. Lett.*, 1989, **162**, 165–169.
20 F. Furche, R. Ahlrichs, C. Hättig, W. Klopper, M. Sierka and F. Weigend, Turbomole, *Wiley Interdiscip. Rev. Comput. Mol. Sci.*, 2014, **4**, 91–100.
21 A. D. Becke, Density-functional exchange-energy approximation with correct asymptotic behavior, *Phys. Rev. A*, 1988, **38**, 3098–3100.
22 J. P. Perdew, Density-functional approximation for the correlation energy of the inhomogeneous electron gas, *Phys. Rev. B*, 1986, **33**, 8822–8824.
23 S. H. Vosko, L. Wilk and M. Nusair, Accurate spin-dependent electron liquid correlation energies for local spin density calculations: a critical analysis, *Can. J. Phys.*, 1980, **58**, 1200–1211.
24 A. D. Becke, Density-functional thermochemistry. III. The role of exact exchange, *J. Chem. Phys.*, 1993, **98**, 5648–5652.
25 C. Lee, W. Yang and R. G. Parr, Development of the Colle-Salvetti correlation-energy formula into a functional of the electron density, *Phys. Rev. B*, 1988, **37**, 785–789.
26 J. P. Perdew and Y. Wang, Accurate and simple analytic representation of the electron-gas correlation energy, *Phys. Rev. B*, 1992, **45**, 13244–13249.
27 J. Tao, J. P. Perdew, V. N. Staroverov and G. E. Scuseria, Climbing the Density Functional Ladder: Nonempirical Meta--Generalized Gradient Approximation Designed for Molecules and Solids, *Phys. Rev. Lett.*, 2003, **91**, 146401.
28 C. Larriba and C. J. Hogan, Free molecular collision cross section calculation methods for nanoparticles and complex ions with energy accommodation, *J. Comput. Phys.*, 2013, **251**, 344–363.
29 C. Larriba-Andaluz and C. J. Hogan Jr., Collision cross section calculations for polyatomic ions considering rotating diatomic/linear gas molecules, *J. Chem. Phys.*, 2014, **141**, 194107.

30 P. Weis, F. Hennrich, R. Fischer, E. K. Schneider, M. Neumaier and M. M. Kappes, Probing the structure of giant fullerenes by high resolution trapped ion mobility spectrometry, *Phys. Chem. Chem. Phys.*, 2019, **21**, 18877–18892.

31 Chemotion Repository, https://www.chemotion-repository.net/welcome, (accessed 3 December 2024).
